# Quantitative Analysis of the KSHV Transcriptome Following Primary Infection of Blood and Lymphatic Endothelial Cells

**DOI:** 10.3390/pathogens6010011

**Published:** 2017-03-19

**Authors:** A. Gregory Bruce, Serge Barcy, Terri DiMaio, Emilia Gan, H. Jacques Garrigues, Michael Lagunoff, Timothy M. Rose

**Affiliations:** 1 Center for Global Infectious Disease Research, Seattle Children’s Research Institute, Seattle, WA 98101, USA; agregbruce@gmail.com (A.G.B.); serge.barcy@seattlechildrens.org (S.B.); jacques.garrigues@seattlechildrens.org (H.J.G.); 2 Department of Pediatrics, University of Washington, Seattle, WA 98101, USA; 3 Department of Microbiology, University of Washington, Seattle, WA 98101, USA; tdimaio@uw.edu (T.D.); lagunoff@uw.edu (M.L.); 4 Department of Global Health, University of Washington, Seattle, WA 98101, USA; emiliagan@hotmail.com

**Keywords:** transcriptome, herpesvirus, Kaposi’s sarcoma-associated herpesvirus, promoter, deep sequencing, RNAseq, latency

## Abstract

The transcriptome of the Kaposi’s sarcoma-associated herpesvirus (KSHV/HHV8) after primary latent infection of human blood (BEC), lymphatic (LEC) and immortalized (TIME) endothelial cells was analyzed using RNAseq, and compared to long-term latency in BCBL-1 lymphoma cells. Naturally expressed transcripts were obtained without artificial induction, and a comprehensive annotation of the KSHV genome was determined. A set of unique coding sequence (UCDS) features and a process to resolve overlapping transcripts were developed to accurately quantitate transcript levels from specific promoters. Similar patterns of KSHV expression were detected in BCBL-1 cells undergoing long-term latent infections and in primary latent infections of both BEC and LEC cultures. High expression levels of poly-adenylated nuclear (PAN) RNA and spliced and unspliced transcripts encoding the K12 Kaposin B/C complex and associated microRNA region were detected, with an elevated expression of a large set of lytic genes in all latently infected cultures. Quantitation of non-overlapping regions of transcripts across the complete KSHV genome enabled for the first time accurate evaluation of the KSHV transcriptome associated with viral latency in different cell types. Hierarchical clustering applied to a gene correlation matrix identified modules of co-regulated genes with similar correlation profiles, which corresponded with biological and functional similarities of the encoded gene products. Gene modules were differentially upregulated during latency in specific cell types indicating a role for cellular factors associated with differentiated and/or proliferative states of the host cell to influence viral gene expression.

## 1. Introduction

Deep sequencing of RNA transcripts (RNA-seq) has been used to examine the global cellular transcriptome at high resolution [1]. RNA-seq allows quantitation of the abundance and change in RNA transcripts yielding a transcriptome pattern that defines developmental stages or changes induced by specific treatments. Sophisticated and powerful computer programs have been developed to analyze and interpret the large RNA-seq datasets, which typically contain millions of short 50–100 bp sequence reads. Depending on the RNA-seq library construction approach, RNA transcripts will generate single RNA reads derived from one end of a transcript (unpaired library) or two paired RNA reads derived from both ends of a transcript (paired library). In addition, the RNA library can be constructed to either identify the DNA strand from which the RNA was derived (stranded library) or to provide no strand information (unstranded library). The set of RNA reads are aligned to a reference sequence, which allows the reads to be mapped to specific positions within the reference sequence. Specialized software, such as TopHat2 [2], can detect splicing events where a single RNA read maps to two or more different regions within the reference sequence. RNA splicing can be visualized using viewers, such as the Integrated Genome Viewer (IGV) [3], which shows a graphical representation of the spliced reads (Sashimi plot). RNA transcripts can be quantitated using specific algorithms and software by comparison of the mapped reads to known sequence features, such as exons, introns, RNA transcripts and protein coding sequences using a gene feature file (GFF) that lists the nucleotide start and stop positions of the features within the reference genome. Different mapping rules can be applied to determine whether a read maps to a feature, and some programs can utilize data from stranded libraries to determine whether a read maps to the DNA strand encoding the feature. Reads mapped to sequence features are normalized to the length of the feature (derived from the start and stop positions within the GFF file) and to the depth of the sequencing run (total mapped reads). Transcript abundance can then be expressed as RPKM (reads per kilobase per million mapped reads). For paired library analysis, transcript abundance can be expressed as FPKM (fragments per kilobase per million mapped reads), in which a fragment is defined by a pair of mapped reads. More recently, transcript abundance can be expressed as TPM (transcripts per million), in which mapped reads are normalized to the length of the transcript and then further normalized to the sequencing depth. This provides a more accurate relative determination of transcript abundance than RPKM or FPKM [4]. The choice of mapping tools and parameters is important for quantitating complex transcriptional data.

RNA-seq analysis has been used to analyze viral transcriptomes, including several large human herpesviruses [5,6,7,8,9,10,11,12]. These studies have revealed complex transcriptional processes yielding a dense pattern of overlapping transcription with multiple transcripts sharing common 5′ or 3′ ends, complex alternate splicing, antisense transcription and transcription of non-coding and micro RNAs (miRNA). However, the informatic approaches developed to analyze cellular transcriptomes are problematic when dealing with the transcription complexity in these large DNA viruses, especially for read and transcript quantitation [8,13]. In addition, the transcript annotations for these herpesviruses and the derived GFF sequence feature files are frequently inadequate to fully characterize the viral transcriptome. New approaches to resolve the transcript structure of high density genomes are critical to understanding virus biology and pathology [14].

The Kaposi’s sarcoma-associated herpesvirus (KSHV)/human herpesvirus 8 is a gammaherpesvirus, like the Epstein-Barr virus (EBV), and is associated with Kaposi’s sarcoma (KS) and two acquired immune deficiency syndrome (AIDS)-related lymphoproliferative diseases, pleural effusion lymphoma (PEL) and multicentric Castleman disease (MCD) [15]. The unique region of the KSHV genome is approximately 140,000 base pairs, encoding more than 80 genes. The linear arrangement of genes within the KSHV genome is similar to that seen in EBV, especially within the regions containing blocks of genes that are highly conserved among other herpesviruses [16]. KSHV contains a number of genes not found in EBV, which appear to be homologs of cellular genes that play important roles in immune evasion and pathology of the virus [17]. Herpesvirus genomes are complex with closely spaced genes and overlapping transcripts, sometimes from opposing DNA strands.

In KS tumors, KSHV infection is primarily detected in the characteristic KS spindle cells, which express multiple endothelial markers [18,19], whereas the infected PEL and MCD cells are of B-lymphocyte origin [20,21]. The majority of latently infected KS spindle and PEL cells express a number of KSHV genes in the viral latency locus at the right end of the genome, including K12 Kaposin (T0.7 RNA), ORF71 (K13; vFLIP), ORF72 (vCyc) and ORF73, the latency-associated nuclear antigen (LANA) [22,23]. The KSHV polyadenylated nuclear RNA (PAN; T1.1) encoded at the left end of the genome is the most highly expressed RNA in KS tumors and PEL cells, however, in situ hybridization studies suggest that it is mainly expressed in a limited number of cells that appear to be undergoing lytic replication [24,25]. The proliferating lymphoblastoid cells in MCD lesions are also infected with KSHV and express the KSHV latency-associated genes. However, the expression of additional KSHV genes associated with virus reactivation and lytic replication, such as vIL6, the viral homolog of interleukin 6, and ORF59, the DNA polymerase processivity factor, have also been detected in MCD [26,27]. 

In vitro, KSHV establishes latency in a wide variety of cell types, including endothelial, epithelial, fibroblast and lymphocyte lineages [28]. In the conventional program of KSHV latency first described in endothelial cells, de novo KSHV infection results in the majority of cells expressing KSHV LANA, with a small subpopulation, ~1% “spontaneously” entering the lytic cycle as evidence by expression of the lytic cycle marker ORF59 [29]. Later studies showed that KSHV infection induced early transient expression of ORF50, the replication transactivator (RTA), and a brief burst of RTA-induced lytic cycle gene expression, which disappeared by 8 hours post infection (hpi) [30]. The burst of lytic gene expression after de novo infection or spontaneous reactivation during long term latency is considered to be critical for the establishment of latency and maintenance of the KSHV infection in tumor cells in vivo [31]. The nature of this transient or spontaneous response suggests a delicate balance between latency and reactivation, which may differ between different cell lineages, stages of differentiation or position within the cell cycle. 

In most cell systems, it is necessary to reactivate the latent KSHV infection using chemical inducers such as the phorbol ester tetradecanoyl phorbol acetate TPA or the histone deacetylase (HDAC) inhibitor sodium butyrate. Sodium butyrate directly induces the KSHV RTA promoter and the butyrate-responsive element has been mapped to a Sp1-binding site [32,33]. KSHV RTA is the only viral gene that is necessary and sufficient for virus replication [34,35]. We have shown that the KSHV RTA promoter is highly active in cells, such as Vero and HEK293, which display the conventional KSHV latency after de novo infection [36]. Thus, cells with high basal levels of active Sp1 and other transcription factors that can activate the RTA promoter are poised to enter the lytic cycle. De novo infection of such cells results in the initial transient lytic gene expression. However, we and others have shown that LANA plays a role in blocking entry into the lytic cycle by directly inhibiting the RTA promoter [36,37]. Thus, LANA produced early during infection would inhibit the expression of RTA leading to the establishment of latency. Increased expression of KSHV RTA through induction by TPA and/or sodium butyrate or recombinant RTA expression systems would overcome the inhibitory effects of LANA or other viral and cellular factors, allowing the establishment of transcriptionally active euchromatin, robust lytic gene expression and viral replication.

In order to examine the pattern of KSHV gene expression at high resolution during the conventional latent KSHV infection in vitro, we utilized RNA-seq analysis to quantitate KSHV RNA transcripts in long-term latent infections in BCBL-1 PEL cells and compared this to primary latent infections in Vero monkey epithelial cells and human blood (BEC), lymphatic (LEC) and immortalized (TIME) endothelial cell cultures, 48 h post infection. We have utilized this RNA-seq data to identify and characterize typical KSHV gene transcripts. A detailed and comprehensive annotation of the KSHV genome was determined from the RNA-seq data and a simplified unique coding sequence (UCDS) GFF was developed to accurately quantitate gene transcripts from the complex KSHV genome structure.

## 2. Results

The NCBI reference sequence (RefSeq) for the KSHV genome was previously determined from overlapping cosmids from KSHV strain “GK18” present in a patient with a case of classic Kaposi’s sarcoma and contains 137,168 nucleotides (NC_009333). This record has been curated and contains numerous gene features, including predicted coding sequences (CDS), transcript sequences (mRNA), miscellaneous RNAs, repeat regions and regulatory features. The regulatory features include known TATA promoter sequences and poly(A) termination sequences, which indicate the approximate transcription initiation and termination sites. However, very limited information is available in the annotation for known or predicted spliced or alternate mRNA transcripts, and the regulatory features for most genes are not known or have not been annotated. Other KSHV genomes have been sequenced but the gene annotations are less complete than NC_009333.

### 2.1. Comparison of Conventional Latency in KSHV-Infected Cell Lines

In order to establish a system and protocol for accurately analyzing KSHV gene expression, we initiated a study to examine the KSHV transcriptome using RNA-seq analysis to globally characterize KSHV gene expression. Initially, we chose to examine the KSHV transcriptomes in latently infected cell cultures, avoiding the use of chemicals, such as TPA or sodium butyrate or exogenous protein inducers, such as recombinant ORF50 RTA, which could artificially induce gene transcription. The BCBL-1 PEL cell line was chosen as it was established from a patient with HIV disease and found to be naturally infected with KSHV. This cell line has been used extensively to study KSHV latency and reactivation since it displays a typical pattern of latent gene expression with a small percentage of cells undergoing spontaneous reactivation. BCBL-1 cells can be induced to high levels of lytic gene expression leading to production of infectious KSHV virions by treatment with phorbol esters or sodium butyrate. We also chose several different human endothelial cell lines, including blood (BEC), lymphatic (LEC) and Tert-immortalized (TIME) microvascular endothelial cells, which all yield typical latent KSHV infections [29,38]. In addition, we examined the latent KSHV infection in Vero monkey epithelial cells, which are commonly used to titer KSHV latent infections [39]. 

**Figure 1:**
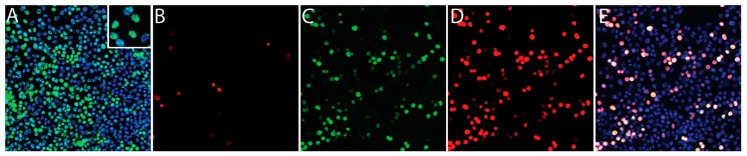
Characterization of a conventional de novo primary latent Kaposi’s sarcoma-associated herpesvirus (KSHV) infection. Vero cells were infected with gradient-purified KSHV for 48 h and then sequentially stained for the latency marker ORF73 (**A**; green) and the lytic marker ORF59 (**B**; red). KSHV-infected cell cultures were super-infected with baculovirus BAC50, which expresses the recombinant KSHV ORF50 transactivator, to induce lytic replication. The induced cells were fixed and sequentially stained for ORF50 (**C**; green) and ORF59 (**D**; red). Co-localization of the ORF50 and ORF59 staining is shown in panel **E** (white). Cell nuclei are stained with TO-PRO 3 (**A**,**E**; blue).

BEC, LEC, TIME and Vero cells were infected de novo with infectious KSHV that had been gradient purified from culture supernatant of sodium butyrate-treated BCBL-1 cells [39]. Three independent infections were performed for each of the BEC, LEC and TIME cell cultures on different days. A single infection of Vero cells was performed. Previous studies have shown that KSHV generally establishes latency 24 hpi [28,29,40], so we analyzed the infections after 48 h to ensure that latency was established. A portion of the infected cell cultures was fixed and stained for nuclear KSHV ORF73 LANA, a latency marker, and ORF59, a lytic marker, as described previously [29]. As shown for Vero cells in Figure 1A, greater than 95% of cells were latently infected and expressed LANA (green), visible as isolated nuclear dots with variable intensity across the culture (insert). Less than 2% of the Vero cells showed evidence of spontaneous reactivation with expression of ORF59 (Figure 1B, red). The KSHV-infected BEC, LEC and TIME cells showed similar staining with 79%–97% of cells staining for LANA and 1%–12% of cells staining for ORF59 (Table 1).

For comparison, the latent infection of Vero cells was reactivated by ectopic expression of KSHV ORF50 RTA transactivator using superinfection with the BAC50 RTA expression vector. Wide-spread reactivation of the latent KSHV infection was observed, with high levels of ORF59 expression detected in the BAC50 infected cells expressing KSHV ORF50 RTA, and the ORF50 and ORF59 staining co-localized (Figure 1C–E).

### 2.2. RNA-seq Analysis of KSHV Latent Transcriptomes

RNA was extracted from the long-term naturally infected BCBL-1 cells and from the experimentally infected Vero epithelial cells (Vero-K) and the triplicate infections of BEC, LEC, and TIME endothelial cell cultures (BEC-K1-3, LEC-K1-3, and TIME-K1-3) 48 h post infection. cDNA libraries were prepared from poly(A)-selected RNA and subjected to RNA deep-sequencing (RNA-seq) analysis on the Illumina platform. The Vero-K and triplicate LEC-K, BEC-K, TIME-K RNAs were sequenced for 50 bp from paired-end non-stranded libraries. Additional aliquots of the LEC-K1, BEC-K1, TIME-K1, and BCBL-1 RNA samples were sequenced for 50 bp from stranded libraries to distinguish sense and antisense RNA transcripts. Total reads ranged from 122 to 192 million for the paired-end libraries and 41 to 42 million for the stranded libraries, which were analyzed at a lower depth of sequencing (Table 1). Initially the stranded BCBL-1 RNA reads were compared to the non-stranded paired-end RNA reads from the Vero and triplicate BEC, LEC and TIME cell infections. The two read files from paired-end sequencing were combined and analyzed as unpaired. The read file from the BCBL stranded sequencing was initially analyzed as unstranded for comparison purposes. The reads were mapped onto the KSHV genome sequence (NC_009333) using Bowtie2 and TopHat2. A high level of KSHV reads were detected ranging from 1 million in the BCBL-1 cells to 9 million in the TIME cells (Table 1). These read depths are 10–100 fold higher than previous RNA-seq studies of KSHV gene expression. While the number of KSHV reads were similar between the triplicate infections of the BEC and LEC cells, one of the TIME infections (TIME-K1) had only 10% of the reads detected in the other two TIME infections (Table 1; Figure S1). Since the total read counts from the three infected TIME cell cultures were similar (120–140 million reads), it appeared that the KSHV infection in the TIME-K1 culture was substantially different, generating much fewer KSHV transcripts

**Figure 2:**
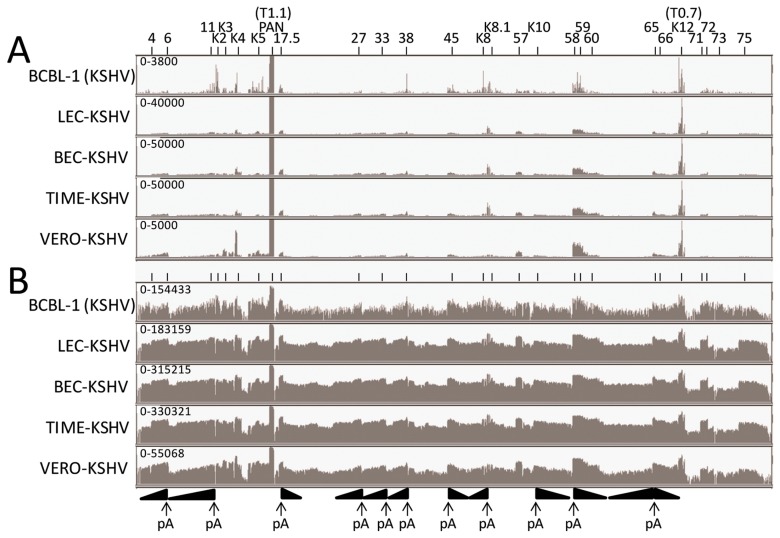
KSHV transcriptome analysis in different latently-infected cell types. RNA-seq analysis was performed on the BCBL-1 pleural effusion lymphoma (PEL) cell line, carrying a long-term latent KSHV infection and compared to de novo primary latent KSHV infections of human lymphatic (LEC), blood (BEC) and Tert-immortalized microvascular (TIME) endothelial cell cultures and the African Green monkey kidney cell line (Vero) 48 h post-infection (hpi). The vertical axis represents the number of reads aligned to each nucleotide position of the KSHV reference genome (NC_009333), and the visible axis range is shown on the left (**A** = linear scale - normalized to the level of the T0.7 read depth; **B** = log scale). KSHV ORFs with significant read depth are shown at the top. Obvious gradients of reads across gene clusters containing overlapping transcripts that terminate in a common poly(A) (pA) site are indicated at the bottom. Representative data from the second infection time point is shown for the triplicate LEC, BEC and TIME infections, i.e., LEC-K2, BEC-K2 and TIME-K2.

The RNA reads from the BCBL-1, LEC, BEC, TIME and Vero infections mapping to the complete KSHV genome (NC_009333) were visualized using the Integrated Genome Viewer (IGV) (Figure 2A—linear scale and Figure 2B—log scale). Two major RNA read peaks were observed in all of the infected cells, which mapped to the left-hand polyadenylated nuclear (PAN) RNA (T1.1) and the right-hand K12 RNA (T0.7). The PAN RNA read level was 10 fold higher than K12. To visualize other KSHV RNAs, the analysis window was scaled to the height of the K12 (T0.7) RNA reads, indicated at the left end of the analysis window (Figure 2A). This scale ranged from 3800 in the BCBL-1 sample to 50,000 in the BEC-K and TIME-K samples. Obvious RNA read peaks mapped to regions across the KSHV genome, as indicated in Figure 2A, with similar patterns in the different KSHV infected cell lines. The most prominent read peaks present in all cell lines mapped to ORFs K4, PAN, 17.5, K8.1, 57, 58, 59, and K12. Additional prominent RNA read peaks mapping to ORF K2, K5, 38, and K8 were observed in BCBL-1 cells. The RNA reads were also analyzed using a log-based scale, which auto-scaled to the PAN RNA read peak height, indicated at the left end of the analysis window (Figure 2B). The log-based scaling allowed analysis of the lower RNA peaks and revealed significant reads mapping to essentially the whole KSHV genome in all of the infected cells, with the major peaks as identified in Figure 2A, above. 

### 2.3. RNA-seq Analysis of KSHV Transcripts

**Figure 3:**
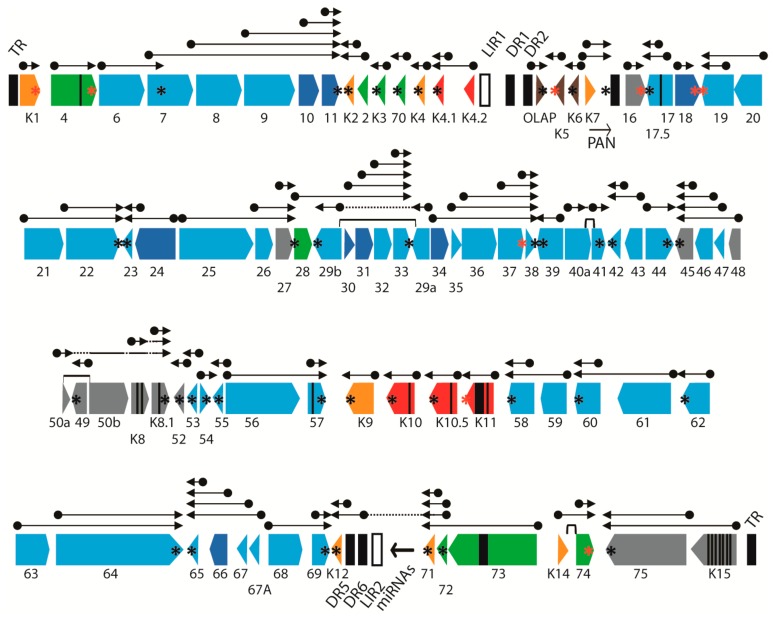
Map of the KSHV genome. The positions and transcription directions of the ORFs identified in the KSHV genome are shown. Vertical black lines within an ORF indicate splicing events or internal initiations, while longer range splices of protein-coding exons are indicated with bars. The positions of known polyadenylation (pA) sites of transcription termination are indicated with an asterix (AATAAA = black; ATTAAA = red) (see Table 2). The ORFs are color coded with regard to their conservation in other herpesvirus genomes, showing core genes conserved in the herpesvirus family (light blue), genes conserved in beta- and gamma- herpesviruses (dark blue), gammaherpesviruses only (grey), gamma-2-herpesviruses (green), RV1 and RV2 rhadinoviruses only (orange), RV1 rhadinoviruses only (red) and KSHV-only (brown). A current view of the complex pattern of gene transcription is indicated with arrows, which initiate at unique promoter elements (dots) and terminate at unique or shared pA sites. The terminal repeats (TR), direct repeats (DR) and long-inverted repeats (LIR) are indicated.

The KSHV genome, like other herpesvirus genomes, contains regions with multiple ORFs whose transcripts terminate at a common polyadenylation (poly(A)) site. Previous studies have mapped transcript termination sites and identified adjacent poly(A) signals, comprising in most cases the common signal “AATAAA” or the “ATTAAA” derivative [41,42] (Table 2). We have mapped these poly(A) signals onto the KSHV genome structure (Figure 3; “AATAAA”-black asterix, “ATTAAA”-red asterix). Interestingly, each of the major RNA peaks identified in our RNA-seq analysis, mapped adjacent to a known poly(A) site of transcript termination (Figure 2B). Obvious gradients of increasing RNA read depth occurred throughout the genome where multiple ORFs terminated at a common poly(A) site (Figure 2B).

### 2.4. Quantitation of RNA Transcripts Using Unique UCDS Features

To accurately quantitate transcripts across the whole KSHV genome, we utilized published information and our RNA-seq data from the KSHV-infected LEC, BEC, TIME, Vero and BCBL-1 cells to identify approximate positions of transcription start sites for the known KSHV ORFs and identify putative TATA-like promoter elements flanking these start sites upstream (Table 2). We also used published information to identify putative transcription termination sites with poly(A) signals for each KSHV ORF (Table 2). This data was used to develop a simplified gene feature file (GFF) containing unique UCDS features for known KSHV ORFs, RNAs and genomic regions that encode poly(A) RNA transcripts (Table 3). Each UCDS feature was designed to avoid overlap with other UCDS features, or any known overlap with 5′ and 3′ NC regions of other transcripts. UCDS features were also developed to detect RNA reads mapping to the sense strand of the long inverted and direct repeats in the KSHV genome. The novel UCDS GFF was used to quantitate KSHV transcripts across the entire genome for all five KSHV-infected cell types. Previously, RNA-seq-based quantitation of the KSHV transcriptome has utilized the coding sequence (CDS) features annotated in the KSHV reference genome (NC_009333) [9,10,43]. Our approach eliminated many of the problems of transcript quantitation of overlapping genes and transcripts resulting from the compact nature of the KSHV genome. In the subsequent sections, the RNA transcription patterns in specific regions of the KSHV genome were analyzed in detail to demonstrate the utility of using the UCDS gene features for transcript quantitation. The patterns of KSHV gene expression in the different latently infected cell types were then determined using the UCDS approach and compared to examine the effects of the host cell on KSHV latency. 

### 2.5. Quantitation of RNA Transcripts Is Confounded by Overlapping Transcripts

**Figure 4:**
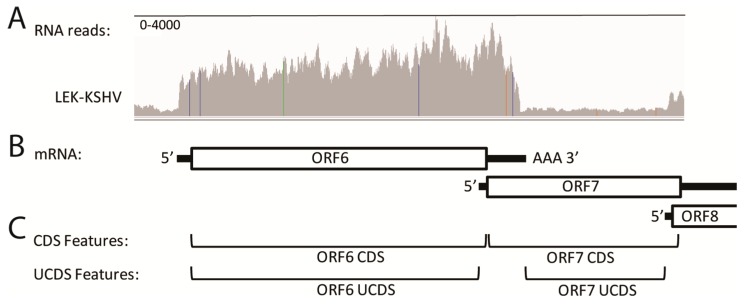
Quantitation of overlapping transcripts using simplified unique coding sequence (UCDS) features. RNA-seq analysis was performed on RNA from triplicate biological replicates of LEC, BEC and TIME cells isolated 48 h after primary infection with KSHV. Similar analysis was performed on a single culture of KSHV-infected Vero cells and on a long-term culture of KSHV-infected BCBL-1 cells. (**A**) The 50 bp RNA reads mapping to the KSHV ORF6 (single strand DNA binding protein homolog of HSV UL29), ORF7 (subunit of terminase homolog of UL28) and N-terminal domain of ORF8 (glycoprotein B homolog of UL27) were visualized using Integrated Genome Viewer (IGV) (representative data from the KSHV-infected LEC cells (LEC-K2) is shown). The range of the vertical axis (read count) is indicated. Allelic differences between the NC_009333 reference genome and the KSHV strain from BCBL-1 cells used for the infection are indicated as colored lines; (**B**) The position of the coding sequences (open boxes) and proposed extent of the 5’ and 3’ non coding regions of the ORF6, ORF7 and ORF8 mRNA transcripts are shown (see Table 2); (**C**) The extent of the coding sequence (CDS) features typically used to quantitate reads mapping to ORF6 and ORF7, as annotated in the NC_009333 genome, is indicated. The new unique UCDS features developed in this study to quantitate complex and/or overlapping transcripts in this region are shown (see Table 3).

An example of the problems associated with overlapping transcripts is shown at the left end of the genome where a moderate level of RNA-seq reads mapped to ORF6 in all five of the KSHV-infected cell types. These reads delineated the extent of the ORF6 mRNA transcript (Figure 4A; representative data from the LEC-K1 infection), extending from a putative transcription start signal (TSS) at bp 3132 to a transcription termination at bp 6974 (graphically visualized in Figure 4B). This was considerably different from the ORF6 annotations in the NC_009333 KSHV reference sequence, which indicated the ORF6 gene feature (gene) extending from bp 3179–17,026, and the coding sequence feature (CDS) extending from bp 3179–6577 (Figure 4C; CDS Features). Analysis of the sequence immediately upstream of the ORF6 putative TSS delineated by the RNA-seq reads revealed a “TATATAAA” TATA-like signal at bp 3090–3097 (Table 2). Analysis of the sequence flanking the putative termination site revealed an “AATAAA” poly(A)-signal at bp 6974–6979 that has been previously mapped [41,42]. Thus, the RNA-seq data indicates that the ORF6 mRNA transcript in these cells contains a short 5′ non-coding (NC) region (black line) upstream of the CDS (boxed) followed by a longer 3′ NC region (black line) terminating in a poly(A) site (Figure 4B). Thus, the 3′ end of the ORF6 NC region overlaps considerably with the ORF7 CDS feature (Figure 4B). While a significant number of RNA-seq reads map to the ORF 7 CDS in this overlap region, it is clear that these reads are derived from the 3′NC end of the highly expressed ORF6 transcript and not to the minimally expressed ORF7 transcript (Figure 4A,B). It is also known that the ORF8 transcript initiates downstream of a “TATTTAAA” like TATA-signal within the genomic region encoding ORF7, and the ORF7 and ORF8 CDSs overlap in this region (Table 2; Figure 4B).

To accurately quantitate the RNA-seq reads, we identified unique UCDS features for both ORF6 and ORF7 that would allow unambiguous RNA read quantitation (Figure 4C; Table 3). While the ORF6 UCDS feature defines the same sequence region as the ORF6 CDS feature of NC_009333, the ORF7 UCDS feature is truncated at both the 5′ and 3′ ends of the ORF7 CDS to avoid overlap with the ORF6 and ORF8 transcripts (Figure 4B,C; Table 3). To avoid additional overlap issues that could affect RNA read quantitation, the ORF7 UCDS feature was further truncated to start 50 bp downstream of the ORF6 UCDS and terminate 50 bp upstream of the TATA-like signal of ORF8. Separation of the UCDS features by the length of a single 50 bp RNA-seq read, permits the use of the “intersection_nonempty” setting in the HTSEQ software used to quantitate RNA reads to the UCDS features, as described in Materials and Methods. This setting allows all reads that partially or fully align to the UCDS feature to be counted, and no read was determined to be ambiguous due to overlap between two features. Currently, the promoter and TSS for ORF7 have not been identified. If further transcript analysis indicates additional overlap between the ORF6 coding sequence and the 5′ NC region of ORF7 then the ORF6 UCDS could be truncated at the 3′ end. For quantitation of reads from the paired-end sequencing libraries, we unlinked the paired-end reads to avoid further overlap issues. The relative expression of the KSHV transcripts was determined based on TPM (transcripts per million mapped KSHV reads) [4] with the average and standard deviation determined for the replicate infections. Normalization to the total mapped KSHV reads per sample allowed comparison of KSHV transcripts between samples that had variable levels of KSHV-infected cells.

**Figure 5:**
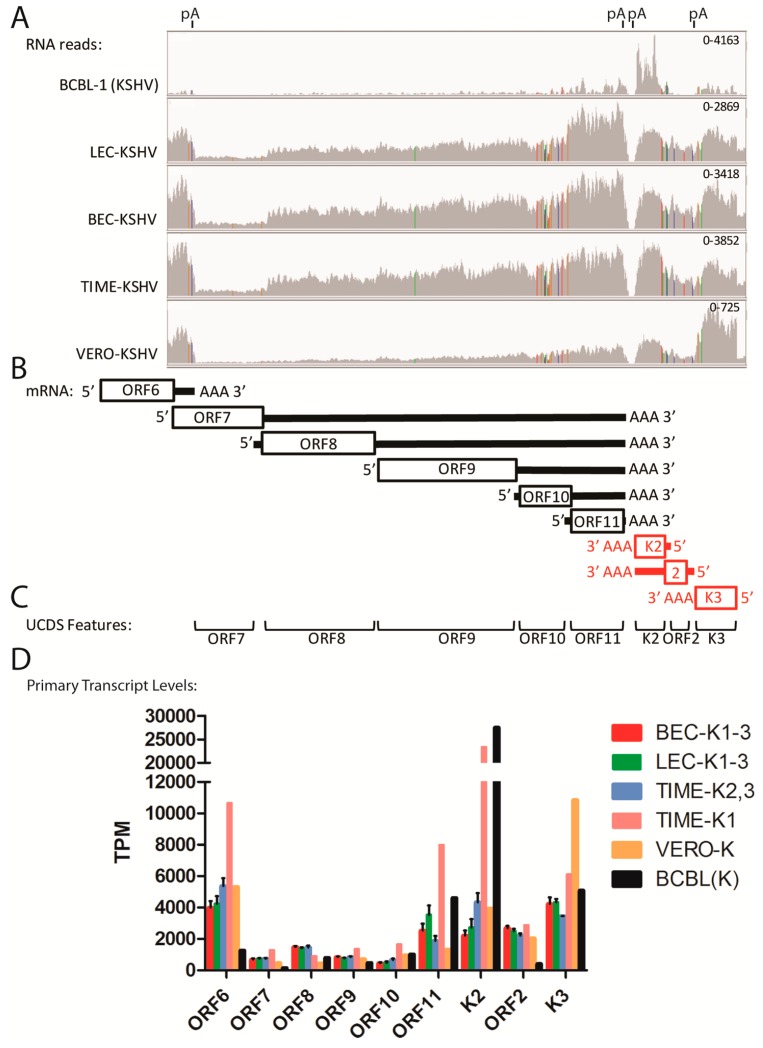
Quantitation of transcripts co-terminating in a common poly(A) site. (**A**) The RNA reads from the KSHV-infected cell cultures described in Figure 4 were mapped to the KSHV genome and the region from ORF6 to K3 was visualized using IGV (representative data from the KSHV-infected BCBL-1, LEC-K2, BEC-K2, TIME-K2 and Vero-K cells are shown). The positions of known poly(A) (pA) transcription termination sites are shown (see Table 2); (**B**) The proposed transcription pattern for this region is indicated. The “primary” transcripts for each 5′ proximal ORF are shown with the coding region indicated as an open box and the proposed poly(A) tail and 5′ and 3′ flanking regions indicated with a line (black = sense strand; red = antisense strand; see Table 2); (**C**) The non-overlapping UCDS features for quantitating transcripts are shown (see Table 3); (**D**) Reads mapping to the primary transcripts for each ORF were quantitated using the UCDS features and normalized (TPM), with the average and standard deviation determined for the replicate infections (see Table S1 for supporting data). The TIME-K1 infection is analyzed separately from the TIME-K2 and -K3 infections, as discussed in the text.

Using the new UCDS features, the average expression levels and variability of ORF6 in the triplicate independent BEC cell infections (BEC-K1-3) were found to be very similar to those seen in the infected LEC cells (LEC-K1-3) (4239 ± 12% TPM vs. 3997 ± 10% TPM, respectively (Figure 5D, Table S1). In contrast, the average ORF6 transcript level in the triplicate independent TIME cell infections was nearly 2 times higher with a much greater variability (7124 TPM ± 35%). Since the first replicate TIME cell infection (TIME-K1) was significantly different from the other TIME-K2 and -K3 replicate infections, as discussed above, it was then analyzed separately from TIME-K2 and -K3. This analysis showed that the ORF6 transcript levels in the duplicate TIME-K2 and -K3 infections were quite similar with an average of 5367 TPM ± 9%, similar to that seen in the Vero infection (5313 TPM) (Figure 5D; Table S1). Although the TIME-K1 infection had only 10% of the total KSHV mapped reads detected in the TIME-K2 and -K3 replicate infections, approximately twice as many reads mapped to the ORF6 UCDS feature (10,639 TPM)(Figure 5D; Table S1). In contrast, the ORF6 expression level in the BCBL-1 cells (1258 TPM) was ~30% lower than the levels in the LEC and BEC cells (Figure 5D; Table S1).

### 2.6. Quantitation of Overlapping Transcripts Terminating at Common Poly(A) Sites (ORF7-K3)

Many regions of the KSHV genome contain loci with multiple transcripts terminating at a common poly(A) site that further confound transcript quantitation. An example of this is seen in the region of the KSHV genome between ORFs 7 and K3 at the left end of the genome, which showed obvious gradients of RNA read depth across multiple genes. The RNA reads from this region were visualized by IGV and autoscaled with read heights indicated (Figure 5A). This allows a comparison of expression within this set of genes without regard to their relative contribution to the total KSHV gene expression in the different infected cells.

This region of the genome contains a number of genes implicated in lytic replication, including the terminase subunit (ORF7), glycoprotein B (ORF8), and DNA polymerase (ORF9). The transcripts for these genes terminate at a poly(A) site that is also common to transcripts for ORF10 and ORF11 (Figure 5A,B). The transcripts for ORFs K2 and 2 terminate at an adjacent poly(A) site on the opposite strand and are flanked by ORFK3, which has its own poly(A) site (Figure 5B). Obvious similarities were observed in the RNA reads from the KSHV-infected LEC, BEC and TIME cells, with an increase in the read depth from ORF7 to ORF11 and from ORF2 to ORFK2, in both cases with the highest read depth at ORFs 11 and K2 adjacent to the poly(A) sites (Figure 5A). Colored lines in the read pattern indicate allelic differences between the reference sequence of the GK18 KSHV strain (NC_009333) and the KSHV strain from BCBL-1 cells that was used to infect the cells (Figure 5A). The total viral reads mapping to each UCDS feature were quantitated using HTSEQ for the transcripts of ORFs 6–11, K2, ORFs 2 and K3 and normalized (TPM) (Figure S2 and Table S1). High levels of RNA reads mapped to the ORF11 UCDS in all infected endothelial cells (Figure 5A and Figure S2; Table S1), indicating that a primary monocistronic ORF11 transcript initiates from the designated ORF11 promoter (TATAT; bp 15,568) within the 3′ end of the sequence encoding ORF10 and terminates after the poly(A) signal immediately downstream of ORF11 (Figure 5B, Table 2). Due to the fact that multiple RNA transcripts derived from specific promoters upstream of each of the ORFs 7–10 also terminate at the ORF11 poly(A) site, RNA reads that map to the ORF11 UCDS can be derived from five different mRNA transcripts (Figure 5B). However, only the primary ORF11 monocistronic transcript initiated from the ORF11 promoter (see Figure 5B) is expected to be a major source of translated protein product through cap-dependent initiation. All other transcripts are polycistronic, in that they initiate further upstream from a different promoter adjacent to a primary 5′ proximal ORF. These polycistronic transcripts encode one or more distal ORFs in the 3′ end of the transcript that may or may not be translated to a protein product (Figure 5B). A similar situation is seen on the opposite DNA strand where ORFK2 is encoded by a primary monocistronic transcript which could initiate from a promoter element (TATTTTTAA; bp 18,122), immediately upstream of ORFK2, and terminate at a poly(A) site downstream of the ORFK2 (Figure 5B, Table 2). ORFK2 is present at the 3′ distal end of the primary ORF2 transcript, which is bicistronic and initiates at the ORF2 promoter (TATATAA; bp 18,570), upstream of ORF2. This transcript encodes ORF2 as the primary proximal ORF and terminates at the same poly(A) site as ORFK2.

In order to more accurately quantitate mRNA transcripts from promoters adjacent to each KSHV gene, we quantitated RNA reads mapping only to the primary transcript of the 5′ proximal ORF, which is presumed to produce the major translation product. Thus, the RNA reads mapping to the ORF7 UCDS are derived from the primary transcript of the ORF7 promoter in which ORF7 CDS is 5′ proximal (ORF7 primary transcript), and all of the reads mapping to the ORF7 UCDS would be derived from the ORF7 primary transcript, as indicated in Figure 5B. As discussed in the previous section, the ORF7 UCDS feature was truncated at the 5′ and 3′ ends so it would not detect reads derived from the 3′ non-coding region of the ORF6 transcript and the 5′ region of the ORF8 transcript. The expression levels of the primary transcripts of ORFs 6 and 7 determined using the ORF6 and ORF7 UCDS features are shown in Figure 5D for the different infected cell types. As seen in Figure 5B, reads mapping to the ORF8 UCDS feature would not only be derived from primary transcripts from the ORF8 promoter, in which ORF8 is 5′ proximal, but would also be derived from ORF7 primary transcripts, in which ORF8 is encoded downstream in a distal position. Therefore the levels of the ORF8 primary transcript were determined by subtracting the primary ORF7 transcripts from the total transcripts mapped to the ORF8 UCDS feature, as indicated in Table 3 and Table S1. A similar strategy was used to quantitate the primary transcripts of ORFs 9–11 in an ordered hierarchical fashion (Table 3; Table S1). In each case, the UCDS feature for each gene was truncated to avoid overlap with the 5′ end of the putative mRNA transcript from the adjacent gene, eliminating the possibility of a single 50bp read mapping to two UCDS features.

Thus, while the total reads mapping to the ORFs 7–11 UCDS features increased stepwise from ORF7 to ORF11 for all the infected cell types, with transcript levels from 158 (ORF7) to 13,143 TPM (ORF11) (Figure 5A; Table S1; Figure S2), the levels of the primary transcripts from the associated promoter for each ORF were variable (Figure 5D). In the endothelial infections, the primary transcripts from the ORF8 promoter (1397–1478 TPM) were more abundant than the primary transcripts from the promoters of ORF9 (766–844 TPM) and ORF10 (451–649 TPM) (Table 1; Figure 5D and Figure S2A–C). Of the genes in this locus, the ORF11 primary monocistronic transcript was expressed at the highest level (1875–3528 TPM), with higher levels in the unusual TK1 infection and in the BCBL-1 cells (7982 and 4618 TPM, respectively).

Similarly, for the ORF2 and ORFK2 locus, higher levels of reads mapped to the polyA-proximal ORFK2 compared to polyA-distal ORF2 in all cells (Table S1). In these cases, the ORF2 UCDS reads derived only from the bicistronic ORF2 transcript initiating from the ORF2 promoter, while the ORFK2 reads derived from both the bicistronic ORF2 transcript and the monocistronic ORFK2 transcript initiating from the ORFK2 promoter downstream (Figure 5B). The levels of the ORFK2 primary transcript were determined by subtracting the contribution of the primary ORF2 transcripts from the total transcripts mapped to the ORFK2 UCDS feature (Table S1; Figure S2A,B). This analysis revealed similar levels of primary transcripts for both ORFK2 and ORF2 in the endothelial cells, ranging from 2,205 to 4890 TPM (Figure 5D; Table S1). In contrast, very elevated levels of ORFK2 transcripts were observed in both the unusual TK1 infection and in the BCBL-1 cells (23,292 and 27,509 TPM, respectively) (Figure 5D; Table S1). The TK1 infection also showed unusually high levels of primary transcripts for ORF6 and ORF11 (Figure 5D; Table S1).

### 2.7. Gene Expression in the Divergent Locus B and PAN Region

**Figure 6:**
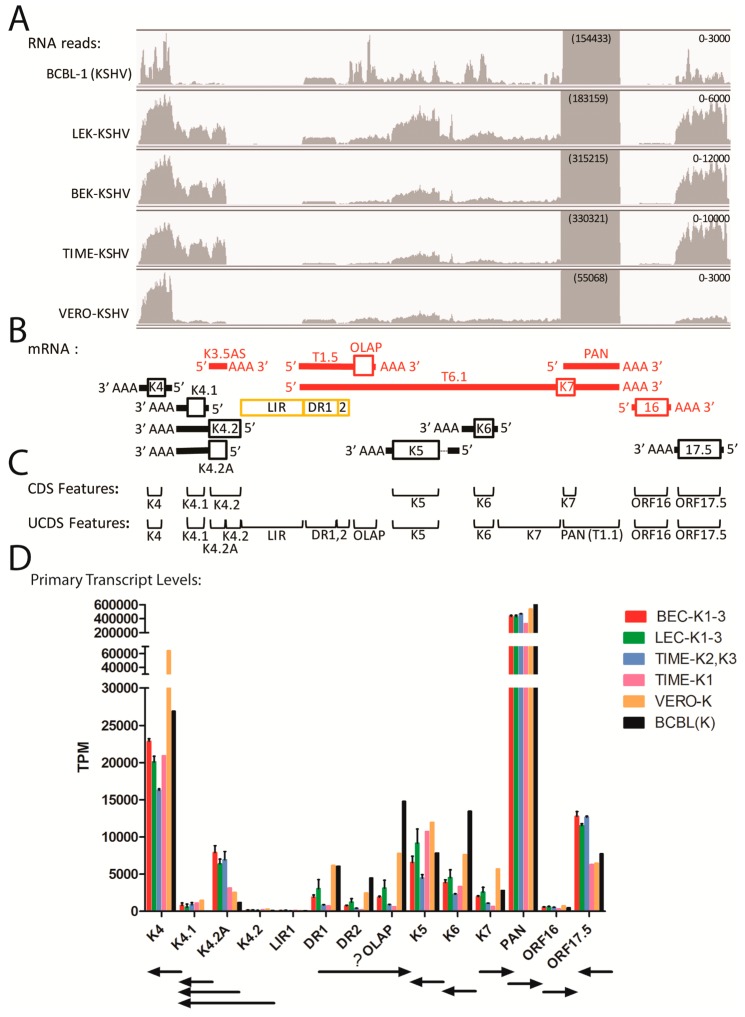
Quantitation of reads mapping to the lytic PAN region (ORFK4-ORF17.5). (**A**) The RNA reads from the KSHV-infected cell cultures described in Figure 4 were mapped to the KSHV genomic region from ORFs K4 to 17.5 and visualized using IGV (representative data from the KSHV-infected BCBL-1, LEC-K2, BEC-K2, TIME-K2 and Vero-K cells are shown). The range of the vertical axis (read count) is indicated on the right and the depth of reads for the PAN RNA is indicated in parenthesis; (**B**) the proposed transcription pattern for this region is shown (red and black coloring differentiate transcripts derived from the different DNA strands; see Table 2). The position of a ORFK4.2A truncated transcript encoding only the C-terminal half of ORFK4.2 is indicated, as are the positions of the direct repeats DR1 and DR2 and the long inverted repeat LIR1; (**C**) the extent of the typical CDS features from the NC_009333 KSHV reference sequence are shown in comparison to the non-overlapping UCDS features developed in this study (see Table 3); (**D**) Reads mapping to the primary transcripts for each ORF were quantitated using the UCDS features and normalized (TPM) and the average and standard deviation was determined for the replicate infections (see Table 3 and Table S2 for supporting data). The general extent and direction of each transcript is shown.

Other transcript quantitation issues were observed in the region between ORFs K3 and 17.5 in the divergent locus B at the left end of the KSHV genome, which contains a number of viral homologs of cellular genes, including the interleukin-8-like chemokines ORFs K4 (vCCL-2), K4.1 (vCCL-3), and K6 (vCCL-1), the K3 and K5 E3 ubiquitin ligase membrane proteins, and the K7 IAP-like and ORF16 Bcl-2-like apoptosis inhibitors (Figure 3). ORFs K7 and 16 flank the major polyadenylated nuclear RNA PAN (T1.1), which was the most highly expressed transcript detected in the five cell types studied (Figure 2). A simplified pattern of transcription in this region is shown in Figure 6B, with transcripts derived from both DNA strands (red and black). The PAN and ORFK7 transcripts share a common poly(A) transcription termination site, as do ORFs K4.1 and K4.2. The other transcripts utilize unique termination sites. This region contains the left origin of lytic replication (LIR) and two regions of direct repeats, DR1 and DR2 (Figure 3), from which several novel transcripts have been identified but not completely characterized [44,45].

Extremely high levels of reads mapped to the genomic region encoding the lytic cycle associated T1.1 PAN RNA transcript (Figure 6A). These reads are mistakenly identified as derived from the ORFK7 transcript using the typical NC_009333 GFF for mapping, as the ORFK7 CDS feature overlaps the PAN transcript and PAN itself is not a CDS feature in the NC_009333 GFF since it is considered to be non-coding (Figure 6C). To rectify this problem, we developed UCDS features to separately quantitate reads mapping specifically to either PAN RNA or the K7 transcript by setting the PAN UCDS feature to the limits of the PAN RNA and limiting the ORFK7 UCDS feature to the 5′ non-coding region of the ORFK7 transcript upstream of PAN RNA (Figure 6C). HTSEQ analysis using these UCDS features revealed very high level of RNA reads mapping to PAN (ranging from 327,965 TPM in the TIME-K1 infection to 650,560 TPM in BCBL-1 cells (Figure 6D and Figure S3; Table S2). This transcript was the most abundant transcript detected in BCBL-1 cells and in the different cell types latently infected with KSHV. In contrast, the level of reads mapping only to the ORFK7 transcript were quite low in comparison, ranging from 622 (TIME-K1) to 5694 TPM (VERO-K) (Figure 6D and Figure S3; Table S2).

Substantial numbers of reads mapped to the ORFK4 UCDS feature in all cell types, with transcript levels ranging from 16,272 TPM in TIME-K2,-K3 infections to 63,647 TPM in the VERO infection, (Figure 6A,D and Figure S3; Table S2). ORFK4 encodes the viral CC chemokine homolog (vCCL-2) implicated in endothelial survival [46]. High levels of reads mapped to the 3′ end of the ORFK4.2 transcript in all of the infected endothelial cells, with essentially no reads mapping to the proposed 5′ end (Figure 6A). To quantitate transcripts from this region, we developed a set of non-overlapping UCDS features (Figure 6C). Separate ORFK4.2 and ORFK4.2A UCDS features were defined to quantitate the transcript from the putative ORFK4.2 promoter (TAATTTAT; bp 23,099) encoding the full-length 182 aa ORFK4.2 and the transcript from the putative ORFK4.2A promoter (TATTAAA; bp 22,871) predicted to encode a novel 101 aa C-terminal end of ORFK4.2 containing only two membrane spanning domains. The transcripts for ORFs K4.2, K4.2A and K4.1 terminate at a common poly(A) site (Figure 6B). A substantial level of reads mapped to the truncated ORFK4.2A transcript in the infected endothelial cells, with transcript levels ranging from 3128 TPM in TIME-K1 to 7896 TPM in the BEC-K1-3 infections (Figure 6D). Only low levels (1189 TPM) were detected in BCBL-1 cells. No full length ORFK4.2 transcripts were detected in any of the five cell types (Figure 6D and Figure S3; Table S2). A moderate level of reads also mapped to the UCDS feature for ORFK4.1, the vCCL-3 chemokine homolog, in the infected endothelial cells, however, the majority of these reads were derived from the bicistronic ORFK4.2A/K4.1 transcript, in which K4.1 is the distal ORF. Thus, the endothelial cells expressed only low levels of the ORFK4.1 monocistronic primary transcript produced from the putative ORFK4.1 promoter (TAATTTTATAA; bp 22,532), while BCBL-1 cells were essentially devoid of this transcript (Figure 6D; Table S2).

Moderate levels of reads mapped to the ORFK5 and ORFK6 UCDS features in all five cell types (Figure 6D and Figure S3; Table S2). BCBL-1 cells had a high level of ORFK6 transcripts (13,459 TPM), nearly 3 times that detected in the infected endothelial cells. Variable levels of reads mapped to the KSHV genome sequence within the DR1 and DR2 repeat regions, with the highest levels in the BCBL-1 and Vero cells. These reads are derived from the T1.5 and T6.1 transcripts antisense to ORFK6, as indicated in Figure 6B (red). Very few reads mapped to ORF16 (<800 TPM) in all of the infected cell types.

A notable level of variation between the reads mapping to the genomic region containing DR1, DR2, OLAP, ORFK5, ORFK6 and ORFK7 UCDS features was observed in the three biological replicates of the KSHV infection of the LEC cells, with standard deviations of 21%–40%, nearly 10 times higher than that seen in the flanking ORFs (Table S2, highlighted in yellow) or in other ORFs throughout the genome (Tables S1 and S3–S7). This suggests that the transcription of this region is variable in the different replicate infections and can be influenced by the conditions of the infection or the status of the target cells being infected.

### 2.8. Quantitation of Overlapping Transcripts for ORFs 17 and 17.5

**Figure 7:**
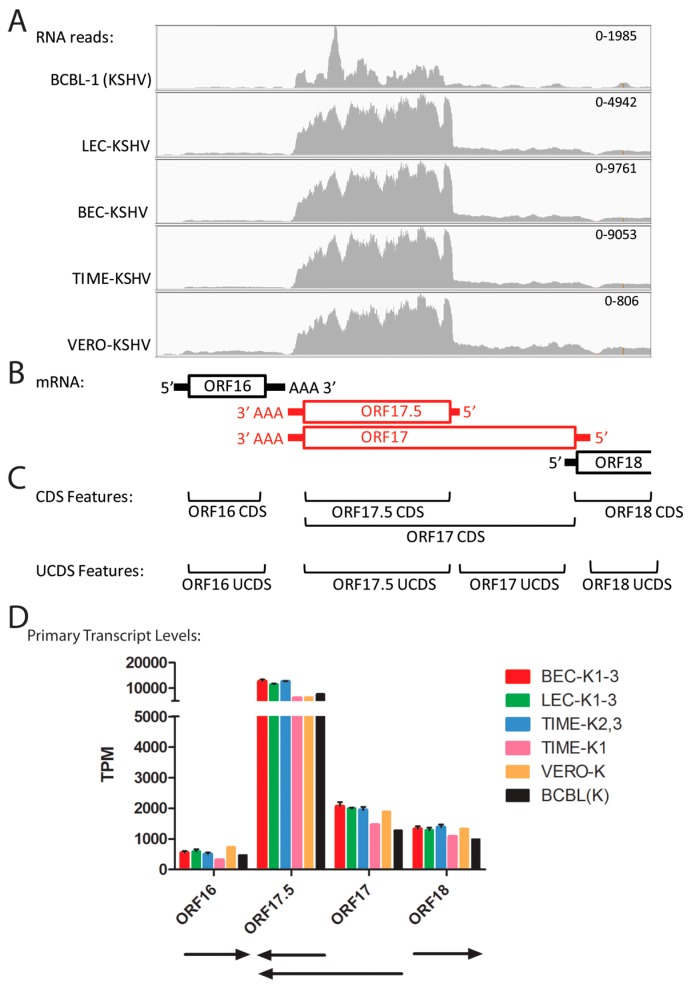
Quantitation of reads mapping to overlapping ORFs (ORF17.5–ORF17). (**A**) The RNA reads from the KSHV-infected cell cultures were mapped to the KSHV genomic region from ORFs 16 to 18 and visualized using IGV (see Figure 4 legend); (**B**) The proposed transcription pattern for this region is indicated; (**C**) The overlapping CDS features from the KSHV reference genome NC_009333 and the non-overlapping UCDS features developed in this study for quantitating transcripts are shown; (**D**) Reads mapping to the primary transcripts for each ORF were quantitated using the UCDS features and normalized (TPM) and the average and standard deviation were determined for the replicate infections (see Table 3 and Table S3 for supporting data).

ORF17.5 encodes a capsid scaffold protein of 288 amino acids from a transcript that initiates at bp ~31,807 downstream of a “TATTTAAA” TATA-like promoter element (Table 2). ORF17 encodes the ORF17.5 scaffold protein at the C-terminus of a larger 534 amino acid protein containing a viral protease at the N-terminus. The ORF17 transcript initiates at bp 32,524 and terminates at bp 30,920, as depicted in Figure 7B. To eliminate overlap problems, we developed an ORF17.5 UCDS feature to quantitate reads mapping to both ORF17.5 and the C-terminal domain of ORF17, and an ORF17-specific UCDS feature to quantitate reads mapping only to the N-terminal domain of ORF17 (Figure 7C). Primary ORF17.5 transcripts are then quantitated by subtracting the contribution of the ORF17 transcripts. A low level of reads mapping to the ORF17 transcript was observed in all of the infected cells, ranging from 1276 to 2069 TPM) (Table S3). In contrast, the primary ORF17.5 transcripts were ~6 fold higher and ranged from 6274 in TIME-K1 cells to 12,777 TPM in the BEC-K1-3 infections (Figure 7D; Table S3).

### 2.9. Quantitation of Spliced Transcripts, ORFs 50/K8/K8.1

**Figure 8:**
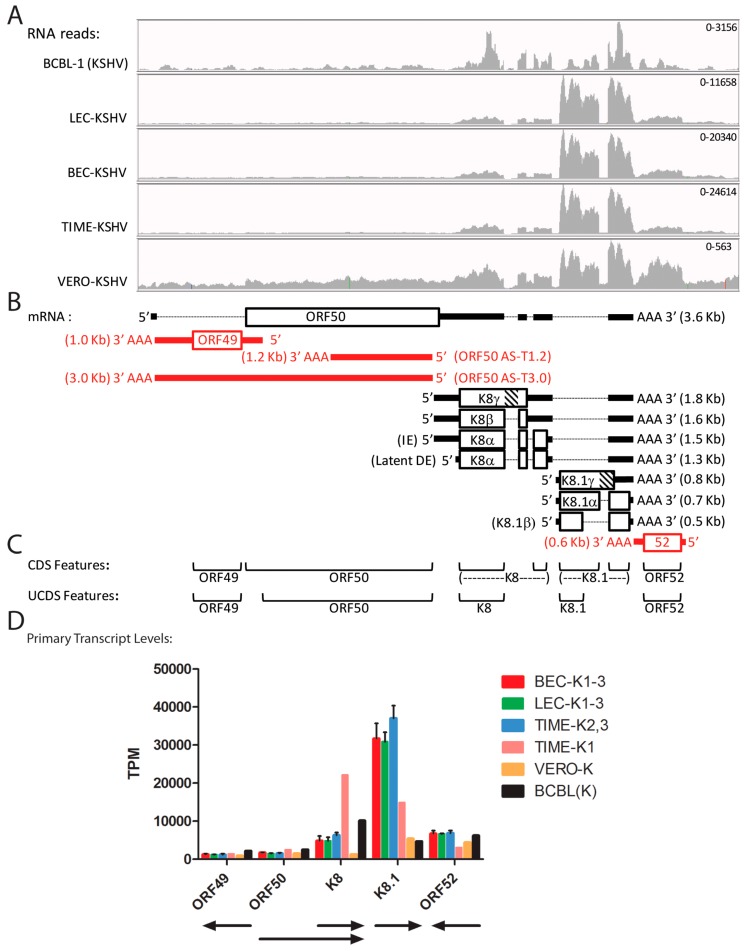
Quantitation of reads mapping to spliced lytic transcripts (ORF50-ORFK8.1). (**A**) The RNA reads from the KSHV-infected cell cultures were mapped to the KSHV genomic region from ORFs 49 to 52 and visualized using IGV (see Figure 4 legend); (**B**) the proposed transcription pattern for this region is indicated (black = sense strand; red = antisense strand). Intron regions from spliced transcripts are shown as thin dotted lines. The proposed non-coding antisense (AS) ORF50 transcripts T1.2 and T3.0 are shown. IE = immediate early; DE = delayed early; (**C**) The overlapping CDS features from the KSHV reference genome NC_009333 are shown with one spliced transcript each for ORFs K8 and K8.1, as indicated. The non-overlapping UCDS features developed in this study for quantitating transcripts are shown. The ORFK8 and ORFK8.1 UCDS features target the 5′ exons common to all spliced and unspliced transcripts; (**D**) Reads mapping to the primary transcripts for each ORF were quantitated using the UCDS features and normalized (TPM) with the average and standard deviation for the replicate infections (see Table 3 and Table S4 for supporting data). The general extent and direction of each transcript is shown.

The transcription pattern across the ORF50, ORFK8 and ORFK8.1 locus has been previously analyzed in detail, and a large set of overlapping spliced transcripts has been identified, as summarized in Figure 8B [47,48]. Transcription from this locus has been extensively used to detect lytic replication, as ORF50 is the replication transactivator (RTA), considered to be the central regulator of the lytic replication cycle, ORFK8 (K-bZIP) is an early protein implicated in KSHV replication, and ORFK8.1 is a late lytic cycle enveloped glycoprotein on the infectious virion. Overlapping spliced transcripts are produced from promoters upstream of ORF50 (~71,627 bp), ORFK8 (~74,592 bp) and ORFK8.1 (~75,966 bp), which all terminate after a single poly(A) signal (76,813 bp) (Figure 8B; Table 2). CDS features identifying single spliced transcripts for ORFK8 and ORFK8.1 are annotated in the NC_009333 reference sequence (Figure 8C). Initially, to quantitate transcripts encoding all ORF50, ORFK8 and ORFK8.1 isoforms, we developed non-overlapping UCDS features for ORFs 50, K8 and K8.1 (Figure 8C). The ORFK8 and ORFK8.1 UCDS features correspond to the large initial exons present in all of the characterized ORFK8 and ORFK8.1 transcripts, respectively. Since the ORF50 primary transcript is bicistronic with ORFK8 and overlaps the ORFK8 primary transcript, the level of the primary ORFK8 transcript was determined by subtracting the contribution of the primary ORF50 transcripts, as indicated in Table 3 and Table S4.

A major problem for quantitating the primary ORFK8.1 transcript is due to the common usage of the terminal exon in all of the ORF50, ORFK8 and ORFK8.1 spliced and unspliced transcripts (Figure 8B). Since the ORFK8.1 CDS feature, as annotated in the NC_009333 reference sequence, includes both the 5′ ORFK8.1 specific and the 3′ ORF50/K8/K8.1 common exon (Figure 8C), it cannot be used to quantitate ORFK8.1 specific transcripts, especially if high levels of the spliced ORF50/K8 transcript are present. The new ORFK8.1 UCDS feature, in contrast, targets only the initial ORFK8.1 specific 5′ exon, and thus avoids this problem. However, this UCDS feature would also detect the large unspliced transcripts generated from the ORF50 and ORFK8 promoters. 

Using the new UCDS features, high levels of the ORFK8.1-specific primary transcript (ranging from 30,682 to 37,000 TPM) were detected in the infected BEC-K1-3, LEC-K1-3 and TIME-K2,3 cells, indicative of late lytic cycle gene expression (Figure 8D; Table S4). Six fold less K8.1 transcripts (4686 TPM) were detected with this feature in BCBL-1 cells, suggesting a more latent phenotype. The TIME-K1 infection was intermediate (14,859 TPM). In contrast, the BCBL-1 cells had higher levels of K8 transcripts (12,701 TPM) than the BEC-K1-3, LEC-K1-3, and TIME-K2, 3 infections (6295–7841 TPM) (Figure 8D; Table S4). Unexpectedly, much higher levels of ORFK8 transcripts were detected in the TIME-K1 infection (24,601 TPM), 3–4 fold higher than the BEC, LEC and other TIME cell infections and twice that detected in BCBL cells (Figure 8D; Table S4). Of note, quantitation of the ORFK8.1 transcripts in the BCBL-1 and TIME-K1 cells using the conventional K8.1 CDS feature targeting the 3′ ORF50/K8/K8.1 common exon (Figure 8C) would have mistakenly identified the high level of ORFK8 reads as a high level of ORFK8.1. 

Consistent low levels of reads mapped to both the ORF49 and ORF50 UCDS features in all five of the infected cell types, ranging from 884/1470 TPM in Vero cells to 1412/2477 TPM in TIME-K1 cells, for ORF49 and ORF50 respectively (Figure 8D and Figure S4; Tables S4 and S5). However, as shown in Figure 8B, transcripts antisense to ORF50 (T1.2 and T3.0; ORF50AS) have been previously identified [48]. Thus, to distinguish reads mapping to the ORF50 sense and antisense transcripts, we analyzed the reads from the stranded RNA-seq libraries that had been prepared from the infected BCBL-1, BEC-K1, LEC-K1 and TIME-K1 cells (Table 1). BOWTIE2 and TopHat2 were used to align the RNA reads from the stranded libraries to the sense and antisense sequences corresponding to the ORF50 UCDS feature. This analysis revealed that only 55% of the reads mapping to the ORF50 UCDS feature were derived from the sense-strand ORF50 transcript in BCBL-1 cells (Table S6). The remaining 45% of the transcripts mapped to ORF50 antisense transcripts. In the infected LEC, BEC and TIME cells, 65%–69% of the reads mapped to the ORF50 sense-strand transcript, while 31%–35% mapped to the antisense transcripts. Thus, the level of sense-strand primary ORF50 transcripts is approximately 50% less than that depicted in Figure 8D.

**Figure 9:**
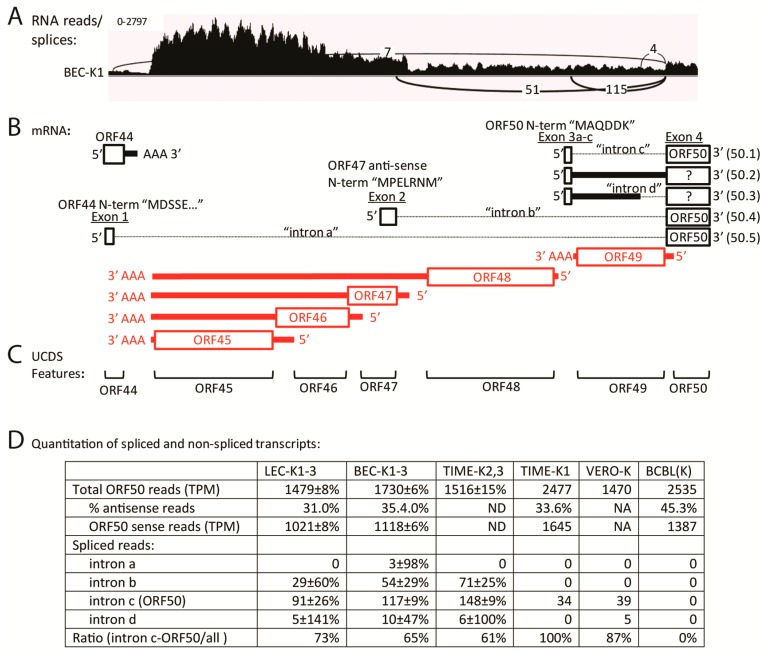
Alternate splicing of ORF50 transcripts. (**A**) An IGV Sashimi plot from TopHat2 analysis of split reads mapping across intron junctions of alternate ORF50 transcripts (representative data from the BEC-K1 infection is shown). Total RNA read depth is shown across the region 5′ of the ORF50 coding sequence. The position and number of split reads mapping across exons are indicated. (**B**) Positions of characterized antisense strand (red) ORFs 45–49 transcripts and sense strand (black) ORF44 and ORF50 transcripts, and upstream sequences alternately spliced to the major ORF50 coding exon are shown. Proposed transcripts (50.1–50.5), introns “a–d” and exons “1–4” are labeled. Exon 1 encodes the N-terminal 136 aa of ORF44 spliced in-frame to the C-terminal 685 aa of ORF50 RTA (exon 4). Exon 2 is derived from a sequence antisense to ORF47, which is spliced to ORF50 exon 4. The 5′ end of this transcript is uncharacterized, as is its coding potential. Exon 3A (transcript 50.1) encodes the 5′ proximal N-terminal sequence “MAQDDK” spliced to the C-terminal 685 aa of ORF50 RTA in exon 4. This transcript encodes the classical ORF50 RTA. Exon 3B and exon 3C encode the 5′ proximal N-terminal peptide “MAQDDKVKIDLFIVY” from the unspliced (50.2) and spliced (50.3) transcripts with possible uncharacterized distal ORFs, including a truncated ORF50 RTA (indicated ?); (**C**) The non-overlapping UCDS features are indicated; (**D**) The number of sense and antisense reads mapping to the ORF50 UCDS feature are shown, and the number of split reads detected by TopHat2 are indicated for the different KSHV infections. The mean and standard deviation for replicate analyses are given. Quantitation of reads mapping to the UCDS features of ORFs 44–50 is provided in Table S6.

The characteristic ORF50 transcript (herein designated 50.1), encoding the ORF50 isoform 1, is spliced from a small exon encoding the six N-terminal amino acids “MAQDDK” to the larger exon downstream of ORF49, which encodes the majority of the ORF50 coding sequence (Figure 8B and Figure 9B). To determine whether any correctly spliced ORF50.1 transcripts were expressed in the five KSHV-infected cell types, we examined the TopHat2 analysis of the RNA-seq reads and compared this with the total reads mapping to the ORF50 UCDS feature. TopHat2 examines the RNA reads that don’t initially map to the target genome sequence, and detects reads ab initio that are split across exon boundaries, indicating a splice junction. Due to the directionality of the splice junction, the sense of the spliced transcript is determined by the algorithm. A representative IGV Sashimi plot for the BEC-K1 infection (paired-end library) is shown, indicating depths of unspliced reads (vertical axis) and numbers and positions of spliced reads (Figure 9A) in relation to the proposed transcripts (Figure 9B). TopHat2 detected an average of 91, 117 and 148 reads split across the ORF50 splice junction of intron “c”, characteristic of transcript 50.1, in the infected LEC-K1-3, BEC-K1-3 and TIME-K2,3 cells, respectively (Figure 9D). The infected Vero and TIME-K1 cells had 39 and 34 split reads, respectively. In contrast, no ORF50 spliced reads were detected in the latently infected BCBL-1 cells, which had only a low level of sense-strand ORF50 transcripts (1387 TPM). 

TopHat2 analysis revealed the presence of additional ORF50 splicing events, in which alternate upstream exons in ORF44 and antisense to ORF47 were spliced to the major exon encoding the majority of the ORF50 coding sequence, as depicted in Figure 9A,B. The transcript 50.5, in which intron “a” is removed, is predicted to encode a novel ORF50 isoform (isoform 5—based on the numbering scheme proposed in [49]) containing the N-terminal 136 aa of ORF44 (exon 1) spliced to the C-terminal 685 aa of ORF50 (exon 4). Only low levels of split reads identifying this transcript were detected in the BEC infections (Figure 9D). The transcript 50.4, in which intron “b” is removed, has been recently identified in TPA-induced BCBL-1 cells [49] and is predicted to encode ORF50 isoform 4, with the N-terminal sequence “MPELRNM” from a region antisense to ORF47, which is spliced to the C-terminal 685 aa of ORF50. A moderate level of split reads identifying this transcript were found in the LEC-K1-3, BEC-K1-3 and TIME-K2, 3 infections (29–71 reads), but not in the TIME-K1, VERO or uninduced BCBL-1 infections (Figure 9D). TopHat2 analysis also identified alternate splicing of ORF50 exon 3, in which intron “d” is removed in transcript 50.3. This transcript would encode a small ORF of 15 aa initiating with the 6 N-terminal amino acids “MAQDDK” of ORF50 isoform 1, which would not be spliced to the C-terminal 685 aa of ORF50. Although the TopHat2 analysis revealed that the splicing generating the 50.1 transcript encoding the classic ORF50 isoform 1 was 2–3 times more prevalent than the splicing for the other ORF50 isoforms, the level of this spliced transcript was quite low in the infected endothelial cells and it was not detected in the BCBL-1 cells (Figure 9D). RNA-seq analysis of the stranded libraries for BCBL-1, LEC-K1, BEC-K1 and TIME-K1 revealed only a low level of reads mapping antisense to ORF49 (58–112 TPM) providing minimal evidence for the unspliced transcript 50.2. No evidence for alternately spliced ORF50 transcripts encoding RTA isoforms 2 and 3, which were observed previously in TPA-induced BCBL-1 cells [49], were observed in any of the latently infected cells. 

### 2.10. Characterization ORF57 Alternate Splicing

**Figure 10:**
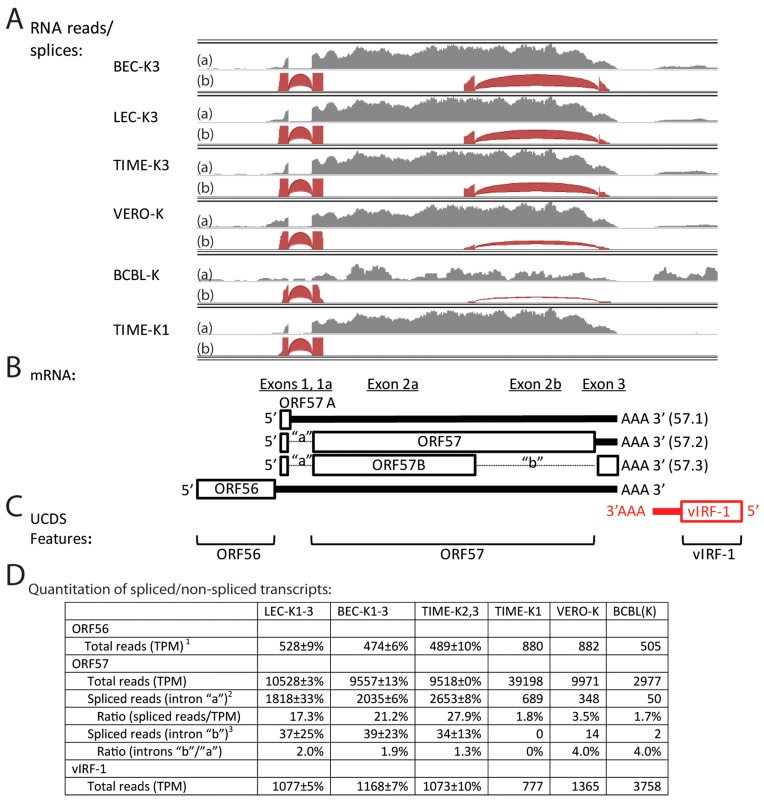
Alternate splicing of ORF57 transcripts. (**A**) RNA reads from the KSHV-infected cell cultures were mapped to the KSHV genomic region from ORF56 to vIRF-1(K9), and representative data for spliced and un-spliced reads were visualized using IGV (see Figure 4 legend). Depth of total mapped reads (dark gray-top section (a)) and spliced reads (brown-bottom section (b)) are shown on the vertical axis. The number of split reads is indicated by an arc from the beginning to the end of the junction and the height and thickness of the arc are proportional to the depth of read coverage up to 50 reads; (**B**) Proposed mRNA transcript structure; the unspliced 57.1 transcript encodes the 28 aa ORF57A as a 5′ proximal ORF; the single spliced 57.2 transcript encodes the full length 455 aa ORF57; the double spliced 57.3 transcript encodes a truncated 299 aa ORF57B with a novel C-terminal domain (see Figure S5 for encoded sequences). ORFs on the sense strand are shown in black, while ORFs on the complementary strand are shown in red; (**C**) position of UCDS features for read quantitation; (**D**) Total reads mapping to the UCDS features were quantitated and normalized (TPM). Split reads detected by TopHat were quantitated across introns “a” and “b”.

ORF57 is essential for the expression of KSHV lytic genes and productive KSHV replication by enhancing RNA stability, promoting RNA splicing and stimulating protein translation [50]. ORF57 is encoded from a spliced RNA transcript (57.2) encoding 15 N-terminal amino acids from a small 5′ exon that is spliced to a larger exon encoding the remainder of the 455 aa protein (Figure 10B and Figure S5). A second splice has been identified in iSLK.219 cells, which removes the C-terminal half of the protein (intron “b”) and encodes a novel 33 aa C-terminal domain (Figure 10B) [8]. Although the transcript (57.3) exhibiting this second splice event was associated with lytic reactivation after doxycycline-induced ORF50 expression [8], it was thought to be artefactual due to use of a cryptic splice site in the recombinant KSHV.219 virus [50]. Because this gene and its spliced transcripts have been linked to lytic replication, we analyzed the RNA reads mapping to the ORF57 UCDS, which targets the large second exon (Figure 10C). High levels of ORF57 reads were observed in the LEC-K1-3, BEC-K1-3 and TIME-K2, 3 infections, with transcript levels from 9518 to 10,528 TPM (Figure 10A,D). The ORF57 level in the TIME-K1 infection was 4 times higher (39,198 TPM), while the level in the BCBL-1 cells was ~3 times lower (2977 TPM). TopHat analysis identified a high level of split reads mapping across the first intron “a” (transcripts 57.2 and 57.3) demonstrating that the vast majority of the ORF57 transcripts were spliced at this point (Figure 10A,D). Splicing across intron “b” (transcript 57.3) was detected in all latent infections, except for the TIME-K1 cells (Figure 10A,D), representing 1%–3% of the spliced transcripts. The unspliced transcript (57.1) would encode the N-terminal 16 aa of ORF57 and 12 additional amino acids (ORF57A) encoded after the unused splice site (Figure 10B and Figure S5), and represents approximately 10% of the total ORF57 transcripts. Our data demonstrates that the ORF57 transcript (57.3) encoding ORF57B, containing the N-terminal half of ORF57 and an uncharacterized novel C-terminal domain (Figure S5), is expressed in latent infections in cell lines containing wild-type BCBL-1 virus, and therefore is not an artefact of the recombinant KSHV.219, as suggested previously [50].

### 2.11. Characterization of vIRF Alternate Splicing

**Figure 11:**
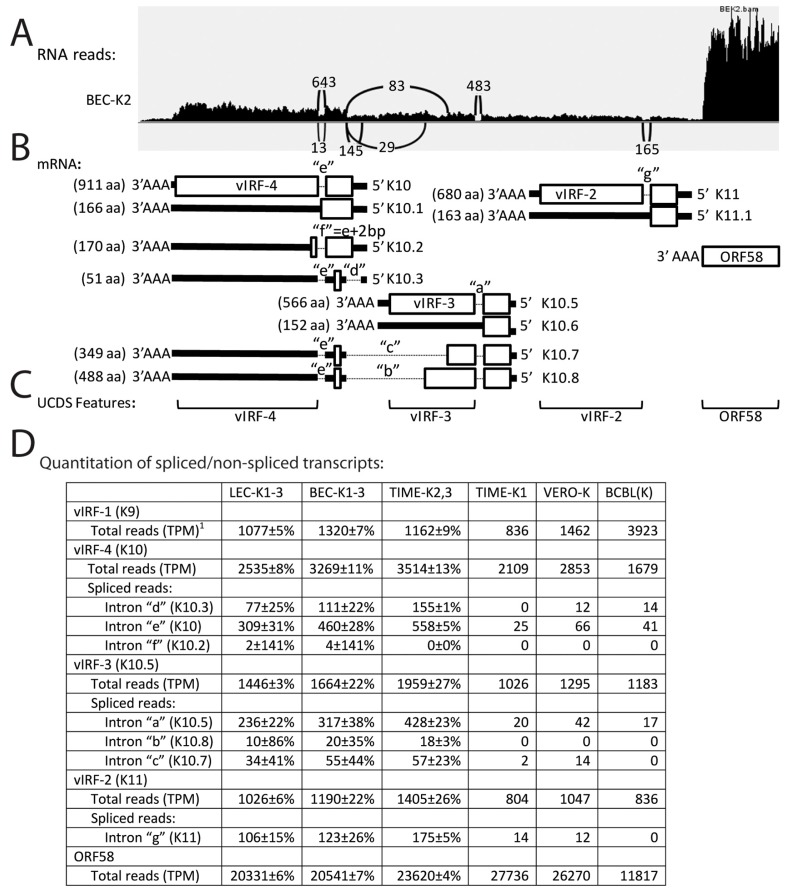
Alternate splicing of vIRF transcripts. RNA reads from the KSHV-infected cell cultures were mapped to the KSHV genomic region from vIRF-4 (K10) to ORF58. (**A**) An IGV Sashimi plot from TopHat analysis of split reads mapping across intron junctions of alternate vIRF transcripts; total RNA read depth is indicated in the vertical axis (representative data from the BEC-K2 infection). The position and number of split reads mapping across the intron junctions are shown; (**B**) the proposed transcription pattern for this region is indicated, with 5′ proximal exon coding regions boxed, adjacent 5′ and 3′ regions as solid lines, and intron regions as small dotted lines. The spliced K10 transcript encodes the 911 aa full length K10/vIRF-4; the unspliced K10.1 transcript encodes a 166 aa extended N-terminal domain of K10; the alternately spliced K10.2 transcript encodes a 170 aa isoform of the N-terminal domain of K10 with an alternate C-terminal peptide; the doubly spliced K10.3 transcript encodes a 51 aa peptide from an alternate frame of the K10 N-terminal domain; The spliced transcript K10.5 encodes the 566 aa full length K10.5/vIRF-3; the unspliced transcript K10.6 encodes the 152 aa N-terminal domain of K10.5; the triple spliced transcript K10.7 encodes a short truncation of K10.5 with the same C-terminal sequence encoded by K10.3 transcript; the triple spliced transcript K10.8 encodes a long truncation of K10.5 with the same C-terminal sequence encoded by K10.3 and K10.7. The spliced transcript K11 encodes the full length 680 aa K11/vIRF-2; the unspliced transcript K11.1 encodes the 388 aa N-terminal domain of K11 (see Figure S6 for encoded sequences); (**C**) The non-overlapping UCDS features for quantitating transcripts are shown. These features quantitate reads to the 3’ common exon and do not distinguish the different isoforms; (**D**) Total reads mapping to the UCDS features were quantitated and normalized (TPM) with the average and standard deviation for the replicate infections. Split reads detected by TopHat were quantitated across introns “a–g”.

Four viral homologs of interferon regulatory factors, vIRF-1/K9, vIRF-4/K10, vIRF-3/K10.5, and vIRF-2/K11 have been identified in KSHV (Figure 3) [51]. While the transcript of vIRF-1 is unspliced, vIRFs 4-2 are encoded by spliced transcripts lacking introns “e” (bp 88,443–88,543; NC_009333), “a” (bp 90,946–91,041), and “g” (bp 93,621–93,741), as depicted in Figure 11B, respectively. Previous microarray data suggested that a second vIRF4-related transcript (K10.3) expressed during KSHV latency in BC-3 lymphoma cells was alternatively spliced across both introns “d” (bp 88,899–89,134) and “e” (Figure 11B) [52]. The sequence within intron “d” encoded the “AUG” translation initiator for the spliced vIRF-4 translation product, and the loss of this sequence by alternate splicing left a possible 767 aa ORF encoding the C-terminal domain of vIRF-4 lacking the tryptophan-rich putative N-terminal DNA binding domain. It was proposed that this ORF could initiate from an alternate “CUG” initiation codon within the exon flanked by introns “d” and “e” [52]. 

To examine the spliced transcripts associated with KSHV latency, the total and spliced RNA-seq reads mapping to the genomic region of KSHV encoding the vIRFs were quantitated. Figure 11A shows a representative Sashimi plot of split RNA reads derived from spliced transcripts for the BEC-K2 infection. Total reads mapping to the vIRF transcripts were quantitated using UCDS features targeting the large downstream exons (Figure 11C). While the adjacent ORF59/58 bicistronic transcript showed high levels of expression (20–28,000 TPM) in the infected endothelial cells (Figure 11A,D), the vIRF transcript levels were much lower ranging from 804 TPM (vIRF-2/K11) to 3514 TPM (vIRF-4/K10) (Figure 11D). Analysis of the split RNA reads in the Sashimi plots revealed evidence for the typical spliced transcripts in the infected endothelial cells for vIRF-4/K10 lacking intron “e” (K10; median = 460 split reads, with 643 in BEC-K2 as shown in Figure 11A), vIRF-3/K10.5 lacking intron “a” (K10.5; median = 317 split reads, with 145 in BEC-K2) and vIRF-2/K11 lacking intron “g” (K11; median = 123, with 165 in BEC-K2). Unexpectedly, evidence for additional spliced transcripts within vIRF4 and vIRF3 was detected in the infected endothelial cells, indicating the existence of putative transcripts lacking intron “f” (bp 88,441–88,543) (K10.2; median = 4, with 13 in BEC-K2—see Figure 11A), transcripts lacking intron “b” (bp 88,899–90,146) (K10.8; median = 18, with 29 in BEC-K2), and transcripts lacking intron “c” (bp 88,441–90,506) (K10.7) median = 55, with 83 in BEC-K2) (Figure 11D). Transcripts lacking intron “f” are generated by splicing the exon encoding the N-terminal DNA binding domain of K10 to a variant splice acceptor site 2 bp downstream of the major acceptor site within the major vIRF-4 exon (Figure 11B). This transcript would encode a 170 aa protein, herein designated K10.2, which contains the N-terminal tryptophan-rich putative DNA binding domain with a remarkable C-terminal “RGGRGCXXC” motif encoded from an alternate reading frame of the major vIRF-4 exon (predicted sequence provided in Figure S6). The transcript lacking intron “b” is generated from an alternate splice donor site (bp 90,147; phase 0) within the major vIRF-3 exon to the splice acceptor site within the first exon of vIRF-4/K10 (bp 88,898) after intron “d”. This transcript is predicted to encode K10.8, which contains the N-terminal putative DNA binding domain of vIRF-3 (exon 1), the N-terminal half of the vIRF-3 major exon 2 and terminates with a cysteine-rich C-terminal 70 aa encoded by an alternate reading frame in the first exon of K10 (Figure 11B; predicted sequence provided in Figure S6). The transcript lacking intron “c” is generated from a different alternate splice donor site further upstream within the major vIRF-3 exon (bp 90,507; phase 0) to the same splice acceptor site within the first exon of vIRF-4/K10 (bp 88,898). This transcript would encode the same vIRF-3/C-terminal cysteine-rich domain fusion protein as the previous transcript with a further deletion of 120 aa from the vIRF-3 ORF in the major exon, herein designated K10.7 (predicted sequence provided in Figure S6). Interestingly, the C-terminal cysteine-rich domain contains a methionine encoded by the first AUG in the exon upstream of intron “e”. This AUG is the first possible AUG initiation codon in the double spliced transcript lacking introns “d” and “e” described above [52], suggesting that the cysteine-rich C-terminal domain, herein designated K10.3 (Figure 11B; sequence provided in Figure S6), could be the major translation product of this transcript, rather than the 767 aa ORF initiating from a possible CUG codon, proposed previously [52]. Although the existence of these transcripts is supported by substantial RNAseq data, verification is needed by other methods.

Surprisingly, 30%–50% of the reads mapping to the major vIRF-2, vIRF-3 and vIRF-4 UCDS features in the KSHV-infected endothelial cells were detected within the genomic region containing introns “g”, “a” and “e”, respectively (see Figure 11A and data not shown). This indicates that high levels of unspliced vIRF transcripts are generated in these cells. In the vIRF-2 and vIRF-3 loci, the unspliced transcripts would encode only the putative DNA binding domains in the first exon (K11.1: 163 aa; K10.6: 152 aa), as a translation stop signal is present immediately after the unused splice donor site (sequence provided in Figure S6). In the vIRF-4 locus, however, the unspliced transcript would encode K10.1, which contains the putative tryptophan-rich DNA binding domain and 10 additional amino acids with an “RGRGC” motif encoded after the unused splice donor site within the intron “e”. This C-terminal “RGRGC” motif in K10.6 is remarkably similar to the C-terminal “RGGRGCXXC” motif” in K10.2 that is encoded from the spliced transcript derived from the unusual alternate splice acceptor site following intron “f”. While “RGG” and “RG” motifs function as nucleic acid binding domains with affinity to primary and secondary nucleic acid structures, they are also implicated in protein-protein interactions and protein localization [53].

Analysis of the latent BCBL-1 infection revealed moderate levels of vIRF-1 (3923 TPM) that were ~4 times the level of vIRF-1 transcripts seen in the infected endothelial cells. In contrast, the BCBL-1 infection displayed only half the level of vIRF-2 and vIRF-3 transcripts detected in the endothelial cells (836 and 1183 TPM, respectively; Figure 11D). Overall fewer spliced vIRF transcripts were detected in the latent BCBL-1 cells with no evidence for spliced transcripts removing introns “b” or “c” in vIRF-3, “f” in vIRF-4, or “g” in vIRF-2 (Figure 11D). Similarly, the unusual TIME-K1 infection showed lower levels of expression of all vIRF transcripts, with no evidence for spliced transcripts removing introns “d”, “f”, or “b”. While a previous microarray study indicated that the spliced transcript lacking intron “e”, which encoded vIRF-4/K10, was not detected in latently infected BC-3 lymphoma cells, our study showed moderate levels of this transcript in all of the infected endothelial cells, except the TIME-K1, which was 10–20 fold lower.

### 2.12. Quantitation of Bicistronic Transcripts, ORFs 58–62

**Figure 12:**
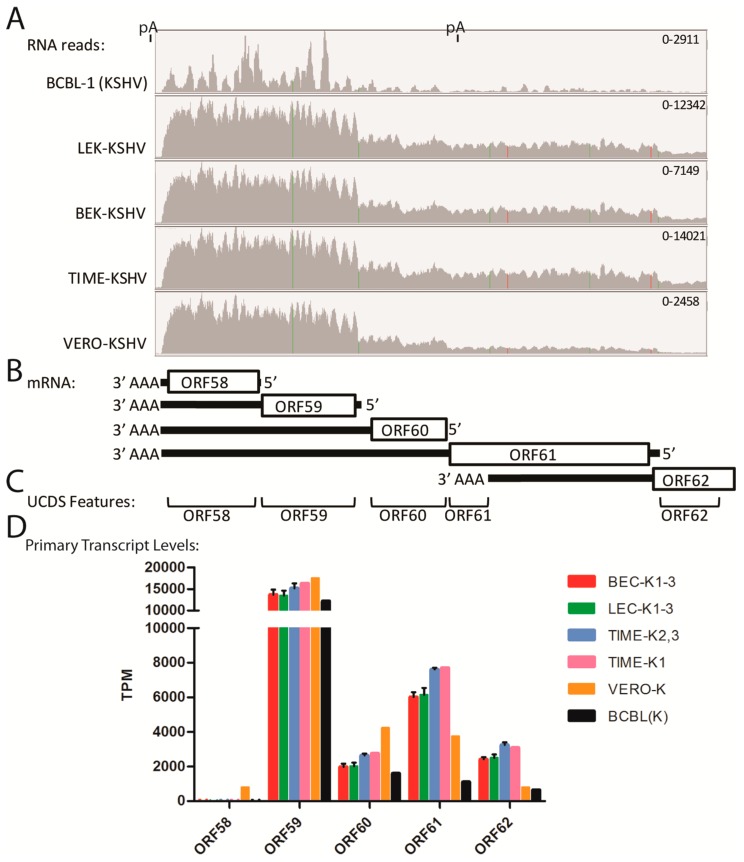
Quantitation of bicistronic transcripts (ORF58-ORF62). (**A**) RNA reads from the KSHV-infected cell cultures were mapped to the KSHV genomic region from ORF58 to ORF62, and representative data were visualized using IGV (see Figure 4 legend). The positions of known poly A (pA) sites are shown; (**B**) the proposed transcription pattern for this region is indicated; (**C**) The non-overlapping UCDS features for quantitating transcripts are shown; (**D**) Reads mapping to the primary transcripts for each ORF were quantitated using the UCDS features and normalized (TPM) with the average and standard deviation determined for the replicate infections (see Table S7 for supporting data).

The mapped reads in the ORF7-K3 region indicated that each ORF had its own adjacent promoter. In other regions, the mapped reads indicated that some transcripts were bicistronic with no evidence for a promoter driving additional expression of transcripts encoding only the distal ORF. High levels of reads in the five cell types mapped to ORF58 and ORF59 (Figure 12A). ORF58 is a homolog of EBV BMRF2, a multiple-pass transmembrane protein with unknown function, and ORF59, an early lytic protein, encodes a DNA polymerase processivity factor involved in DNA replication. Multiple transcripts encoding ORFs 62, 61, 60, 59 and 58 have been detected [54]. We developed non-overlapping UCDS features targeting these ORFs and quantitated gene expression (Figure 12B,C). All five cell lines showed high levels of reads mapping to the ORF58 UCDS (ranging from 11,817 TPM in BCBL-1 cells to 27,736 TPM in TIME-K1 cells) and to the ORF59 UCDS (ranging from 14,879 TPM in BCBL-1 cells to 28,672 TPM in the TIME-K1 cells) (Table S7). A gradient of total reads mapping to the UCDS features of ORF62, ORF61, ORF60 and ORF59 was observed in all cell lines (Table S7). This was compatible with the pattern of primary transcripts for each of these ORFs indicated in Figure 12B. The reads mapping to the primary transcripts, in which the designated ORF is 5′ proximal, were calculated (Table S7) and compared (Figure 12D and Figure S7). The transcript from the ORF59 promoter, which is bicistronic with the distal ORF58, was highly expressed across all 5 KSHV-infected cell types, including the TIME-K1 cells. While no evidence for a transcript from the ORF58 promoter was observed in the infected endothelial or BCBL-1 cells, a low level of the ORF58 primary transcript was detected in Vero cells (794 TPM) (Figure 12D; Table S7). Variable low level expression of the primary ORF60 and 62 transcripts was seen across the 5 cell types, with the lowest expression seen in the BCBL-1 cells. The primary ORF61 transcript was expressed at higher levels than ORFs 60 and 62 with the highest expression detected in the TIME cell infections (Figure 12D; Table S7).

### 2.13. Quantitation of Transcripts in the ORFK12-73 Latency Region

**Figure 13:**
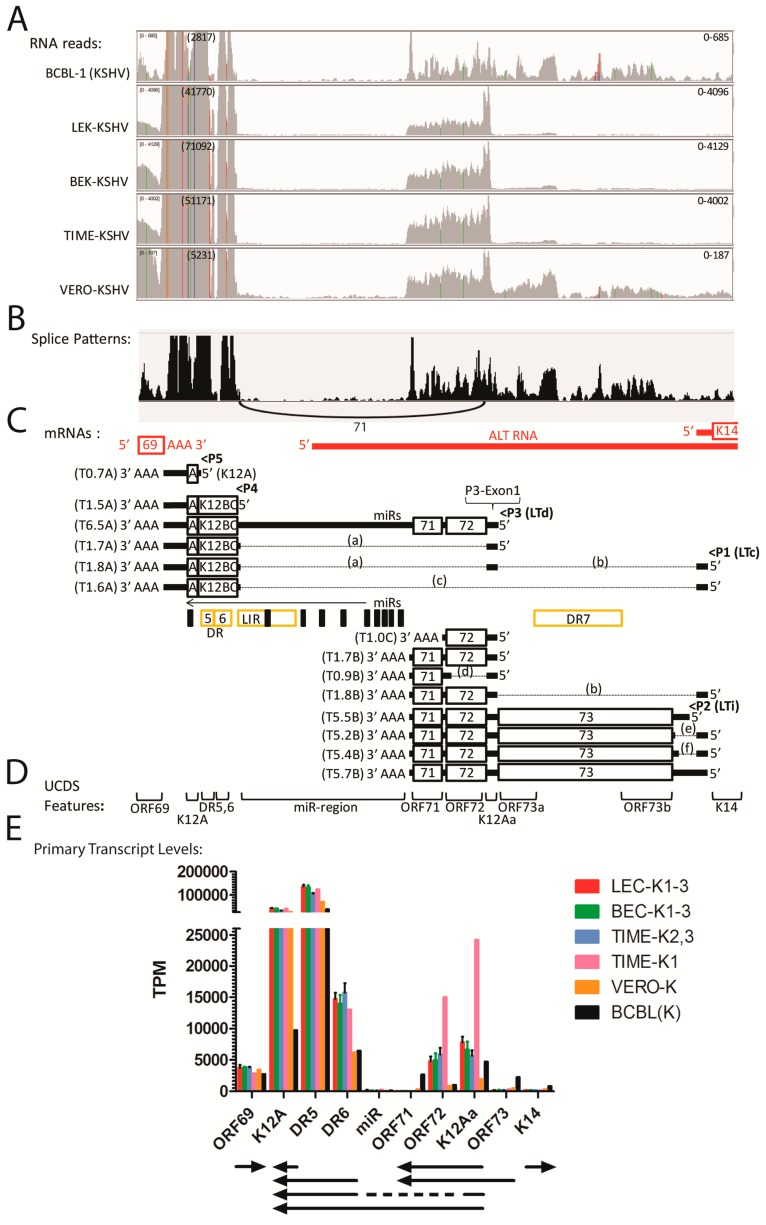
Quantitation of reads mapping to the latency region. (**A**) RNA reads from the KSHV-infected cell cultures were mapped to the KSHV genomic region from ORF69 to K14, and representative data were visualized using IGV (see Figure 4 legend). The range of the vertical axis (read count) was scaled to the ORF71/72 read depth indicated on the right and the depth of reads for the K12 region is indicated in parenthesis on the left; (**B**) Representative spliced transcript pattern (Sashimi plot) for latently infected BCBL-1 cells is shown. The number and position of reads split between the upstream and downstream exons are indicated; (**C**) The proposed transcription pattern for this region is shown (black = sense strand; red = antisense strand) [23,55,56,57,58,59,60,61]. The proposed non-coding antisense ALT RNA and the positions of the direct repeats DR5, 6 and 7, the indirect repeat LIR and the microRNA region (miRs) are indicated. Coding sequences are boxed, adjacent 5′ and 3′ regions are thick lines and introns (**a**–**f**) are indicated as thin dotted lines. The positions of the promoters for the latency transcripts are indicated: P1 (LTc; bp 128,815), P2 (LTi; bp 127,789), P3 (LTd; bp 124,055), P4 (~bp 119,035), and P5 (~bp 118,234) promoters and the known latency transcripts are graphically represented and are grouped according to their use of the polyA termination sites at bp 117,548 (Group A), bp 122,337 (Group B) and bp 123,010 (Group C), and the predicted sizes were determined from the GK18 NC_009333 reference sequence; (**D**) The non-overlapping UCDS features developed in this study for quantitating transcripts are shown. The miR UCDS feature identifies reads from the unspliced T6.5A transcript, which map to the genomic region encoding the micro RNAs. The miR UCDS feature in our study does not detect processed miRNAs, as these RNAs are not polyadenylated and thus are not sequenced in the RNA-seq library. The K12Aa UCDS feature identifies reads mapping to the short 5′ exon upstream of ORF72 (P3-Exon1), which is present in several spliced and unspliced transcripts in this region. The ORF73a and ORF73b UCDS features target the non-repetitive parts of the ORF73 transcript, and reads are combined for TPM normalization. The DR6 UCDS feature targets an extremely GC-rich repetitive region, which may be poorly amplified in the library preparation. E) Reads mapping only to the primary transcripts for each ORF were quantitated using the UCDS features and normalized (TPM) with the average and standard deviation determined for the replicate infections (see Table S8 for supporting data).

High levels of reads mapped to the DR5 repeats within the latency region flanking ORFK12 (Figure 13A), with transcript levels ranging from 38,833 TPM in BCBL-1 cells to 136,194 TPM in the LEC-K1-3 infections (Table S8). Reads mapping to the ORFK12A UCDS feature downstream detected transcript levels ranging from 9743 TPM in BCBL-1 cells to 43,101 TPM in the LEC-K1-3 infections (Table S8). These reads map to a region of the genome for which a variety of different spliced and unspliced transcripts have been identified previously (Figure 13C). The RNA reads mapping to the K12A, DR5 and DR6 UCDS features in all five cell types could be derived from the different transcripts encoding K12 Kaposin A, B and C (Figure 13C). Minimal reads mapped to the miR region UCDS feature indicating that the T6.5A unspliced transcript containing the Kaposin A/B/C region (Figure 13C) is minimally expressed (Table S8). The high level of reads mapping to K12A, DR5 and DR6 UCDS features are consistent with a high level of unspliced transcripts encoding either K12 Kaposin A (T0.7A) or variants of the DR5/6 repeats encoding Kaposin B/C (T1.5A) (Figure 13C). In addition, expression of the spliced T1.7A transcript encoding the Kaposin A/B/C region initiating from a small 5′ exon upstream of ORF72 (P3-Exon1) was supported both by the TopHat2 analysis, which detected RNA reads across the splice junction (Figure 13B,C—intron “a”; Table S9) and the high level of reads mapping uniquely to the K12Aa UCDS feature, which targets the P3-Exon1 (Figure 13A,E; Table S8). TopHat2 quantitation of reads spanning the splice junction for intron “a” revealed 71 split reads in infected BCBL cells (shown in Figure 13B) and 1179, 1094, 612 and 49 split reads in LEC-K1-3, BEC-K1-3, TIME-K2, 3 and VERO-K infections (Table S9), consistent with substantial levels of the T1.7A Kaposin A/B/C spliced transcript (Figure 13C, indicated with an asterix).

To avoid issues with the G/C-rich highly variable repeat region within the ORF73 coding sequence, discontiguous ORF73a and ORF73b UCDS features flanking the DR7 repeat region were utilized for read quantitation. Furthermore, reads were allowed to map only once to eliminate problems with repeat region reads mapping to multiple sites. Previous studies indicated that ORF73 transcripts are tricistronic with ORF72 and ORF71 with a poly(A) termination site downstream of ORF71 (Figure 13C; transcripts: T5.2B, T5.4B, T5.5B, T5.7B). Surprisingly, very few reads mapping to the ORF73 transcripts were detected in the latently infected endothelial cells 48 h pi (Figure 13A), with transcript levels ranging from 183 TPM (TIME-K2,3) to 311 TPM (TIME-K1) (Figure S8; Table S8). In contrast, up to 10 fold higher levels of ORF73 transcripts (2235 TPM) were detected in the infected BCBL-1 cells (Figure 13A,E; Table S8). There was little evidence for spliced transcripts initiating from the small 5’ exon upstream of ORF73, which encode either the Kaposin A/B/C region (transcripts: T1.6A, T1.8A), ORF71/72 (transcript: T1.8B) or ORF73 (transcripts: T5.2B, T5.4B) (Figure 13C, Table S9).

Moderate levels of reads mapped to the ORF71 and ORF72 UCDS features, indicating the presence of the unspliced T1.7B bicistronic ORF72/71 transcript (Figure 13A; indicated with a double asterix), with transcript levels ranging from 3286 TPM in BCBL(K) to 5830 TPM in TIME-K-2,3 (Figure 13D; Figure S8; Table S8). In the TIME-K1 infection, the level of the T1.7B transcript was 14,997 TPM, more than double that seen in the other TIME infections. Interestingly and consistently in the endothelial cells more than 1000 TPM mapped to ORF72 than mapped to ORF71 (Table S8), suggesting the presence of a novel transcript across ORF72 that would terminate before the ORF71 coding sequence (Figure 13C; transcript: T1.0C). Such an RNA species has been detected previously [55], and an “AATAAA” poly(A) signal is detected downstream of ORF71 (Table 2). In the BCBL-1 infection, in contrast, more reads mapped to ORF71 than ORF72, suggesting the low level presence of a monocistronic ORF71 transcript (T0.9B) that has been previously detected [56]. None of the other infected cell types showed evidence of a monocistronic ORF71 transcript (Figure 13D, Figure S8; Table S8).

### 2.14. Antisense Transcripts

Previously, transcripts were detected antisense to ORFs K1-11 (ALE; K1.3), K2 (K1.5), K4/K4.1 (K3.5), PAN/K7 (K7.3), K6 (T6.1/K4.5, T1.5/K4.7), 50 (T1.2, T3.0/K7.7), 58/59 (K11.5), and 71/72/73 (ALT) during lytic replication [44,48,62,63]. Mapping reads antisense to the UCDS features in the stranded libraries of our latent infections, we did not detect expression of K1.3, K1.5, K3.5, K7.3, K11.5 or ALT transcripts. However, moderate levels of transcripts were detected antisense to the DR1 repeat region, and ORFs K6, 29A, 29B, 50, 68, and 69 in the five latently infected cell lines (Table S6). The highest level of antisense transcript was detected across ORF29B in the infected LEC and BEC cells, with 1682 and 2465 TPM, respectively. This antisense transcript is downstream and in the same direction as the ORF28 transcript and could co-terminate with ORF28 at an AATAAA poly(A) signal within the ORF29B sequence (50,552–50,557 bp) (see Figure 3). In the infected LEC and BEC cells, the RNA-seq data indicated that the read levels of the ORF29BAS (antisense) and ORF28 transcripts are essentially the same (Table S6). This suggests that a single bicistronic transcript is produced from this region from the ORF28 promoter, containing ORF28 in a 5’ proximal position with a novel secondary 52 aa ORF 28A encoded in the distal end of the transcript antisense to ORF29B. Similar levels of reads (901 and 1083 TPM, respectively) mapped antisense to ORF29A in the infected LEC and BEC cells (Table S6), indicating the presence of an additional transcript upstream and in the same orientation as ORF34 (see Figure 3). Moderate levels of reads in all five cell types were also detected antisense to ORFs 68/69 and ORFK6 (Table S6). The transcripts antisense to ORFs 68/69 could be a continuation of the K12A transcripts that did not terminate earlier (see Figure 13). 

The ORFK6AS (T6.1) transcript detected previously initiates at the DR1 repeat region, encodes ORFK7 downstream and co-terminates with the PAN RNA (Figure 6B) [44]. Smaller transcripts, including T1.5 antisense to DR1, initiate at the same position but have different termination sites. Variable coding sequences have been proposed for these transcripts, including an origin of lytic replication associated protein (OLAP) [45]. Moderate levels of RNA reads from the stranded libraries mapped antisense to ORFK6 and ORFK5 in the infected LEC, BEC and BCBL cell types (Table S6), indicating the presence of the ORFK6AS transcript. This was further supported by the presence of reads mapping to the DR1, DR2 and OLAP UCDS features, which are in the same sense orientation as the ORFK6AS transcript (Figure 6; Table S6). Higher levels of reads mapping antisense to DR1/OLAP region were detected in BCBL cells, suggesting the presence of an additional poly(A)+ RNA transcript downstream of ORF K5 in the same orientation. 

### 2.15. Hierarchical Clustering of RNA Reads

**Figure 14:**
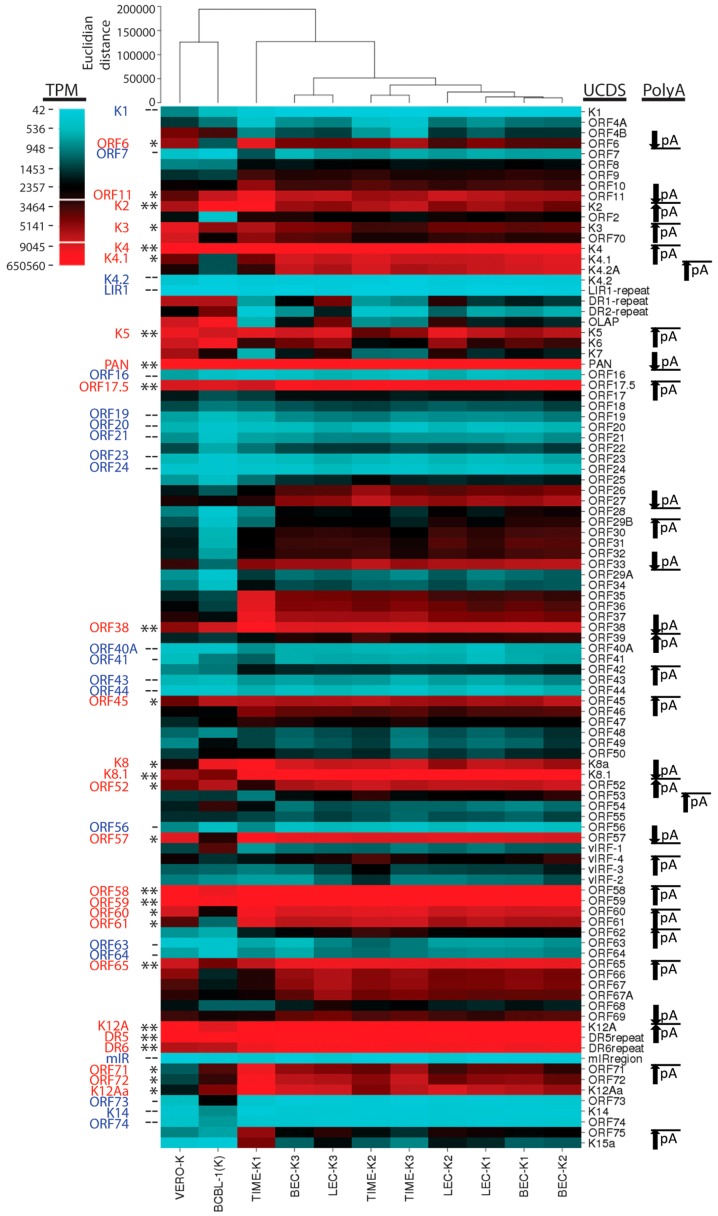
Hierarchical clustering of KSHV transcripts in cells undergoing latent KSHV infection. Hierarchical clustering was applied to viral gene expression data obtained by mapping RNA reads to UCDS gene features from triplicate independent latent KSHV infections of LEC, BEC and TIME cells, a single latent infection of Vero cells at 48 h post infection, and a single analysis of BCBL-1 cells carrying a latent KSHV infection. Total reads mapping to each KSHV UCDS feature were quantitated and normalized (TPM). The types of infected cells and replicate infections are indicated at the bottom of the analysis. UCDS features (described in Table 3) are indicated on the right hand side. Clustering of viral gene expression for the different infected cells is indicated (top horizontal axis) in the context of the order of genes within the KSHV genome (vertical axis). Expression levels are indicated (red (*) = strong; blue (-) = weak) and genes with consistent expression across the different cell types are shown on the left hand side (**/--: very consistent; */-: moderately consistent). The position of poly(A) transcription termination sites are indicated (pA) with arrows showing the extent of loci terminating at a common poly(A) transcription terminate site.

To visualize the RNA expression patterns of KSHV in the different latently infected cells, we analyzed the total RNA reads mapping to the UCDS features using a hierarchical clustering algorithm implemented in CIMminer [64]. This analysis would correspond to previous studies using RT-PCR, microarray or RNA-seq, since all reads mapping to a UCDS feature were quantitated, even if they were derived from overlapping polycistronic mRNAs. The initial analysis showed expression of the genes in their order within the KSHV genome (Figure 14). Low transcript levels were observed in a large number of viral genes (Figure 14, ORFs shaded blue). High expression levels of specific viral transcripts were detected across the whole KSHV genome in the endothelial and epithelial cells undergoing primary infection with KSHV, as well as in the BCBL-1 cell line with a long-term latent KSHV infection (Figure 14, ORFs labeled red). These peak read levels occurred proximal to poly(A) sites, as indicated (Figure 14) and a gradient of transcript levels was observed in genetic loci containing multiple transcripts initiating from different promoters and terminating in the same poly(A) site (Figure 14; arrows). Cluster analysis revealed that the KSHV gene expression was similar between the LEC, BEC, and TIME endothelial cells experimentally infected with KSHV, which grouped separately from the infected African green monkey kidney Vero cells and the pleural effusion lymphoma BCBL-1 cells carrying a long-term latent KSHV infection (Figure 14). 

**Figure 15:**
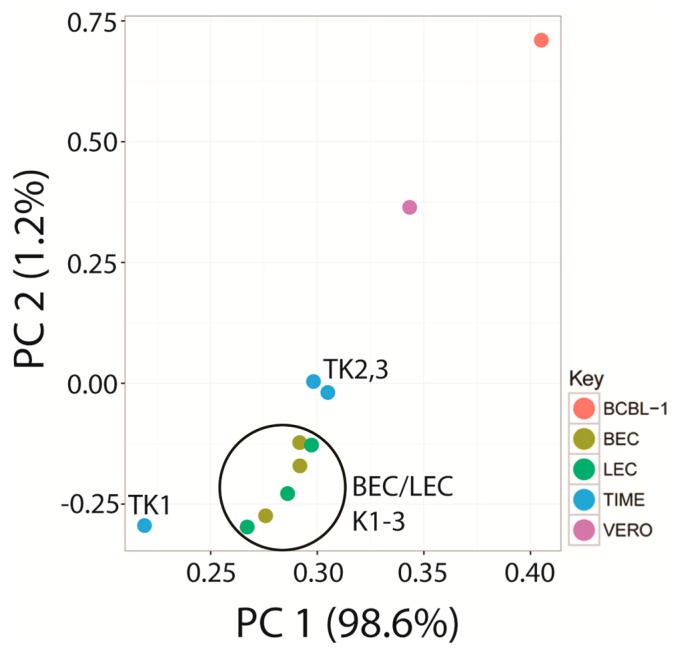
Principal component analysis (PCA) analysis of normalized KSHV gene expression in latently infected cells. Principal component analysis was performed on the viral gene expression data from the latently infected cells in Figure 14. The clustering of the triplicate infections of BEC and LEC cultures is shown.

Principal component analysis (PCA) was used to reduce the complexity of the expression data. Principal components 1 (PC1) and 2 (PC2) captured 98.6% and 1.2% of the variation in the gene expression data, respectively. Using this analysis, the replicate infections of the BEC and LEC endothelial cells clustered together, with overlap between the two cell types (Figure 15). While the pattern of KSHV expression was very similar in the replicate TIME-K2 and -K3 infections, the TIME-K1 infection was substantially different, clustering apart from the infected endothelial cells and the infected VERO and BCBL-1 cells (Figure 14 and Figure 15).

Although widespread gene expression was observed in all of the latently-infected cultures, there was evidence of differential expression of specific genes in the different cell types, as discussed above. The infected endothelial cell lines exhibited consistent expression of a number of genes associated with lytic replication, while the infected BCBL-1 cells and the Vero cells showed a more restricted pattern of gene expression, although the lytic PAN mRNA was still the major transcript. Obvious differences with the endothelial cells included higher levels of transcripts from immunomodulatory genes and lower levels of transcripts from genes involved in viral replication and virion structure, consistent with a more latent phenotype. 

To compare the RNA transcripts from the individual promoters associated with each KSHV ORF, we determined the transcript levels associated with the primary transcripts for each KSHV ORF, in which the designated ORF was 5′ proximal in the transcript, as described in Table 3. Reads mapping to upstream ORFs in known bicistronic and polycistronic transcripts were subtracted from the reads mapping to the designated ORF to obtain an estimate of the level of primary transcripts, as described in Table 3, Table S1, and Table S6–8. Unlike all previous studies that have not considered the effect of overlapping polycistronic transcripts on RNA read mapping, the analysis of primary transcripts described here, provides a quantitative estimation of the promoter function of each KSHV gene associated with a UCDS feature. A two-dimensional hierarchical clustering was performed on the log2 RNA read count from the replicate infections of the different LEC, BEC and TIME endothelial cells to visualize clustering of the primary transcript levels. 

**Figure 16:**
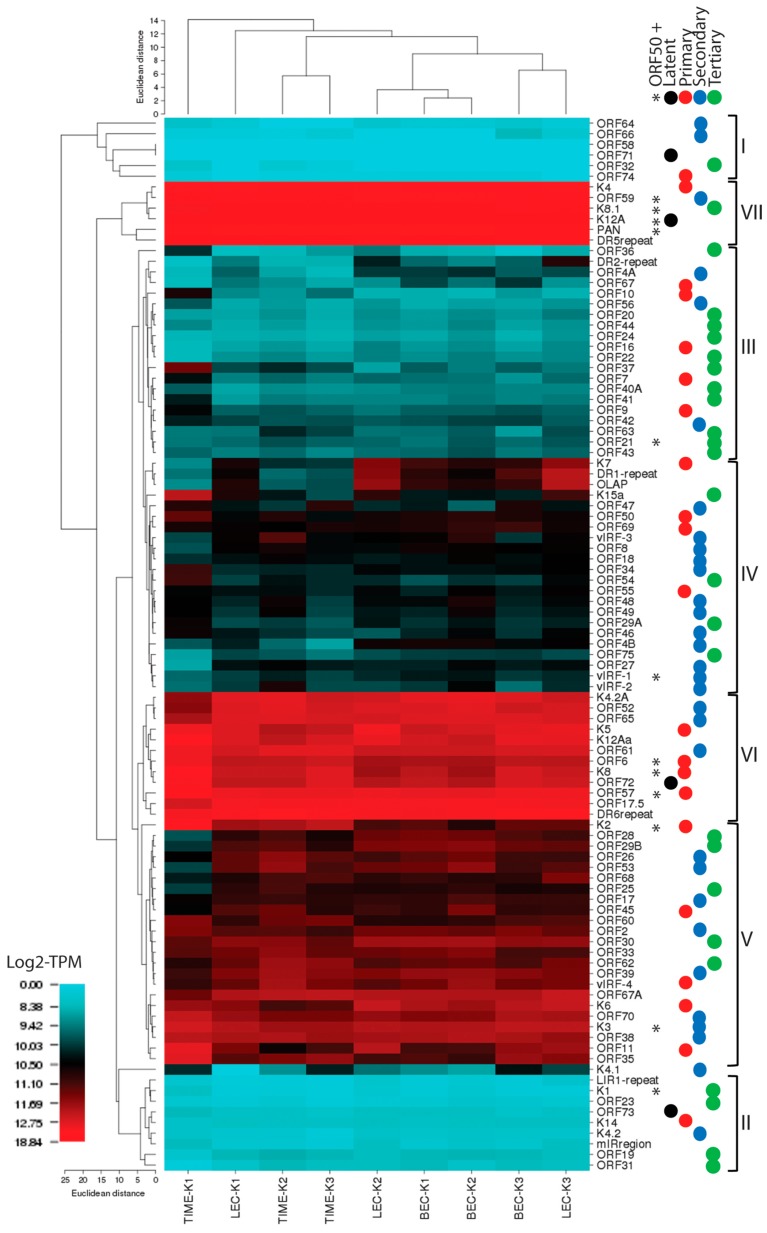
Hierarchical clustering of primary KSHV transcripts in endothelial cell lines undergoing latent infection. Two-dimensional hierarchical clustering was applied to viral gene expression data obtained by mapping RNA reads to UCDS gene features from triplicate independent latent KSHV infections of LEC, BEC and TIME cells. Reads mapping to UCDS features for primary transcripts of each KSHV ORF were quantitated, as described in Table 3, normalized (TPM) and converted to Log2 to visualize clustering. Clustering of the expression patterns for the different infected cells is indicated at the top and clustering of the viral gene expression is indicated at the left side with major clusters enumerated on the right side (Groups I-VII). UCDS features (described in Table 3) are shown on the right hand side. The classical pattern of gene expression obtained from latently-infected PEL cells (BC-3) before and after TPA induction of lytic replication, is shown on the right: Latency-associated genes (black), genes induced 0–10 h (primary; red), 10–24 h (secondary; blue) and 48–72 h (tertiary; green) after lytic induction [52]. Genes directly activated by KSHV ORF50 RTA are also indicated (*).

A set of primary transcripts with reads mapping to UCDS features for K4, K8.1, ORF59, K12A, PAN and DR5 in Group VII, and ORF6, K4.2A, K5, ORF17.5, K8, ORF52, ORF57, ORF61, ORF65, DR6, K12Aa, and ORF72 in Group VI were highly expressed in all of the latently infected endothelial cell cultures (Figure 16, shaded red). The most highly expressed transcript PAN, and transcripts for K4, K4.2A, and K5 are considered to be from lytic genes transcribed from the left end of the genome proximal to the long inverted repeat LIR1 (see Figure 3). The transcripts detected with UCDS features for K12A, DR5, DR6, ORF72 and K12Aa are transcribed from the latency locus proximal to the long inverted repeated LIR2 at the right end of the genome. These transcripts encode the Kaposin A/B/C complex, ORF72 and the KSHV microRNAs. The other highly expressed transcripts, including ORFs 6, 17.5, K8, K8.1, 52, 57, 59, and 61, are considered to be from lytic genes positioned across the length of the KSHV genome. The K8.1 transcript was detected with a UCDS feature targeting only the K8.1 specific 5′ exon used in all spliced forms of K8.1, and did not include reads mapping to the 3′ exon, which is also present in ORF50 and K8 polycistronic spliced transcripts from which the K8.1 specific exon is removed.

A second set of primary transcripts in Group V were moderately expressed in the different latent endothelial cell infections (Figure 16). While the TIME-K1 infection showed higher levels of transcripts for ORFs K2, K3, 11 and 35 in this group, lower levels of transcripts for ORFs 17, 25, 26, 28, 29B, 53, 60, and 68 were observed in the TIME-K1 infection compared to the other endothelial infections. The Group I genes showed the lowest levels of primary transcripts. These ORFs did not appear to be expressed from an adjacent promoter and were only detected as distal ORFs in bicistronic or polycistronic transcripts, as discussed for ORF58 above. Low levels of transcripts for genes in Group II were also observed in all the latently infected endothelial cells (Figure 16).

Previous studies have characterized the cascade of KSHV gene expression after experimental induction of lytic replication by exogenous factors, such as TPA, sodium butyrate and recombinant ORF50 RTA transactivator using microarray analysis. One such experiment analyzed viral gene expression in KSHV-infected BC-3 pleural effusion lymphoma cells after reactivation with TPA [52]. Cluster analysis revealed a temporal program of lytic gene expression, in which primary genes were expressed within 10 h after TPA treatment, secondary genes between 10 and 24 h and tertiary genes between 48 and 72 h. They concluded that the pattern of gene expression and assigned gene function was consistent with the known stages of the herpesvirus life cycle. We annotated the gene expression profile obtained after 48 h of natural latent infection of endothelial cells without chemical activation in our study (Figure 16) with the published temporal profile of gene expression obtained after TPA reactivation of the BC-3 cells. Unlike the gene expression detected after TPA reactivation in BC-3 cells, there was little correlation between the levels of gene expression in the infected endothelial cells and their designation as primary (red label), secondary (blue label) or tertiary (green label) genes (Figure 16). This was true for reads mapping to primary transcripts, in which the designated ORF is 5′ proximal on the RNA transcript (Figure 16), or for total reads mapping to the designated ORFs in all overlapping transcripts (Figure 14). A high percentage of ORFs known to be activated by the ORF50 transcriptional activator were represented in the highly expressed transcript Groups V-VII, however, other ORF50-activated ORFs, including ORF21, vIRF-1, and K1 were minimally expressed (Figure 16, asterix). Of the known latency-associated genes, only the primary transcripts for K12 (Kaposin) and ORF72 (vCYC) were highly expressed in the latently infected endothelial cells 48 h post infection (Figure 16, black label). Reads mapping to the K12Aa UCDS feature detect expression of the latency associated ORF72/71 bicistronic transcript as well as the initial exon of the spliced transcript containing the DR5 and DR6 repeat regions encoding the Kaposin B/C ORFs from which the intron encoding the miRNAs 1–9 is excised (see Figure 13). Although mature miRNAs are believed to be processed from the excised intron, the RNA-seq approach used in this study does not detect these RNA species since they lack the poly A tail used for preparation of the RNA-seq library. Only very low levels of ORF73 (LANA) transcripts were detected, and ORF71 (vFLIP) was only present as a distal ORF downstream of ORF72 in the bicistronic ORF72/71 transcript. Thus, a wide vary of KSHV genes were expressed in the latently infected endothelial cells 48 hpi, which were discordant with the previously characterized temporal cascade of lytic gene expression.

### 2.16. Hierarchical Clustering of the Matrix of Gene-Gene Expression Correlations

**Figure 17:**
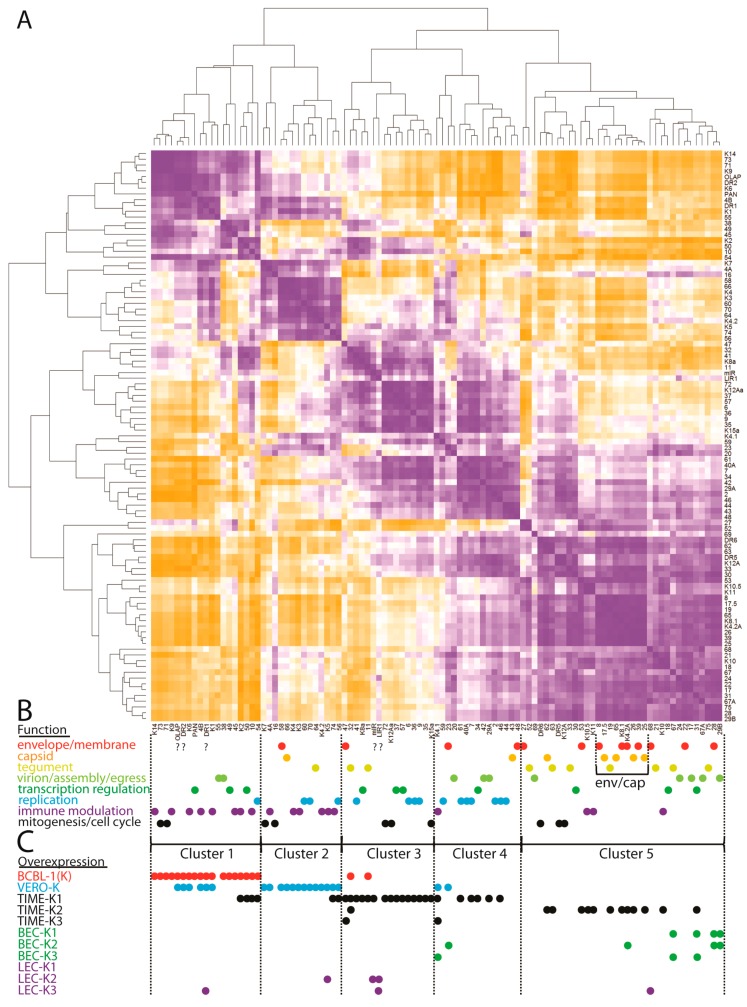
Hierarchical clustering of a gene-gene expression correlation matrix from different cell types latently infected with KSHV. (**A**) Pearson correlation coefficients (*R*) were calculated for pairs of KSHV genes using the counts of reads mapping to the gene-specific UCDS features, normalized to transcripts per million (TPM) for primary transcripts, in which reads from overlapping transcripts from polycistronic regions were removed, as described in the text and Table 3. This analysis compared the transcript expression from specific promoter/s associated with each ORF/gene feature. The negative correlation (*R* = −1.0) is shown in orange, the positive correlation (*R* = 1.0) in purple and the absence of correlation (*R* = 0) in white. The UCDS features used for mapping are indicated at the bottom and right side. The unsupervised hierarchical clustering is indicated on the top and left side, and the major clusters of genes (Clusters 1–5) are shown. (**B**) The functional/biological category associated with each ORF in panel A is indicated with a colored dot. (**C**) Genes in panel A with primary transcript levels that were 1.5 fold or greater than the mean of all eleven infected cell cultures and outside of the 95% confidence level are indicated with a dot color coded for each cell type. Significance of the clustering is discussed in the text.

To identify biologically relevant KSHV gene regulation modules, the Pearson correlation was determined between the primary transcript levels (TPM) of each KSHV gene pair for all of the latent KSHV infections in BEC, LEC, TIME, Vero and BCBL-1 cells. The comparison of primary transcripts allows the specific promoter activities for each KSHV gene to be compared in the different infected cultures. Gene expression in these cell cultures was a natural consequence of KSHV infection and was not experimentally induced by chemical treatments, such as sodium butyrate or phorbol esters, or by overexpression of recombinant proteins. Hierarchical gene clustering revealed groups of co-expressed genes that showed similar expression correlation profiles across the different latently infected cell types (Figure 17A), indicated by cohesive purple squares along the diagonal. These clusters of genes represent modules undergoing similar regulation of gene expression (Table S10). The orange areas showed clusters of genes that were negatively correlated. A functional enrichment analysis was performed by comparing the known functions or biological properties of the KSHV genes in each module (Figure 17B). Of note, a group of genes with highly correlated expression profiles (strong purple box) were identified in the Cluster 5 subgroup ENV/CAP (Figure 17B), whose members all had functional attributes related to late gene expression and KSHV virion structure, including envelope/membrane proteins (red dots—ORF8, glycoprotein B; K8.1 glycoprotein; K4.2A a novel membrane-associated protein; ORF39, glycoprotein M), capsid proteins (orange dots—ORF17.5, virion scaffold protein SCAF; ORF65, minor capsid protein SCIP; ORF26, triplex component TRI-2; ORF25, major capsid protein (MCP) and tegument protein (yellow dot—ORF19, capsid-associated tegument protein) (Table S11). The correlations within the envelope/capsid group of Cluster 5 (Figure 17B) were significant for both the envelope/membrane proteins (*p* = 0.017, odds ratio (OR) = 6.9) and capsid proteins (*p* = 0.001, OR = 21.2) (Table S12). 

The genes in the larger Cluster 5 expanded the number of virion structural genes with co-regulated expression, including additional envelope/membrane proteins (red dots—ORFs 53, 68, 22 and 28), capsid proteins (orange dot—ORF62), tegument proteins (yellow dots—ORFs 63, 33, 21, 67 and 75), as well as proteins involved in virion assembly and egress (light green dots—ORFs 24, 17, 67A and 29B) (Table S11). The correlations within the large Cluster 5 were significant for both the envelope/membrane proteins (*p* = 0.011, OR = 5.0), capsid proteins (*p* = 0.094, OR = 5.0), tegument proteins (*p* = 0.0318, OR = 4.9) and virion assembly/egress proteins (*p* = 0.1295, OR = 3.3) (Table S12). Examination of TATA-like promoter elements (see Table 2) for the genes in Cluster 5 revealed the shared presence of the non-consensus TATA motifs, TATTTAAA (*p* = 0.096, OR = 2.3) and its close homolog TATTAAA (*p* = 0.051, OR = 3.6), which have been implicated in temporal regulation of EBV and KSHV late gene expression [65,66] (Tables S13 and S14). 

Interestingly, the expression of the spliced homologs of the viral interferon regulatory factor, vIRF-4 (K10), vIRF-3 (K10.5) and vIRF-2 (K11) and the Kaposin complex (K12A, DR5 and DR6) also correlated with the late virion and membrane-associated genes in Cluster 5 (Figure 17B). vIRF-4 has been shown to cooperate with ORF50 in late gene expression [67], while Kaposin A is a membrane-associated protein through its two hydrophobic domains [68], suggesting functional similarities with the other Cluster 5 genes. In addition, the transcription/regulatory genes (dark green dots) in Cluster 5 included ORFs 18, 30 and 31, which are all involved in the regulation of late gene expression, and ORFs 18 and 30 have been specifically implicated in the regulation of the majority of genes in Cluster 5 [69,70]. Thus, the module of genes co-regulated in Cluster 5 is strongly associated with late gene expression and the assembly and structure of the infectious virion. 

Strong correlated expression of genes involved in DNA replication in Cluster 3 and 4 (*p* = 0.0001, OR = 9.6) and genes involved in immune modulation in Clusters 1 and 2 (*p* < 0.0001, OR = 10.3) were also observed (Figure 17B, Tables S10–S12). Significant correlations were detected with the promoter element “TATAA” upstream of the genes in Cluster 1 (*p* = 0.04141, OR = 3.9) and the promoter elements “TATTAAA” and “TATA” upstream of genes in Cluster 2 (*p* = 0.1902, OR = 2.6 and *p* = 0.0228, OR = 6.1, respectively) (Tables S13 and S14). A significant correlation was also detected with the TATA-like element “TAAAT” upstream of the genes in Cluster 4 (*p* = 0.0703, OR = 4.8). Surprisingly, the latency-associated genes involved in mitogenesis and cell cycle control (black dots) were scattered across the different gene clusters showing little evidence of co-regulated expression.

To further examine the significance of the gene regulatory modules observed in Figure 17, we identified KSHV genes that had unusually high expression levels in one of the eleven infected cultures. We calculated the confidence interval for the mean expression levels of the primary transcript for each KSHV gene across all eleven KSHV infected cultures, and identified genes whose expression level was outside the 95% confidence level and 1.5 fold or greater than the mean (Figure 17C, Table S15). This analysis clearly showed that the module of genes in Cluster 1, which showed co-regulated expression across all of the latently infected cell types, was significantly up-regulated in the long-term latent BCBL-1 infection compared to the other cell types undergoing primary infection (*p* < 0.0001, OR=347; Table S16). These co-regulated genes, including PAN, K2, K9 and ORF73, were associated with immune modulation, transcriptional regulation, cell cycle control and mitogenesis (Figure 17C). The module of genes in Cluster 2 was significantly up-regulated in the primary latent KSHV infection of the Vero epithelial cells (*p* < 0.0001, OR = 128), as was a subgroup of the Cluster 1 genes that were highly co-regulated across the different cell types (Figure 17A, purple box) and up-regulated in the BCBL-1 infection (Figure 17C, Table S16). The Cluster 2 genes were associated with a variety of functions, including immunomodulation and replication. Finally, the modules of co-regulated genes in Clusters 3 and 4 strongly associated with DNA replication were upregulated in the distinct TIME-K1 infection. Very few genes were overexpressed in the other infected endothelial cell cultures, with no consistency across the LEC, BEC and TIME cell types. Instead, these infected endothelial cells showed similar expression levels of genes in all of the clusters across the KSHV genome, as evidenced by the hierarchical clustering in the heat maps in Figure 14 and Figure 16 and the PCA analysis in Figure 15.

## 3. Discussion

We used RNA-seq to analyze the KSHV transcriptome in primary experimental latent infections of different endothelial and epithelial cell cultures and have compared this to long-term latent KSHV infection in a pleural effusion lymphoma cell line. Using this large amount of RNA data, we developed a comprehensive, high resolution map of the KSHV genome and its transcripts and identified unique UCDS gene features to map RNAseq reads and unambiguously quantitate KSHV mRNA transcripts. Our studies revealed that, overall, the pattern of KSHV transcription in the primary infected cell cultures 48 hpi was similar to the long term KSHV infection maintained in the BCBL-1 PEL cell line with high levels of the PAN (T1.1) and K12 (T0.7) mRNAs and extensive transcription across the complete KSHV genome. Very similar patterns of transcription were observed in the primary KSHV infections of blood-derived (BEC) and lymphatic (LEC) endothelial cell cultures. This contrasts with results observed previously in established LEC.219 and BEC.219 endothelial cell lines, in which long term infection with recombinant KSHV.219 was maintained under drug selection [38]. In this study, the selected BEC.219 cell line showed a restricted pattern of KSHV gene expression in the latency locus, considered to be representative of classical latency, while the LEC.219 cell line showed widespread gene expression across the entire KSHV genome, similar to that seen in the BCBL-1 cells and in the primary infections of LEC, BEC and VERO cells in our study. The widespread expression in the LEC.219 cells in the earlier study was not considered to be evidence of characteristic lytic replication, but instead was believed to be a novel transcriptional program that allows expression of traditional lytic genes without full-blown production of KSHV virions. Our studies show that this transcriptional pattern is not restricted to the lymphatic endothelial cell lineage. 

We observed minimal expression of correctly processed transcripts of ORF50 RTA, the replication transactivator required for induction of the lytic gene cascade, and minimal expression of genes involved in replication, including the ORFs 7, 9, 36, 40A, 41, 42, 46, 54, 56, 60, and 61. This correlates with previous studies, which have shown little evidence of productive replication in similar infected cultures without subsequent activation by chemicals, such as TPA or sodium butyrate, or addition of exogenous RTA. Furthermore, we observed little expression of antisense transcripts that have been detected during experimentally induced lytic replication [44,48,62,63]. However, the well-used marker of lytic replication, the ORF59 DNA polymerase processivity factor, was one of the most highly expressed genes in all of the latent infections. The historical use of ORF59 as a lytic marker was due its functional role with the viral DNA polymerase in replication, the early availability of a monoclonal antibody and its easily visualized nuclear location [71,72]. In our latent infections, we observed ORF59 protein expression in a small percentage of infected cells, similar to that seen previously [28,29]. In cells undergoing long-term latency, such as BCBL-1 cells and the drug-selected LEC.219 and BEC.219 cell lines, the presence of ORF59 protein has been attributed to spontaneous reactivation of viral latency. While de novo primary KSHV infection induces an early transient expression of RTA and a brief burst of RTA-induced lytic cycle gene expression, this disappears by 8 h post infection [30], and KSHV establishes the initial stages of latency by 24 h post infection [28,29]. Our RNAseq data shows that this transcriptional program of widespread KSHV gene expression is present in long-term latently infected BCBL-1 cell cultures as well as in BEC, LEC and VERO cells 48 h after primary infection. The presence of ORF59 protein and high levels of ORF59 transcripts suggests that this transcriptional program is due to spontaneous reactivation of viral latency in a small subset of infected cells and that ORF59 is a marker of this transcriptional program. Single-cell RNAseq analysis will be necessary to determine the relative contributions of reactivated and non-activated cells to this transcription pattern. Surprisingly, the expression of “lytic” gene transcripts did not correlate with expression of transcripts for ORF50 RTA, the master lytic switch regulator. Further time-course and single-cell studies are needed to determine the role of RTA in regulating the gene expression detected in these infections. 

Although the overall transcription patterns were similar between the long-term latent infection in BCBL-1 cells and the primary infection of BEC, LEC and VERO cells, unique qualitative and quantitative expression of specific genes was observed. We have limited our current analysis of the KSHV transcriptomes mainly to unspliced transcripts. However, due to the depth of reads in our RNA-seq data (5–100 fold more than previous studies), we detected a large number of previously unknown splice sites, which predict the existence of novel transcripts and encoded proteins throughout the KSHV genome. We specifically analyzed splicing of the ORF50 RTA and detected evidence of novel spliced transcripts containing the ORF50 coding sequence, which are derived from different upstream promoter regions, confirming and extending results from a recent study [49]. Complete characterization of these transcripts will be necessary to determine their structure and function. While evidence of low levels of correctly spliced characteristic ORF50 transcripts was observed in the infected endothelial cell cultures, this was lacking in the BCBL-1 cultures, as was evidence for the other novel spliced ORF50 transcripts. Our studies confirmed the expression of a spliced transcript removing the C-terminus of the ORF57 transactivator and detected evidence for novel spliced transcripts of the viral interferon regulatory factors, K10, K10.5 and K11 in the different infected cells.

To quantitate KSHV gene transcripts using RNA-seq read data, we developed a unique and powerful approach using constrained UCDS features to map viral reads to regions of the genome that are specific for a particular KSHV gene transcript. In regions with multiple polycistronic transcripts derived from different promoters that terminate at a common polyadenylation site, we developed an approach to determine the relative proportion of RNA reads mapping to each unique transcript. Since the KSHV genome, like other herpesvirus genomes, contains large regions of overlapping genes, our approach is the first to attempt to accurately quantitate the expression levels of the primary transcripts of all of the KSHV gene-specific promoters. We developed a new KSHV gene feature file (GFF) containing UCDS features specific for important regions of the KSHV genome, which are based on the transcription initiation and termination sites predicted from our RNA-seq data. These UCDS features were designed to be non-overlapping to eliminate ambiguous mapping results. Previous RNAseq, microarray and quantitative RT-PCR studies have based KSHV gene expression levels on the mapping of reads, probe hybridization or gene amplification to the known coding sequences for each gene, in most cases ignoring the contribution of overlapping or alternately spliced transcripts derived from different promoters. In these studies, therefore, expression levels detected for most KSHV genes must be viewed with caution. We have shown specific examples of these problematic areas and have detailed approaches to accurately quantitate expression levels of each KSHV promoter. As further information is gained regarding the structure and expression of KSHV gene transcripts, the features in our UCDS GFF can be easily modified to more accurately determine KSHV expression patterns. The characterization of these gene products and the development of UCDS features and approaches to quantitate these transcripts are ongoing.

A novel finding of our study was that peak levels of RNA transcripts across the KSHV genome were detected in ORFs that were proximal to poly(A) signals at the termination of mRNA transcripts. In most cases, a gradient of gene expression was detected in loci containing multiple ORFs whose transcripts all terminated after the same poly(A) signal, indicating that each ORF in polycistronic loci had its own promoter initiating a specific primary mRNA transcript. In many cases, the primary transcript of an ORF was bicistronic or polycistronic for additional secondary ORFs located in more distal positions. The 5′ proximal ORF is believed to be the major source of expressed protein in these transcripts, due to favorable ribosomal interactions and optimal translation initiation through cap-dependent mechanisms. Although protein expression of secondary ORFs can occur through internal ribosome entry sites (IRES) or ribosome reinitiation, the levels of protein expression of secondary ORFs are believed to be much less efficient than the primary ORF, as has been shown previously [56]. While our RNAseq data showed stepwise increases in reads mapping to ORFs flanking 3′ polyA transcription termination sites in polycistronic loci, we did not observe significant differences in read depth across individual ORFs that could be attributed to degradation of 5′ ends of mRNA transcripts.

Mapping RNA reads to UCDS features of ORFs in polycistronic loci allows primary transcripts for each ORF to be quantitated by subtracting the reads mapping to transcripts in which they are secondary, thus providing a more accurate assessment of gene expression from each unique promoter. In some cases, our RNA-seq data showed little evidence for primary monocistronic transcripts for specific ORFs, such as ORF71 and ORF58, even though there were high levels of RNA reads mapping to these ORFs in bicistronic transcripts. Using our UCDS approach, we were able to determine the expression level of the primary transcript for each gene from its associated promoter. Since the primary transcripts for each ORF are more likely to produce functional protein products a more accurate biological assessment of KSHV gene expression is obtained. This analysis will be enhanced as the KSHV transcriptome is more completely defined. However, correlations with protein expression are still needed to confirm these studies.

Currently, it is believed that the maintenance and spread of KSHV-associated tumors, such as Kaposi’s sarcoma, lymphoma and multicentric Castleman’s disease is dependent on spontaneous reactivation of KSHV latency in a small proportion of infected cells leading to the production of new infectious virions to maintain the infection and expression of genes involved in viral pathology. However, the pattern of gene expression observed in the latent KSHV infections in our study does not match the ordered expression of genes seen after experimental induction of lytic replication using phorbol esters, sodium butyrate or exogenous ORF50 RTA. In fact, modules of genes were detected, which were co-regulated in all of the infected cell types and upregulated in specific cell types. These results suggest that the expression of different KSHV genes is strongly influenced by cellular factors specific to the differentiated and/or proliferative state of the host cell. Quantitative analysis of the complex KSHV transcriptome using the UCDS features described in this study provides a more optimal approach to compare cell-specific viral expression patterns. We are currently using this approach to analyze the KSHV transcriptome in Kaposi’s sarcoma and other KSHV-associated diseases, as a basis for developing new therapies and diagnostic approaches.

It has been proposed that lytic genes, such as ORF74 (vGPCR), are expressed in unique patterns of KSHV latency associated with spontaneous reactivation, and play important biological roles in KS pathogenesis. However, in the latent infections in LEC, BEC, and TIME cells in our studies, essentially no expression of vGPCR was detected. Low levels of ORF73 LANA, K15 and ORF75 transcripts were detected in the latent infections of the LEC, BEC and TIME cells, but these were vastly overshadowed by higher levels of expression of numerous “lytic” gene transcripts. None of the latent infections in our study, including the BCBL-1 cell line, showed the very restricted latency pattern of gene expression observed in the tiled microarray analysis of the BEC.219 cells in the previous study [38]. It is possible that copy number of the KSHV episomes within the infected cells influenced the gene transcription pattern. Our latent infections were performed with sufficient infectious virus to infect 80%–90% of the cell cultures in order to optimize the levels of RNA detected by RNA-seq. This approach may have led to an increase in lytic gene expression, although expression of the ORF59 lytic marker by immunofluorescence was only observed in a small percentage of cells, typical of previous studies. Lytic gene expression has been observed previously in endothelial cell cultures undergoing high-efficiency primary infection [73]. It is clear that more extensive studies examining the KSHV transcripts following infection during time course experiments are now warranted given the data we have obtained at 48 h post infection, since the establishment of latency is believed to be an ongoing evolving process. 

In our study, transcripts of the PAN RNA, a major regulator of viral gene expression, showed the highest expression levels in all of the latently infected cell cultures, including BCBL-1 cells. Transcripts derived from the direct repeats DR5 and DR6 in the latency locus and the downstream ORFK12 showed the second highest level of expression. Although the majority of these transcripts appeared to be unspliced, produced from promoters upstream of K12 and DR5/DR6, moderate levels of spliced transcripts containing K12 and DR5/6 were produced from the ORF72 promoter [74]. These spliced transcripts consisted of a short 5′ region derived from an exon located upstream of the ORF72 coding sequence, which was spliced into an exon encoding the complex Kaposin A/B/C locus within DR5 and 6. The intron removed from this RNA transcript encodes the major microRNAs and is believed to be the source of processed microRNAs. Moderate levels of RNA reads in all latently infected cell types mapped to a bicistronic unspliced transcript also derived from the ORF72 promoter, which encoded the latency-associated ORF71 vFLIP and ORF72 viral cyclin homologs.

Although the majority of the KSHV transcripts in the biological replicates of the LEC, BEC and TIME cell infections showed low levels of variation (<10%), higher levels of variation were detected in some genes. While this was occasionally observed in transcripts with very low read depths, a number of genes in specific cell types showed high levels of variation in highly expressed transcripts in the replicate infections. These replicate infections were meticulously set up to have the same cell culture conditions and the same virion source and titer. However, there were unavoidable differences due to small variations in cell proliferation and confluency, as well as possible differences in infectivity of virus obtained from frozen stocks. Polybrene was used to enhance and equalize the level of infection in the endothelial cell cultures so that sufficient KSHV gene expression was induced for comparison purposes. The latent LEC and BEC infections (48 h post infection) yielded similar transcriptome patterns between the replicate infections, and very few differences were noted between the transcriptome patterns of the two infected cell types, in contrast with the KSHV-infected LEC.219 and BEC.219 cultures maintained under drug selection, described above. The 2nd and 3rd replicate KSHV infections of TIME cells also yielded very similar patterns of gene expression, which largely matched the patterns seen in the replicate LEC and BEC infections. However, the initial infection in TIME cells (TIME-K1) was significantly different with only 10% of the total KSHV-specific reads detected in the TIME-K2 and -K3 infections. Although the level of KSHV infection and activation was similar in the infected TIME cell cultures, as indicated by the ORF59 IFA staining, the pattern of gene expression was considerably different. Hierarchical cluster and principal component analyses showed a gene expression profile for the TIME-K1 infection that was quite distinct from the other endothelial cell infections, with high levels of transcripts encoding K2 (viral IL-6), K8 (K-bZIP) and members of the ORF35–38 locus, including ORF36 which phosphorylates K-bZIP [75] and ORF37, the host shut-off protein [76]. High levels of K2, K8 and ORF38 were also observed in the long-term latent infection in BCBL-1 cells, suggesting similarities in the latency states. The differences detected in the KSHV expression in the TIME cell infections may have resulted from differential uptake of KSHV during the initial infection.

Differences have been observed previously in other biological replicate experiments analyzing KSHV transcripts by RNA-seq. In the published KSHV 2.0 manuscript, Arias et al. used RNA-seq to analyze KSHV transcripts from latently infected iSLK.219 cells before and after reactivation by doxycycline induction of recombinant ORF50 RTA [8]. Although only indicated in the supplementary Table S3 of this manuscript, technical replicates of different time points before and after induction were analyzed. In this study, duplicate technical replicates of the latent uninduced iSLK.219 cell cultures yielded 9754 KSHV-specific reads in one case and 43,145 KSHV-specific reads in the replicate. In the first replicate, 595 PAN mRNA reads were detected, constituting 6.1% of the total KSHV reads. In the second replicate 34,473 PAN mRNA reads were detected, constituting 79.9% of the KSHV reads, a 60 fold increase. While this difference must have correlated with a large difference in the overall KSHV transcriptome pattern of these replicate latent infections, only data from the second replicate showing limited latency-associated transcription was presented. In the legend to the supplementary data, the authors indicated that the difference could be due to a possible cross contamination of the RNA library preparation or to an increased spontaneous lytic reactivation of the infected cells in the culture. In our study of latent infections, the total number of reads mapping to KSHV ranged from 6.0 × 10^5^ in the TIME-K1 infection to 8.8 × 10^6^ in the TIME-K3 infection. Whereas the KSHV read levels in the replicate BEC and LEC infections were at most two-fold different, the read level in the TIME-K1 infection was 6–10 fold less than the other LEC, BEC and TIME infections, similar to the difference in KSHV transcript levels in the iSLK.219 replicates of the KSHV 2.0 study. These results suggest that minor differences in the status of the target cell population or in the infection process can substantially affect the outcome of KSHV infection. 

Our updated transcript data and constrained UCDS features provide important advantages over other approaches to quantitate KSHV gene expression. First, this approach allows the transcript levels of specific genes from their associated promoter to be accurately determined without the contribution of reads derived from adjacent overlapping transcripts. For loci with multiple ORFs terminating at a common poly A site, our approach allows for quantitation of specific primary transcript levels for 5′ proximal ORFs. In loci with bicistronic transcripts, the quantitation with UCDS features can suggest the presence or absence of primary monocistronic transcripts. The UCDS approach avoids the problem of miscounting transcript levels for spliced genes that have exons shared with other transcripts and using stranded RNA-seq libraries can accurately distinguish reads mapping to sense and antisense-strand transcripts. The UCDS approach can predict the presence of previously unknown transcripts. Finally, although not used extensively yet, the UCDS approach can also be used to quantitate alternately spliced transcripts.

There are several caveats to the UCDS approach that should be considered. First, constraining the size of the gene feature to limit overlap decreases the number of reads mapping to a transcript causing potential problems with statistical variation in the case of low abundance transcripts. In our in vitro infection studies, this was not a major problem due to the abundance of the KSHV transcripts, 5 to 100 fold higher than other similar studies. Second, RNA-seq analysis is dependent on PCR amplification, whose efficiency can vary due to nucleotide content and sequence, and repeat and G/C-rich regions can be difficult to amplify. Limiting the size of a gene feature could artificially affect transcript quantitation if the transcript sequence was not homogenous across its length. To avoid such issues, we have eliminated high G/C-rich repeat regions from the UCDS features, such as LANA. Third, since RNA transcripts are purified by 3′ poly(A) selection to eliminate ribosomal RNA during the RNA-seq library construction, the 5′ ends of transcripts may be underrepresented if 5′ RNA degradation occurs. While this was seen in some previous RNA-seq analysis of biopsy tissues (unpublished data), the RNA samples from the in vitro infected tissue culture cells showed consistent read levels across longer RNA transcripts, with little evidence of overrepresentation of 3′ ends of transcripts. Finally, the correct analysis of the KSHV transcriptome depends on an accurate and comprehensive characterization of the viral transcriptome. We have examined the large amount of RNAseq read data and identified distinct changes in read depth that would correspond to the beginning or end of specific transcripts. By assigning the boundaries of these transcripts to adjacent TATA-like promoter elements or AATAAA-like polyadenylation signals, we have developed non-overlapping UCDS gene features to quantitate primary transcripts of every KSHV ORF. While the analysis of KSHV infected cells in our study has provided a more complete description of the transcripts derived from the KSHV genome, there are still many regions for which the transcriptional start and termination sites and splicing events are uncharacterized. Ideally, the extent of each transcript should be determined and verified experimentally. Our UCDS gene feature file could be easily updated to accommodate new transcript characteristics.

The cluster analysis of the gene correlation matrix from all of the latently infected cell cultures revealed modules of co-regulated gene expression. This co-regulation was a natural consequence of KSHV infection and was not induced by chemicals or exogenous recombinant protein expression, as is commonly done with KSHV. Furthermore, the co-regulated gene expression was based on RNAseq reads mapping to primary transcripts from the specific promoter elements we have identified for each KSHV gene. This contrasts with all previous expression studies that have not adequately considered the effects of overlapping transcripts on quantitation of gene expression from highly complex genomes, such as KSHV. While our expression data is based on our current knowledge of KSHV transcription, this study is the first to attempt to globally examine the promoter activity and resulting transcription levels of each KSHV gene during viral latency in different cell types.

## 4. Materials and Methods

### 4.1. Cell Lines

TIME cells are a tert-immortalized dermal microvascular endothelial cell line (McMahon laboratory) [77]. BEC cells are a neonatal dermal blood microvascular endothelial cell culture HMVECdBlNeo (CC2813; Lonza, Allendale, NJ, USA. LEC cells are a neonatal dermal lymphatic microvascular endothelial cell culture HMVEC-dLyNeo-Der (CC-2812; Lonza). Endothelial cells were maintained as monolayer cultures in EBM-2 medium (Lonza) supplemented with a bullet kit containing 5% fetal bovine serum, vascular endothelial growth factor, basic fibroblast growth factor, insulin-like growth factor 3, epidermal growth factor, and hydrocortisone (EGM-2 media). African green monkey Vero cells were from the American Type Tissue Culture. Vero cells were maintained in Dulbecco’s modified Eagle’s medium (DMEM) containing 10% fetal bovine serum (FBS). BCBL-1 cells latently infected with KSHV [78], were cultured in RPMI complete medium with 10% FBS, 100 U/mL penicillin, 100 µg/mL streptomycin, 1.0 mM HEPES, and 0.01% 2-mercaptoethanol at 37 °C.

### 4.2. Immunological Reagents

Rabbit anti-LANA antibody used to determine latent infections in the endothelial cells was a gift from Don Ganem. The ORF59 mouse monoclonal antibody, rat anti-LANA (clone LN53), goat anti-rat IgG-HRP used for the Vero cell infection were obtained from Advanced Biotechnologies, Eldersburg, MD, USA. Other reagents included TSA 488, TSA 594, TSA 647, TO-PRO 3, and SlowFade (Invitrogen, Carlsbad, CA, USA).

### 4.3. KSHV Infection

KSHV virions from culture medium of TPA-treated BCBL cells were concentrated approximately 200-fold by centrifugation onto an Opti-Prep cushion, as described previously [79]. The TIME, LEC, and BEC cells were infected in parallel with the same KSHV virion preparation on three different days (triplicate biological replicates). KSHV virions were titered previously and sufficient virus to infect >90% of the cells was used in the infection. The KSHV infections of endothelial cells were performed in serum-free EBM-2 supplemented with 8 μg/mL polybrene for 4 h, after which the medium was replaced with complete EGM-2. 30% more KSHV virion inoculum was used to infect the primary LEC and BEC cell cultures compared to the immortalized TIME cell lines to obtain similar infection rates. Infection rates were assessed by immunofluorescence for all experiments, using antibodies against the latency-associated nuclear antigen (LANA) and the lytic protein ORF59. KSHV infection of Vero cells was performed in a similar manner to the TIME cells, but in DMEM containing 10% FBS.

### 4.4. Immunofluorescence Assays

For analysis of KSHV expressed proteins, the Vero cultures were fixed with 4% paraformaldehyde (PF) in PHEM/sucrose (60 mM PIPES, 25 mM Hepes, 10 mM EGTA, 2 mM MgCl2, 4% sucrose) for 30 min at 37 °C. Free aldehydes were quenched with 50mM NH4Cl and the cells were permeabilized with 0.5% NP-40/0.2% Tween 20. Endogenous peroxidase activity was inhibited with 3% H_2_O_2_. The cells were incubated with 10% normal goat serum (NGS) followed by 10% milk containing 1% NGS (blotto/NGS) to block non-specific binding. To detect KSHV infected cells, the anti-LANA monoclonal antibody LN53 (1:500) was incubated with cells for 2.5 h. The cells were washed and bound antibody was detected with goat anti-rat IgG-HRP (1:100) followed by a 10 minute TSA amplification (Invitrogen) with TSA 488 or TSA 594 in colocalization experiments. The nuclei were stained with TO-PRO 3.

### 4.5. Confocal Microscopy

Confocal images were generated on an LSM 5 Pascal system (Zeiss, Thornwood, NY, USA) equipped with 40X 1.3 NA and 63X 1.4 NA objectives.

### 4.6. RNA-Sequencing

Total RNA was isolated using the NucleoSpin RNA kit (Machery-Nagle, Bethlehem, PA, USA). RNA was further concentrated and purified using the RNA Clean and Concentrator kit (Zymo Research, Irvine, CA, USA). RNA-seq libraries were constructed from 100 ng of total RNA using either the TruSeq RNA Sample Preparation Kit v2 or the TruSeq Stranded mRNA Library Kit (Illumina, San Diego, CA, USA).

### 4.7. Data Analysis

RNA reads mapping to the human genome (hg19) were removed using the Bowtie2 program (version 2.2.6) [80]. The remaining RNA reads were aligned to the KSHV reference sequence NC_009333 for the KSHV GK18 strain using TopHat2 (version 2.0.14) [2] in a local instance of Galaxy [81]. Mapped reads were visualized using Integrated Genome Viewer (IGV; version 2.3.75) [3]. For quantitation purposes, the reads from paired-end libraries (non-stranded) were analyzed as unpaired (single-end data) to allow each read of the pair to map unambiguously to a single gene feature. The reads from both strands of the stranded libraries (non-paired end) were either concatenated and analyzed together (librarytype = unstranded) for comparison to the non-stranded paired end libraries or were analyzed separately (librarytype = FR) to show strand specificity. TopHat2 was used to detect splicing events ab initio. The default presets were used except that the maximum intron length was decreased to 10,000 and the maximum number of alignments allowed was decreased from 20 to 1, to avoid overcounting reads to repetitive regions. HTSEQ-Count (version 0.6) was used to quantitate the reads mapping to our unique set of UCDS gene features within our novel revised gene feature file “KSHV NC_009333 UCDS ver 020116.GFF” (File S1). The “intersection (non-empty)” setting in HTSEQ was used to count all reads mapping completely or partially to a UCDS feature to maximize read count (featuretype = UCDS; IDattribute = gene). No reads were eliminated by ambiguity since the UCDS features were 50 bp apart, the length of a read. The read count was expressed as transcripts per million (TPM) by first normalizing the read count to reads per kilobase (RPK) by dividing the read counts by the length of the UCDS gene feature. The “per million” scaling factor was determined by summing all of the RPK values in a sample and dividing by 1,000,000. Each RPK value was then divided by the “per million” scaling factor to give TPM of mapped KSHV reads. Hierarchical clustering of expression levels was performed using the algorithm implemented in CIMminer [64], using TPM and log2 TPM to visualize gene clustering. Hierarchical clustering of the gene correlation matrix was performed by calculating the Pearson correlation between the primary transcript levels (TPM) associated with each pair of UCDS gene features, using a script in R to create and output the correlation matrix. The lists of genes in each cluster were analyzed using published information and classified into different functional groups. To identify overexpressed genes, the mean and 95% confidence level was determined from the primary transcript data for each UCDS feature across all eleven infected cell types. The genes showing transcript levels above the 95% confidence level and having 1.5 fold or higher transcript levels than the mean were selected and plotted against the correlation matrix. An R script was developed to determine the statistical significance of the correlation between functional and overexpression levels in gene clusters using Fisher’s exact test. A Shiny web application was developed for R-based principal component analysis, which is available at https://efg-ds.shinyapps.io/pcaApp/.

## Figures and Tables

**Table 1:** RNA sequencing (RNA-seq) libraries.

Infected Cell	IFA ^1^		RNA Library	Total Reads	KSHV	
	ORF73+	ORF59+			Reads	% Total
TIME-K1 ^2^	89%	3.4%	Paired-end	1.41 × 10^8^	6.25 × 10^8^	0.4%
			Stranded	4.06 × 10^7^	1.75 × 10^8^	0.4%
TIME-K2 ^2^	89%	1.0%	Paired-end	1.34 × 10^8^	8.19 × 10^6^	6.1%
TIME-K3 ^2^	94%	2.7%	Paired-end	1.22 × 10^8^	8.83 × 10^6^	7.2%
LEC-K1 ^3^	91%	8.6%	Paired-end	1.37 × 10^8^	7.25 × 10^6^	5.3%
			Stranded	4.17 × 10^7^	2.15 × 10^6^	5.1%
LEC-K2 ^3^	97%	6.0%	Paired-end	1.27 × 10^8^	4.61 × 10^6^	3.6%
LEC-K3 ^3^	84%	2.9%	Paired-end	1.37 × 10^8^	3.50 × 10^6^	2.6%
BEC-K1 ^4^	91%	12.3%	Paired-end	1.35 × 10^8^	6.29 × 10^6^	4.7%
			Stranded	4.09 × 10^7^	1.88 × 10^6^	4.6%
BEC-K2 ^4^	84%	<1%	Paired-end	1.47 × 10^8^	7.85 × 10^6^	5.4%
BEC-K3 ^4^	79%	2.6%	Paired-end	1.64 × 10^8^	5.51 × 10^6^	3.4%
Vero-K ^5^	95%	<2%	Paired-end	1.92 × 10^8^	1.21 × 10^6^	0.6%
BCBL1(K) ^6^	100%	<2%	Stranded	4.99 × 10^7^	1.01 × 10^6^	2.0%

^1^ IFA = immunofluorescence assay for latency marker ORF73 or lytic marker ORF59; ^2^ TIME-K = de novo Kaposi’s sarcoma-associated herpesvirus (KSHV)-infected Tert-immortalized (TIME) cells, 48 h post infection (hpi), infection rounds 1, 2 and 3; ^3^ LEC-K = de novo KSHV-infected lymphatic endothelial cells (LEC), 48 hpi, infection rounds 1, 2 and 3; ^4^ BEC-K = de novo KSHV-infected blood endothelial cells (BEC), 48 hpi, infection rounds 1, 2 and 3; ^5^ Vero-K = de novo KSHV-infected Vero cells, 48 hpi; ^6^ BCBL-1(K) = KSHV-infected pleural effusion lymphoma (PEL) cell line.

**Table 2:** KSHV TATA-like promoters and PolyA-signals.

Gene	TATA-Like Signal ^1^	Start (bp) ^2^	Stop (bp) ^2^	PolyA-Signal	Start (bp) ^2^	Stop (bp) ^2^	Strand	Verified ^3^
K1	TAATTTT	4	9	AUUAAA	1053	1058	+	?
ORF4	TTATTAAAA	1051	1059	AAUACA	2922	2927	+	mapped
ORF6	TATATAAA	3090	3097	AAUAAA	6974	6979	+	mapped
ORF7	Nd ^4^	-	-	AAUAAA	17,021	17,026	+	mapped
ORF8	TATTTAAA	8611	8618	AAUAAA	17,021	17,026	+	mapped
ORF9	TAAATTTA	11,230	11,237	AAUAAA	17,021	17,026	+	mapped
ORF10	TATAA	14,326	14,330	AAUAAA	17,021	17,026	+	mapped
ORF11	TATAT	15,568	15,572	AAUAAA	17,021	17,026	+	mapped
K2	TATTTTTAA	18,122	18,114	AAUAAA	17,171	17,166	-	mapped
ORF2	TATATAA	18,570	18,564	AAUAAA	17,171	17,166	-	mapped
K3	TTACAATAAA	19,793	19,784	AUUAAA	18,578	18,573	-	mapped
ORF70	TATA	21,116	21,113	AAUAAA	19,799	19,794	-	?
K4	TAATAAAA	21,912	21,906	AAUAAA	21,280	21,275	-	mapped
K4.1	TAATTTATAA	22,532	22,523	AAUAAA	21,912	21,907	-	?
K4.2A	TATTAAA	22,875	22,871	AAUAAA	21,912	21,907	-	?
K4.2	TAATTTAT	23,106	23,099	AAUAAA	21,912	21,907	-	?
OLAP (T1.5)	TATAA	24,137	24,141	AAUAAA	26,222	26,227	+	mapped
K5	TTATTT	27,017	27,022	AUUAAA	25,712	25,717	-	mapped
K6	TATAA	27,663	27,703	AAUAAA	27,067	27,072	-	mapped
K7	TATAT	28,639	28,643	AAUAAA	29,871	29,876	+	mapped
PAN	GATAAAA	28,788	28,794	AAUAAA	29,871	29,876	+	mapped
ORF16	TATATAAAA	30,176	30,184	AUUAAA	30,829	30,834	+	mapped
ORF17.5	TATTTAAA	31,838	31,846	AAUAAA	30,857	30,862	-	mapped
ORF17	TATTTAAA	32,606	32,613	AAUAAA	30,857	30,862	-	mapped
ORF18	TTATT	32,398	32,402	AUUAAA	33,530	33,535	+	mapped
ORF19	TATTTAA	35,050	35,056	AUUAAA	32,967	32,972	-	?
ORF20	CATTAAAT	35,711	35,718	AUUAAA	32,967	32,972	-	?
ORF21	AATAAT	35,296	35,291	AAUAAA	39,411	39,416	+	mapped
ORF22	TATTAAA	37,140	37,146	AAUAAA	39,411	39,416	+	mapped
ORF23	Nd	-	-	AAUAAA	39,345	39,350	-	mapped
ORF24	TATATA	43,042	43,047	AAUAAA	39,345	39,350	-	mapped
ORF25	TATAAAA	42,629	42,635	AAUAAA	48,841	48,846	+	mapped
ORF26	TATTAAA	46,975	46,981	AAUAAA	48,841	48,846	+	mapped
ORF27	TATATTTAAT	47,865	47,874	AAUAAA	48,841	48,846	+	mapped
ORF28	TATATATAA	48,999	49,006	AAUAAA	54,171	54,176	+	mapped
ORF29B	TTATTAAAAA	50,546	50,555	AAUAAA	49,461	49,466	-	mapped
ORF30	Nd	-	-	AAUAAA	54,171	54,176	+	mapped
ORF31	Nd	-	-	AAUAAA	54,171	54,176	+	mapped
ORF32	TATTTAAA	51,446	51,453	AAUAAA	54,171	54,176	+	mapped
ORF33	TATTAAA	52,805	52,811	AAUAAA	54,171	54,176	+	mapped
ORF29A	TTAAATT	54,880	54,886	AAUAAA	49,461	49,466	-	mapped
ORF34	AATAAAA	54,635	54,641	AUUAAA	58,933	58,938	+	mapped
ORF35	TATATAA	55,635	55,641	AUUAAA	58,933	58,938	+	mapped
ORF36	Nd	-	-	AUUAAA	58,933	58,938	+	mapped
ORF37	TATTTATATA	57,257	57,266	AUUAAA	58,933	58,938	+	mapped
ORF38	TATTAAA	58,439	58,974	AAUAAA	58,952	58,957	+	mapped
ORF39	TATTTAAAA	60,355	60,363	AAUAAA	59,000	60,363	-	mapped
ORF40A	TTAAATA	60,357	60,363	AAUAAA	62,639	62,644	+	mapped
ORF41	Nd	-	-	AAUAAA	62,639	62,644	+	mapped
ORF42	TATTTATAT	63,445	63,453	AAUAAA	62,534	62,639	-	mapped
ORF43	ATATTT	65,104	65,109	AAUAAA	62,534	62,639	-	mapped
ORF44	TATAAATA	63,446	63,453	AAUAAA	67,394	67,399	+	mapped
ORF45	TAAATTT	68,707	68,713	AAUAAA	67,444	67,449	-	mapped
ORF46	TTATAATAA	69,612	69,620	AAUAAA	67,444	67,449	-	mapped
ORF47	TATATAAA	70,088	70,096	AAUAAA	67,444	67,449	-	mapped
ORF48	TATTTA	71,551	71,556	AAUAAA	67,444	67,449	-	mapped
ORF49	ATTTATAA	72,856	72,863	AAUAAA	71,728	71,733	-	mapped
ORF50	TTTAAAAA	71,627	71,634	AAUAAA	76,813	76,818	+	mapped
ORF50AS(T1.2)	TATA	74,618	74,621	AAUAAA	73,621	73,616	-	mapped
ORF50AS(T3.0)	TATA	74,618	74,621	AAUAAA	71,728	71,733	-	mapped
K8	ATATA	74,592	74,596	AAUAAA	76,813	76,818	+	mapped
K8.1	TATTAAA	75,966	75,972	AAUAAA	76,813	76,818	+	mapped
ORF52	TATTTAAA	77,368	77,375	AAUAAA	76,823	76,828	-	mapped
ORF53	TATATAA	77,819	77,825	AAUAAA	77,377	77,382	-	?
ORF54	TTATATA	77,819	77,825	AAUAAA	78,787	78,792	+	mapped
ORF55	TATATTTAAT	79,832	79,841	AAUAAA	78,818	78,823	-	mapped
ORF56	TTTTATA	79,507	79,512	AAUAAA	83,078	83,713	+	mapped
ORF57	TATATAA	82,073	82,079	AAUAAA	83,078	83,713	+	mapped
vIRF-1	TATATA	85,410	85,415	AAUAAA	83,903	83,908	-	mapped
vIRF-4	TATAA	89,066	89,071	AAUAAA	86,125	86,130	-	mapped
vIRF-3	Nd	-	-	AAUAAA	89,487	89,492	-	mapped
vIRF_2	ATATATT	94,348	94,353	AUUAAA	91,881	91,886	-	mapped
ORF58	Nd	-	-	AAUAAA	94,584	94,589	-	mapped
ORF59	TATTTAA	96,923	96,929	AAUAAA	94,584	94,589	-	mapped
ORF60	Nd	-	-	AAUAAA	94,584	94,589	-	mapped
ORF61	TATATAAA	100,459	100,466	AAUAAA	94,584	94,589	-	mapped
ORF62	TATTTAAAAA	101,458	101,467	AAUAAA	98,399	98,404	-	mapped
ORF63	CATATTTA	101,165	101,172	AAUAAA	111,992	111,997	+	mapped
ORF64	TATTAAA	103,950	103,956	AAUAAA	111,992	111,997	+	mapped
ORF65	TATTAAA	112,652	112,658	AAUAAA	111,927	111,932	-	mapped
ORF66	TATATT	113,951	113,956	AAUAAA	111,927	111,932	-	mapped
ORF67	TATTTAAAAA	114,809	114,818	AAUAAA	111,927	111,932	-	mapped
ORF67A	AAATATTT	114,989	114,996	AAUAAA	111,927	111,932	-	mapped
ORF68	AAATATTT	114,989	114,996	AAUAAA	117,509	117,514	+	mapped
ORF69	TATAAAAA	116,287	116,294	AAUAAA	117,509	117,514	+	mapped
K12A	Nd	-	-	AAUAAA	117,548	117,553	-	mapped
miR-region	Nd	-	-	AAUAAA	117,548	117,553	-	mapped
DR5-repeat	Nd	-	-	AAUAAA	117,548	117,553	-	mapped
DR6-repeat	Nd	-	-	AAUAAA	117,548	117,553	-	mapped
Alt RNA	TATAT	120,457	120,461	AUUAAA	130,666	130,671	+	mapped
ORF71	Nd	-	-	AAUAAA	122,337	122,342	-	mapped
ORF72	ACATAAAA	124,055	124,062	AAUAAA	123,010	123,015	-	?
ORF73	TATAA	128,185	128,189	AAUAAA	123,010	123,015	-	?
K14	TATAA	127,966	127,970	AUUAAA	130,666	130,671	+	mapped
ORF74	TTTATTA	129,380	129,386	AUUAAA	130,666	130,671	+	mapped
ORF75	TATAGA	134,891	134,896	AAUAAA	130,655	130,660	-	mapped
K15	TATTTAT	137,077	137,083	AAUAAA	130,655	130,660	-	mapped

^1^ TATA-like promoter sequences were either identified previously (see NC_009333 accession record) or were identified upstream of putative transcription initiation sites from the RNAseq data analyzed in the current data sets; ^2^ Nucleotide positions determined from the KSHV genome Reference Sequence (NC_009333); ^3^ Poly A signals were detected in the KSHV genome and have been either predicted (?) or verified (mapped) [41,42]; Only the distal polyA signal is indicated, when multiple signals have been identified; ^4^ Nd = not determined or multiple.

**Table 3:** Unique coding sequence (UCDS) features.

UCDS	Description ^1^	Start (bp)	Stop (bp)	Size (bp)	Strand	Primary Transcript (Subtract) ^2^
K1	N-term CDS domain	105	747	643	+	
ORF4a	N-term CDS domain	1096	1985	890	+	
ORF4b	C-term CDS domain	2595	2764	170	+	
ORF6	Normal CDS	3179	6577	3399	+	
ORF7	N-term trunc/ORF6(3′NC), C-term trunc/ORF8(5′NC)	6980	8618	1639	+	
ORF8	N-term trunc/ORF7(CDS,3′NC)	8681	11,202	2522	+	ORF7 UCDS
ORF9	Normal CDS	11,329	14,367	3039	+	ORF8 UCDS
ORF10	C-term trunc/ORF11(5′NC,CDS)	14,485	15,522	1257	+	ORF9 UCDS
ORF11	N-term trunc/ORF10(CDS)	15,791	16,979	1223	+	ORF10 UCDS
K2	Normal CDS	17,227	17,841	615	-	ORF2 UCDS
ORF2	CDS C-term trunc/K2(5′NC)	18,114	18,519	406	-	
K3	Normal CDS	18,574	19,542	969	-	
ORF70	Normal CDS	20,023	21,036	1014	-	
K4	Normal CDS	21,480	21,764	285	-	
K4.1	Normal CDS	22,117	22,461	345	-	K4.2A UCDS
K4.2A	C-term domain K4.2	22,530	22,835	306	-	K4.2 UCDS
K4.2	N-term domain K4.2	22,885	23,078	549	-	
LIR1	5′ trunc/K4.2(CDS), 3′ trunc/DR1-repeat	23,128	24,180	1147	+	
DR1	Normal domain	24,230	24,808	579	+	
DR2	5′ trunc/DR1-repeat	24,858	25,045	223	+	
OLAP	N-term trunc/DR2-repeat	25,095	25,569	524	+	
K5	Normal CDS	25,865	26,635	771	-	
K6	Normal CDS	27,289	27,576	288	-	
K7	3′ trunc/PAN RNA	27,693	28,818	1126	+	
PAN	5′ trunc/K7(CDS)	28,868	29,895	1077	+	K7 UCDS
ORF16	Normal CDS	30,242	30,769	528	+	
ORF17.5	Normal CDS	30,920	31,786	867	-	ORF17 UCDS
ORF17	C-term trunc/ORF17.5 CDS	31,838	32,524	687	-	
ORF18	N-term trunc/ORF17(5′NC), C-term trunc/ORF19(CDS,3′NC)	32,605	32,967	363	+	
ORF19	N-term trunc/ORF20(CDS), C-term trunc/ORF18(3′NC)	33,535	34,709	1175	-	ORF20 UCDS
ORF20	N-term trunc/ORF19(CDS), C-term trunc/ORF21(CDS)	35,049	35,401	353	-	
ORF21	N-term trunc/ORF20(CDS), C-term trunc/ORF22(5′NC)	35,672	37,147	1476	+	
ORF22	C-term trunc/ORF23(CDS)	37,212	39,351	2193	+	ORF21 UCDS
ORF23	Normal CDS	39,401	40,615	1215	-	ORF24 UCDS
ORF24	N-term trunc/ORF25(3′NC), C-term trunc/ORF23(CDS)	40,665	42,636	2018	-	
ORF25	N-term trunc/ORF24(5′NC), C-term trunc/ORF26(5′NC)	43,041	46,982	3942	+	
ORF26	C-term trunc/ORF27(5′NC)	47,032	47,873	842	+	ORF25 UCDS
ORF27	Normal CDS	47,973	48,845	873	+	ORF26 UCDS
ORF28	Normal CDS	49,091	49,399	309	+	
ORF29B	Normal CDS	49,462	50,517	1056	-	
ORF30	Normal CDS	50,723	50,952	230	+	
ORF31	N-term trunc/ORF30(CDS), C-term trunc/ORF32(5′NC,CDS)	51,002	51,452	500	+	ORF30 UCDS
ORF32	N-term trunc/ORF31(CDS)	51,537	52,860	1324	+	ORF31 UCDS
ORF33	N-term trunc/ORF32(CDS)	52,910	53,865	1005	+	ORF32 UCDS
ORF29A	N-term trunc/ORF34(5′NC), C-term trunc/ORF33(3′NC)	54,176	54,665	490	-	
ORF34	C-term trunc/ORF35(5′NC,CDS)	54,774	55,688	984	+	
ORF35	C-term trunc/ORF36(CDS)	55,738	56,075	338	+	ORF34 UCDS
ORF36	N-term trunc/ORF35(CDS), C-term trunc/ORF37(5′NC,CDS)	56,190	57,269	1080	+	ORF35 UCDS
ORF37	N-term trunc/ORF36(CDS), C-term trunc/ORF38(5′NC,CDS)	57,409	58,396	988	+	ORF36 UCDS
ORF38	N-term trunc/ORF37(5′NC,CDS)	58,446	58,972	527	+	ORF37 UCDS
ORF39	Normal CDS	59,072	60,274	1203	-	
ORF40A	Normal CDS	60,407	61,780	1374	+	
ORF41	C-term trunc/ORF42(CDS)	61,926	62,543	618	+	Variable ^3^
ORF42	C-term trunc/ORF41(3'NC), N-term trunc/ORF43(CDS)	62,644	63,235	592	-	ORF43 UCDS
ORF43	N-term trunc/ORF44(CDS), C-term trunc/ORF42(5′NC,CDS)	63,444	64,991	1548	-	
ORF44	N-term trunc/ORF43(CDS)	65,052	67,357	2306	+	
ORF45	Normal CDS	67,452	68,675	1224	-	ORF46 UCDS
ORF46	C-term trunc/ORF45(5′NC)	68,963	69,503	541	-	ORF47 UCDS
ORF47	C-term trunc/ORF46(5′NC)	69,611	70,014	404	-	ORF48 UCDS
ORF48	Normal CDS	70,272	71,480	1209	-	
ORF49	Normal CDS	71,729	72,637	909	-	
ORF50	N-term trunc/ORF49(5′NC,CDS), C-term trunc/K8(5′NC)	72,856	74,688	1833	+	
K8a	N-term domain	74,949	75,422	474	+	ORF50 UCDS
K8.1	N-term domain	76,014	76,254	241	+	
ORF52	Normal CDS	76,901	77,296	396	-	
ORF53	Normal CDS	77,432	77,764	333	-	
ORF54	Normal CDS	77,835	78,722	888	+	
ORF55	N-term trunc/ORF56(5′NC)	78,864	79,513	650	-	
ORF56	N-term trunc/ORF55(5′NC)	79,831	82,066	2236	+	
ORF57	Normal CDS	82,326	83,644	1319	+	ORF56 UCDS
vIRF-1	Normal CDS	83,960	85,309	1350	-	
vIRF-4	C-terminal domain	86,174	88,442	2269	-	
vIRF-3	C-terminal domain	89,700	90,945	1246	-	
vIRF_2	C-terminal domain	92,066	93,620	1555	-	
ORF58	Normal CDS	94,577	95,650	1074	-	ORF59 UCDS
ORF59	N-term trunc/ORF60(3′NC), C-term trunc/ORF58(CDS)	95,700	96,658	1004	-	
ORF60	Normal CDS	96,976	97,893	918	-	ORF61 UCDS
ORF61	N-term trunc/ORF62(3′NC)	97,922	98,399	478	-	
ORF62	N-term trunc/ORF63(5′NC), C-term trunc/ORF61(5′NC)	100,458	101,171	714	-	
ORF63	N-term trunc/ORF62(5′NC)	101,495	104,100	2606	+	
ORF64	N-term trunc/ORF63(CDS), C-term trunc/ORF65(5′NC)	104,150	111,927	7822	+	ORF63 UCDS
ORF65	Normal CDS	112,037	112,549	513	-	ORF66 UCDS
ORF66	C-term trunc/ORF65(5′NC), N-term trunc/ORF67(CDS)	112,651	113,799	1149	-	ORF67 UCDS
ORF67	C-term trunc/ORF66(5′NC,CDS)	113,957	114,614	658	-	ORF67A UCDS
ORF67A	C-term trunc/ORF67(5′NC)	114,808	114,911	104	-	
ORF68	C-term trunc/ORF69(5′NC)	115,108	116,295	1188	+	
ORF69	Normal CDS	116,544	117,452	909	+	ORF68 UCDS
K12A	Normal CDS	118,025	118,207	183	-	
miR	Normal domain	119,048	122,159	3132	-	
DR5	5′ trunc/K12A(CDS)	118,257	118,490	262	+	
DR6	5′ trunc/DR5repeat	118,540	118,845	355	+	
ORF71	Normal CDS	122,393	122,959	567	-	ORF72 UCDS ^3^
ORF72	C-term trunc/ORF71(5′NC)	123,042	123,815	774	-	Variable ^3^
K12Aa	Small exon upstream of ORF72	123,843	124,054	212	-	
ORF73a	C-term domain	124,104	124,783	680	-	
ORF73b	N-term domain	126,456	127,446	991	-	
K14	Normal CDS	128,264	129,079	816	+	
ORF74	Normal CDS	129,520	130,548	1029	+	K14 UCDS
ORF75	Normal CDS	130,699	134,589	3891	-	K15a UCDS
K15a	Exon 8	134,824	135,287	464	-	

^1^ Normal coding sequence (CDS) feature from KSHV Reference sequence NC_009333—truncations (trunc) of the normal CDS are indicated to eliminate overlaps with adjacent transcripts, UCDS are spaced at least 50 bp apart to eliminate overlap of a 50 bp read; ^2^ To quantitate primary transcripts for the designated ORF in loci sharing a common polyadenylation transcription termination site, the transcript count from the adjacent upstream ORF is subtracted, as indicated; ^3^ Variable—different possible transcripts exist due to complex splicing patterns.

## Supplementary Materials

The following are available online at www.mdpi.com/2076-0817/6/1/11/s1, Figure S1: Comparison of the reads mapping to the KSHV genome in the RNA-seq libraries from different infected cell types, Figure S2: Comparison of reads mapping to overlapping transcripts between ORF6 and K3, Figure S3: Comparison of reads mapping to the transcripts of PAN and flanking ORFs, Figure S4: Comparison of reads mapping to the primary transcripts of ORFs 49–52, Figure S5: ORFs encoded by alternately spliced ORF57 transcripts, Figure S6: ORFs encoded by alternately spliced vIRF transcripts, Figure S7: Quantitation of reads mapping to the primary transcripts of ORFs 58–62, Figure S8: Quantitation of reads mapping to the primary transcripts of ORFs 69-K14, Table S1: Comparison of total and primary transcripts encoding ORFs 6-K3, Table S2: Comparison of total and primary transcripts encoding ORFs in the PAN region, Table S3: UCDS quantitation of overlapping ORF17/ORF17.5 primary transcripts, Table S4: Comparison of total and primary transcripts encoding ORFs 49–52, Table S5: Comparison of total and primary transcripts encoding ORFs 44–50, Table S6: Quantitation of sense and antisense transcripts, Table S7: Comparison of total and primary transcripts encoding ORFs 58–62, Table S8: Comparison of total and primary transcripts encoding ORFs in the latency region, Table S9: Quantitation of reads spanning splice junctions in the latency region, Table S10: Modules of co-regulated genes, Table S11: Functional classification of KSHV genes, Table S12: Significance of functional enrichment in gene regulatory modules, Table S13: KSHV promoter categories, Table S14: Significance of promoter element enrichment in gene regulatory modules, Table S15: Overexpression of KSHV genes in different infected cell cultures, Table S16: Significance of overexpression enrichment in gene regulatory modules, File S1: KSHV NC_009333 UCDS ver 020116.GFF.
