# Supplementary material for: Quantitative Analysis of the KSHV Transcriptome Following Primary Infection of Blood and Lymphatic Endothelial Cells
Source: Pathogens. 2017 Mar 19;6(1):11. doi: 10.3390/pathogens6010011 (PMC5371899; doi:10.3390/pathogens6010011)
Supplement: Supplementary file 1 [file pathogens-06-00011-s001.zip › Figure S1 - Total mapped reads.pdf]

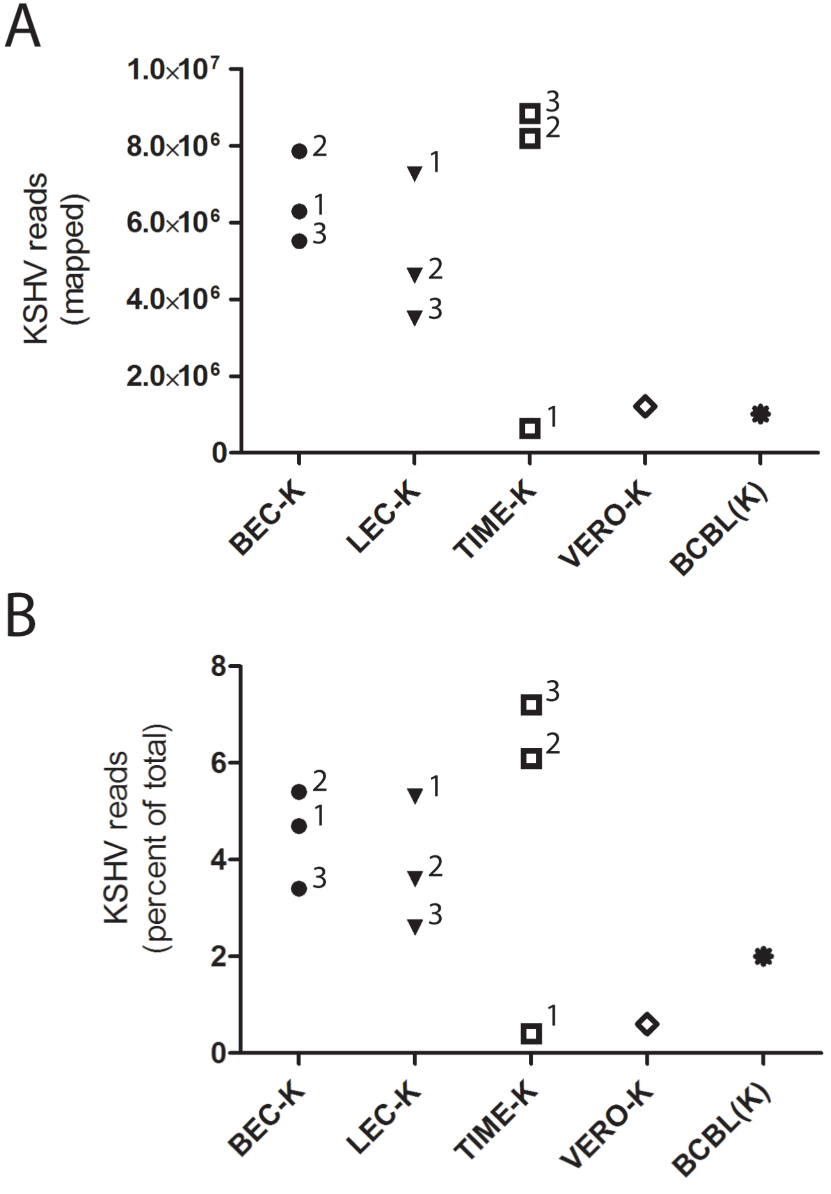

Figure S1. Comparison of the reads mapping to the KSHV genome in the RNA-seq libraries from different infected cell types. RNA reads from the RNA-seq libraries prepared from KSHV-infected BCBL-1 cells, three independent replicate primary KSHV infections of TIME (K1-3), LEC (K1-3) and BEC (K1-3) cell cultures and a single primary infection of VERO cells were mapped to the KSHV reference genome (NC_009333) (A) and compared to the number of total RNA reads (B). Notably, the number of KSHV mapped reads from the TIME-K1 infection was 10 fold less than the other TIME cell infections, indicating a significant difference in the infection outcome. The KSHV reads from the TIME-K1 infection were subsequently analyzed separate from the TIME-K2, -K3 infections to characterize this difference.
