# Supplementary material for: Quantitative Analysis of the KSHV Transcriptome Following Primary Infection of Blood and Lymphatic Endothelial Cells
Source: Pathogens. 2017 Mar 19;6(1):11. doi: 10.3390/pathogens6010011 (PMC5371899; doi:10.3390/pathogens6010011)
Supplement: Supplementary file 1 [file pathogens-06-00011-s001.zip › Figure S2 - ORF7-K3 mapped reads.pdf]

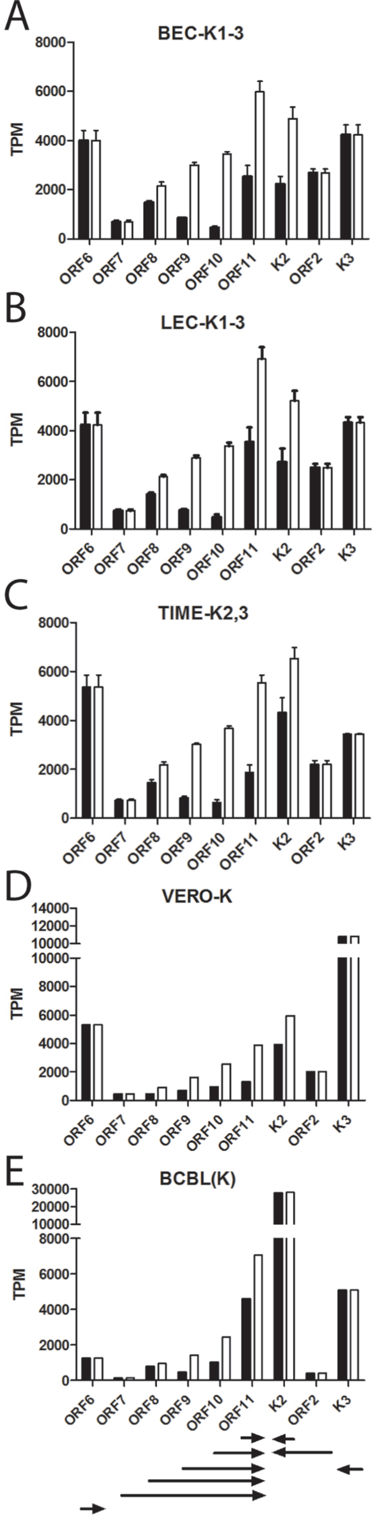

Figure S2. Comparison of reads mapping to overlapping transcripts between ORF6 and K3. The total number of reads mapping to the non-overlapping UCDS feature for each ORF was quantitated by HTSEQ and normalized (TPM) (open bars) with the average and standard deviation for the replicate infections. The number of reads mapping only to the primary transcript, in which the designated ORF is 5' proximal were quantitated by subtraction of the transcripts in which the designated ORF is distal in a polycistronic transcript (black bars; see Tables 3 and S1 for supporting data). A) BEC-K1-3; B) LEC-K1-3; C) TIME-K2,3; D) VERO-K; E) BCBL(K). The proposed pattern of transcription is indicated at the bottom.
