# Supplementary material for: Quantitative Analysis of the KSHV Transcriptome Following Primary Infection of Blood and Lymphatic Endothelial Cells
Source: Pathogens. 2017 Mar 19;6(1):11. doi: 10.3390/pathogens6010011 (PMC5371899; doi:10.3390/pathogens6010011)
Supplement: Supplementary file 1 [file pathogens-06-00011-s001.zip › Figure S3 - PAN region mapped reads.pdf]

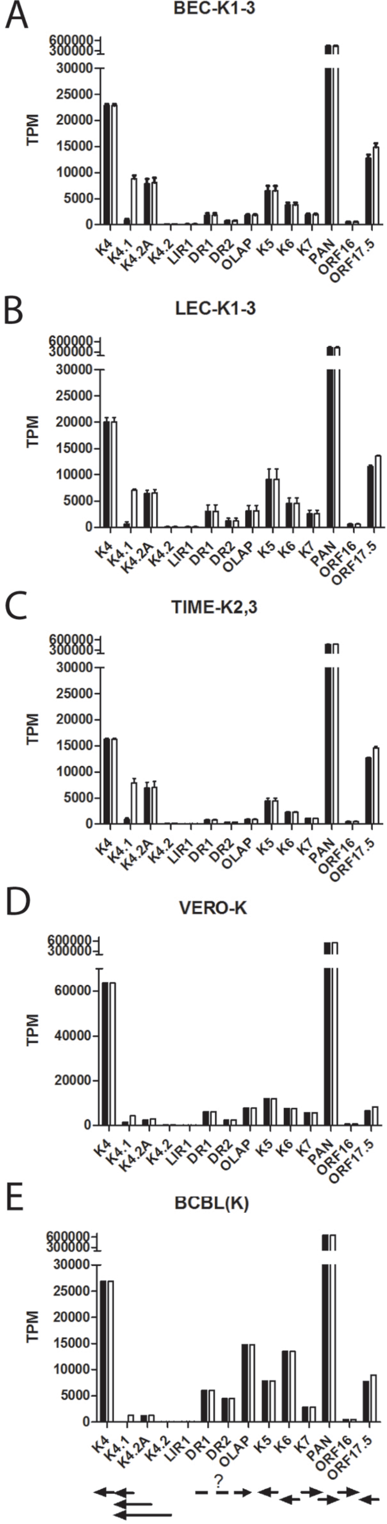

Figure S3. Comparison of reads mapping to the transcripts of PAN and flanking ORFs. The total number of reads mapping to the non-overlapping UCDS feature for each ORF was quantitated by HTSEQ and normalized (TPM) (open bars) with the average and standard deviation for the replicate infections. The number of reads mapping only to the primary transcript, in which the designated ORF is 5′ proximal were quantitated by subtraction of the transcripts in which the designated ORF is distal in a polycistronic transcript (black bars; see Table 3 and Table S2 for supporting data). A) BEC-K1-3; B) LEC-K1-3; C) TIME-K2,3; D) VERO-K; E) BCBL(K). The proposed pattern of transcription is indicated at the bottom.
