# Supplementary material for: Quantitative Analysis of the KSHV Transcriptome Following Primary Infection of Blood and Lymphatic Endothelial Cells
Source: Pathogens. 2017 Mar 19;6(1):11. doi: 10.3390/pathogens6010011 (PMC5371899; doi:10.3390/pathogens6010011)
Supplement: Supplementary file 1 [file pathogens-06-00011-s001.zip › Figure S5 - Alternately spliced ORF57 ORFs.pdf]

>ORF57A (28 aa)
MVQAMIDMDIMKGILEGKSSSTTDFSHF
>ORF57B (299 aa)
MVQAMIDMDIMKGILEDSVSSSEFDESRDDETDAPTLEDEQLSEPAEPPADERIRGTQSA
QGIPPPLGRIPKKSQGRSQLRSEIQFCSPLSRPRSPSPVNRYGKKIKFGTAGQNTRPPPE
KRPRRRRPRDRLQYGRTTRGGQCRAAPKRATRRPQVNCQRQDDDVRQGVSDAVKKLRLPAS
MIIDGESPRFDDSIIPRHHGACFNVFIPAPPSHVPEVFTDRDITALIRAGGKDDELINKK
ISAKKIDHLHRQMLSFVTSRHNQAYWDCQTPWQSVSRIPCNSRSLSLINKLPANYTQMK
>ORF57 (455 aa)
MVQAMIDMDIMKGILEDSVSSSEFDESRDDETDAPTLEDEQLSEPAEPPADERIRGTQSA
QGIPPPLGRIPKKSQGRSQLRSEIQFCSPLSRPRSPSPVNRYGKKIKFGTAGQNTRPPPE
KRPRRRRPRDRLQYGRTTRGGQCRAAPKRATRRPQVNCQRQDDDVRQGVSDAVKKLRLPAS
MIIDGESPRFDDSIIPRHHGACFNVFIPAPPSHVPEVFTDRDITALIRAGGKDDELINKK
ISAKKIDHLHRQMLSFVTSRHNQAYWVSCRRETAAAGGLQTLGAFVEEQMTWAQTVVRHG
GWFDEKDIDIILDTAIFVCNAFVTRFRLLHLSCVFDKQSELALIKQVAYLVAMGNRLVEA
CNLLGEVKLNFRGGLLLAFVLTIPGMQSRRSISARGQELFRTLLEYYRPGDVMGLLNVIV
MEHHSLCRNSECAAATRAAMGSAKFNKGLFFYPLS

Figure S5. ORFs encoded by alternately spliced ORF57 transcripts. The amino acid sequences of ORFs encoded by the putative alternately spliced transcripts 57.1 (ORF57A: 28 aa), 57.2 (ORF57: 299 aa) and 57.3 (ORF57B: 455 aa) are shown, as described in Fig 10. The distinct exon sequences are color coded.
