# Supplementary material for: Quantitative Analysis of the KSHV Transcriptome Following Primary Infection of Blood and Lymphatic Endothelial Cells
Source: Pathogens. 2017 Mar 19;6(1):11. doi: 10.3390/pathogens6010011 (PMC5371899; doi:10.3390/pathogens6010011)
Supplement: Supplementary file 1 [file pathogens-06-00011-s001.zip › Figure S6 - Alternately spliced vIRF ORFs.pdf]

>K10/vIRF-4 - transcript K10 (911 aa)

MPKAGGSEWATLWIIDALENNKFPYFSWFDRNNLLFAAPAPLPAGSDIPPGWYSVYHAFD
EECDRVYGPSPVVGQTVYGRFGRLLRGTRRAVVRNDLRYSDTFGGSYVVWQLVRTPFKNC
TYCYGAAYGPEKLQRFIQCLLSPPMQTTATRRSDTREQSYEEAGAAAPAPPKAPSGLRGR
PRKSNRYYNVGDITTEQKAACSVWIPVNEGASTSGMGSSGTRQVTQASSFTWRVPGDPPA
PSTLTGPSDPHSSGAGLPGTAPPKPQHETRLAGTVSGVSGVAQTPGDTGQLAPPMRDGSR
LPSTSPWIPACFPWGDLPVTGWWPQGASGLPEKVHPPTTGQFDPLSPRWTYTGIPSSQLN
PAAPSWIPPHAQAGTFVGEFSQGAPLAPQGLLPQSGQCASAWLPRRETGAEGACGASTEG
RAPQGAASERVYPFEPQPPSAPAPGYAKPSCYNWSPLAEPPATRPIRAPVWHPPVGHAVV
PEVRTPLWIPWSSGGAPNQGLSHTQGGASATPSAGAPPTPEVAERQEPSSSGIPYVCQGD
NMATGYRRVTTSSGALEVEIIDLTGDSDTPSTTVASTPLPVSGPRVFQPTVLYSAPEPAV
NPEVSHLPTELERRECVCPGSGERPRVPLVSTYAGDRYAVGGYGPEQSLVPPPLGLPLTL
SNLQGEDICTWEEGLGNILSELQEEPSSSTRQATDRRRPRSRSPHGRRTPVSHSGPEKPP
SKMFFDPPDSQRVSFVVEIFVYGNLRGTLRREGDAGEAMLCSWPVGDTLGHLCQSFVPEL
LRIPRLTVPSPEQMEILNRVFEGLGHGFPIFCSMSGIYSRNATQVEGWWFGNPNSRYERI
LRSFSPRVPQQLFNTARYLATTAAIPQTPLSVNPVTCGTVFFGASPASTENFQNVPLTVK
IFIGSIWDSLH

>K10A – transcript K10.1 (166 aa)

MPKAGGSEWATLWIIDALENNKFPYFSWFDRNNLLFAAPAPLPAGSDIPPGWYSVYHAFD
EECDRVYGPSPVVGQTVYGRFGRLLRGTRRAVVRNDLRYSDTFGGSYVVWQLVRTPFKNC
TYCYGAAYGPEKLQRFIQCLLSPPMQTTATRRSDTRyvtrgrgcev

>K10B – transcript K10.2 (170 aa)

MPKAGGSEWATLWIIDALENNKFPYFSWFDRNNLLFAAPAPLPAGSDIPPGWYSVYHAFD
EECDRVYGPSPVVGQTVYGRFGRLLRGTRRAVVRNDLRYSDTFGGSYVVWQLVRTPFKNC
TYCYGAAYGPEKLQRFIQCLLSPPMQTTATRRSDTRtklrggrgcstcsp

>K10C – transcript K10.3 (51 aa)

MHLMRSVIESTDHRLSWDRRCMDVLGDCCVEPGGPSCGTIYGTATHLVVAT

>K10.5/vIRF-3 – transcript K10.5 (566 aa)

MAGRRLTWISEFIVGALDSDKYPLVKWLDRSTGTFLAPAARNDVIPLDSLQFFIDFKREC
LSKGLHPRDLLGSPITAFGKICTTSRRLRRLPGEEYEVVQGINCRRWRLLCAEVKECWWC
VHARTHLHSGSSLWEILYQHSVRLEKHRRRPRPFVGENSDSSEEDHPAFCDVPVTQTGAE
SEDSGDEGPSTRHSASGVQPVDDANADSPGSGDEGPSTRHSDSQPPPADETTVHTDNVED
DLTLLDKESACALMYHVGQEMDMLMRAMCDEDLFDLLGIPEDVIATSQPGGDTDASGVVT
EGSIAASAVGAGVEDVYLAGALEAQNVAGEYVLEISDEEVDDGAGLPPASRRRPVVGEFL
WDDGPRRHERPTTRRIRHRKLRSAYYRVARPPVMITDRLGVEVFYFGRPAMSLEVERKVF
ILCSQNPLADISHSCLHSRKGLRVLLPKPDDNNTGPGDVNLLAAVLRSFASGLVIVSLRS
GIYVKNLCKSTVLYHGNNPPKKFGVICGLSSRAVLDVFNVAQYRIQGHEHIKKTTVFIGG
DPTSAEQFDMVPLVIKLRLRSVTCDD

>K10.5A – transcript K10.6 (152 aa)

MAGRRLTWISEFIVGALDSDKYPLVKWLDRSTGTFLAPAARNDVIPLDSLQFFIDFKREC
LSKGLHPRDLLGSPITAFGKICTTSRRLRRLPGEEYEVVQGINCRRWRLLCAEVKECWWC
VHARTHLHSGSSLWEILYQHSVRLEKHRRRPR

>K10.5B – transcript K10.7 (488 aa)

MAGRRLTWISEFIVGALDSDKYPLVKWLDRSTGTFLAPAARNDVIPLDSLQFFIDFKREC
LSKGLHPRDLLGSPITAFGKICTTSRRLRRLPGEEYEVVQGINCRRWRLLCAEVKECWWC
VHARTHLHSGSSLWEILYQHSVRLEKHRRRPRPFVGENSDSSEEDHPAFCDVPVTQTGAE
SEDSGDEGPSTRHSASGVQPVDDANADSPGSGDEGPSTRHSDSQPPPADETTVHTDNVED
DLTLLDKESACALMYHVGQEMDMLMRAMCDEDLFDLLGIPEDVIATSQPGGDTDASGVVT
EGSIAASAVGAGVEDVYLAGALEAQNVAGEYVLEISDEEVDDGAGLPPASRRRPVVGEFL
WDDGPRRHERPTTRRIRHRKLRSAYYRVARPPVMITDRLGVEVFYFGRPAMSLEVERKPP
RPCLPAAISRLAGIRCTMHLMRSVIESTDHRLSWDRRCMDVLGDCCVEPGGPSCGTIYGT
ATHLVVAT

>K10.5C – transcript K10.8 (368 aa)

MAGRRLTWISEFIVGALDSDKYPLVKWLDRSTGTFLAPAARNDVIPLDSLQFFIDFKREC

LSKGLHPRDLLGSPITAFGKICTTSRRLRRLPGEEYEVVQGINCRRWRLLCAEVKECWWC
VHARTHLHSGSSLWEILYQHSVRLEKHRRRPRPFVGENSDSSEEDHPAFCDVPVTQTGAE
SEDSGDEGPSTRHSASGVQPVDDANADSPGSGDEGPSTRHSDSQPPPADETTVHTDNVED
DLTLLDKESACALMYHVGQEMDMLMRAMCDEDLFDLLGIPEDVIATSQPGGDTDASGV**PP**
RPCLPAAISRLAGIRCTMHLMRSVIESTDHRLSWDRRCMDVLGDCCVEPGGPSCGTIYGT
ATHLVVAT

>K11/vIRF-2 – transcript K11 (680 aa)

MPRYTESEWLTDFIIDALDSGRFWGVGWLDEQKRIFTVPGRNRRERMPEGFDDFYEAFLE
ERRRHGLPEIPETETGLGCFGRLLRTANRARQERPFTIYKGKMKLNRWIMTPRPYKGCEG
CLVYLTQEPAMKNMLKALFGIYPHDDKHREKALRRSLRKKAQREAARKQAAAVATPTTSS
AAEVSSRSQSEDTESSDSENELWVGAQGFVGRDMHSLFFEEPEPSGFGSSGQSSSLLAPD
SPRPSTSQVQGPLHVHTPTDLCLPTGGLPSPVIFPHETQGLLAPPAGQSQTPFSPEGPVP
SHVSGLDDCLPMVDHIEGCLLDLLSDVGQELPDLGDLGELLCETASPQGPMQSEGGEEGS
TESVSVLPATHPLESSAPGASVMGSGQELPDLGDLSELLCETASPQGPMQSEGGEEGSTE
SVSVLPATHPLESSAPGASVMGSSFQASDNVDDFIDCIPPLCRDDRDVEDQEKADQTFYW
YGSDMRPKVLTATQSVAAYLSKKQAIYKVGDKLVPLVVEVYYFGEKVKTHFDLTGGIVIC
SQVPEASPEHICQTVPPYKCLLPRTAHCSVDANRTLEQTLDRFSMGVVAIGTNMGIFLKG
LLEYPAYFVGNASRRRIGKCRPLSHRHEIQQAFDVERHNREPEGSRYASLFLGRRPSPEY
DWDHYPVILHIYLAPFYHRD

>K11A – transcript K11.1(163 aa)

MPRYTESEWLTDFIIDALDSGRFWGVGWLDEQKRIFTVPGRNRRERMPEGFDDFYEAFLE
ERRRHGLPEIPETETGLGCFGRLLRTANRARQERPFTIYKGKMKLNRWIMTPRPYKGCEG
CLVYLTQEPAMKNMLKALFGIYPHDDKHREKALRRSLRKKAQR

Figure S6. ORFs encoded by alternately spliced vIRF transcripts. The amino acid sequences of ORFs encoded by the putative alternately spliced transcripts of K10/vIRF-4: K10 (K10/vIRF-4: 911 aa), K10.1 (K10A: 166 aa), K10.2 (K10B: 170 aa), and K10.3 (K10C: 51 aa); K10.5/vIRF-3: K10.5 (K10.5/vIRF-3: 566 aa), K10.6 (K10.5A: 152 aa), K10.7 (K10.5B: 488 aa), K10.8 (K10.5C: 368 aa); and K11/vIRF-2: K11 (K11/vIRF-2: 680 aa), K11.1 (K11A: 163 aa) are shown, as described in Fig 11. The distinct exon sequences are color coded.
