# Supplementary material for: Quantitative Analysis of the KSHV Transcriptome Following Primary Infection of Blood and Lymphatic Endothelial Cells
Source: Pathogens. 2017 Mar 19;6(1):11. doi: 10.3390/pathogens6010011 (PMC5371899; doi:10.3390/pathogens6010011)
Supplement: Supplementary file 1 [file pathogens-06-00011-s001.zip › Figure S8 Latency region mapped reads.pdf]

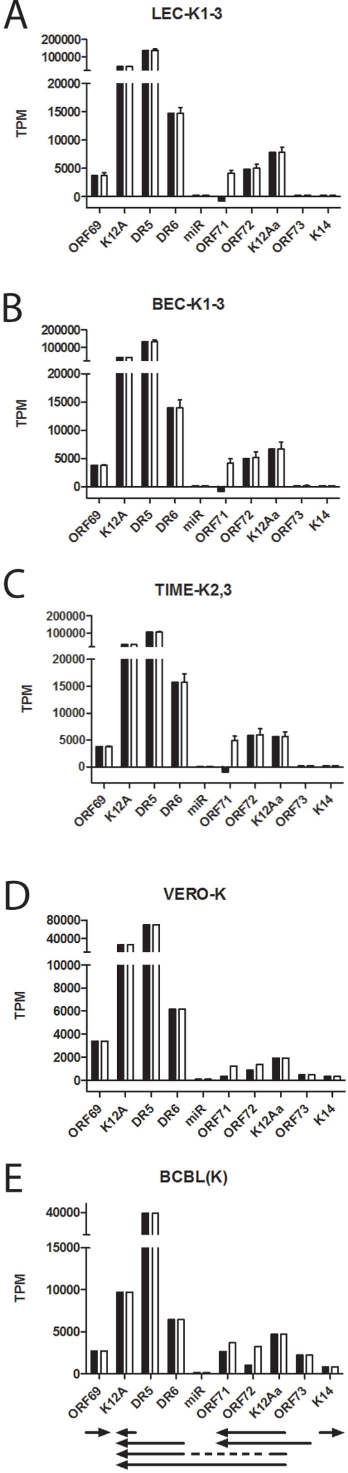

Figure S8. Quantitation of reads mapping to the primary transcripts of ORFs 69-K14. The total number of reads mapping to the UCDS feature for each ORF were quantitated by HTSEQ and normalized (TPM) with the average and standard deviation for the replicate infections (open bars). The number of reads mapping only to the primary transcript, in which the designated ORF is 5' proximal were quantitated by subtraction of the number of reads attributed to transcripts in which the designated ORF is distal in a polycistronic transcript (black bars; see Table 3 and Table S8). A) BEC-K1-3; B) LEC-K1-3; C) TIME-K2,3; D) VERO-K; E) BCBL(K). The proposed pattern of transcription is indicated at the bottom.
