# Supplementary material for: Quantitative Analysis of the KSHV Transcriptome Following Primary Infection of Blood and Lymphatic Endothelial Cells
Source: Pathogens. 2017 Mar 19;6(1):11. doi: 10.3390/pathogens6010011 (PMC5371899; doi:10.3390/pathogens6010011)
Supplement: Supplementary file 1 [file pathogens-06-00011-s001.zip › Table S1 - ORF6-K3 UCDS quantitation.pdf]

S1 Table: Comparison of total and primary transcripts encoding ORFs 6-K3[1]

UCDS Feature[2]	LEC-K1-3 (TPM)[3]		BEC-K1-3 (TPM)		TIME-K2-3 (TPM)		TIME-K1 (TPM)		BCBL(K) (TPM)		VERO-K (TPM)	
	Total[4]	Primary[5]	Total	Primary	Total	Primary	Total	Primary	Total	Primary	Total	Primary
ORF6	4239±12%	4239±12%	3997±10%	3997±10	5367±10%	5367±10%	10639	10639	1258	1258	5313	5313
ORF7	735±8%	735±8%	678±13%	678±13%	730±8%	730±8%	1271	1271	158	158	462	462
ORF8	2132±3%	1397±5%	2155±7%	1478±5%	2181±9%	1451±9%	2174	903	959	801	916	455
ORF9	2898±3%	766±5%	2996±4%	841±6%	3025±8%	844±6%	3521	1347	1436	477	1619	703
ORF10	3384±4%	486±19%	3447±3%	451±16%	3674±4%	649±16%	5161	1640	2459	1022	2577	958
ORF11	6912±7%	3528±17%	5975±7%	2528±18%	5549±3%	1875±17%	13143[6]	7982	7077	4618	3892	1315
K2	5207±8%	2720±20%	4890±10%	2211±15%	6536±7%	4331±14%	26165	23292	27919	27509	5957	3927
ORF2	2487±6%	2487±6%	2679±6%	2679±6%	2205±7%	2205±7%	2873	2873	410	410	2031	2031
K3	4331±5%	4331±5%	4220±10%	4220±10%	3446±1%	3446±1%	6100	6100	5094	5094	10844	10844

[1]RNA transcripts were quantitated using HTSEQ from RNA isolated from triplicate independent infections of LEC, BEC and TIME endothelial cells and single infections of VERO and BCBL-1 cells. The initial TIME cell infection (TIME-K1) is analyzed separately, as discussed in the text.

[2]UCDS features used by HTSEQ to quantitate RNAseq reads; see Table 3

[3]TPM=transcripts per million mapped KSHV reads

[4]Total = total reads mapped to the UCDS for all mono- and poly-cistronic transcripts

[5]Primary = Reads mapping only to the primary transcript of the 5' proximal ORF (see Table 3):
ORF6(primary)=ORF6(total); ORF7(primary)=ORF7(total); ORF8(primary)=ORF8(total)-ORF7(total);
ORF9(primary)=ORF9(total)-ORF8(total); ORF10(primary)=ORF10(total)-ORF9(total); ORF11(primary)=ORF11(total)-ORF10(total);
ORFK2(primary)=ORFK2(total)-ORF2(total); ORF2(primary)=ORF2(total); ORFK3(primary)=ORFK3(total).

[6]Comparatively higher read depths are highlighted in yellow
