# Supplementary material for: Quantitative Analysis of the KSHV Transcriptome Following Primary Infection of Blood and Lymphatic Endothelial Cells
Source: Pathogens. 2017 Mar 19;6(1):11. doi: 10.3390/pathogens6010011 (PMC5371899; doi:10.3390/pathogens6010011)
Supplement: Supplementary file 1 [file pathogens-06-00011-s001.zip › Table S10 - Modules of co-regulated genes.pdf]

S10 Table: Modules of co-regulated genes

Cluster1[1]	Cluster2[1]	Cluster3[1]	Cluster4[1]	Cluster5[1]	ENV/CAP 5 subcluster[1]
K14	K7	47	K4.1	27	8
73	4A	32	59	52	17.5
71	16	41	23	69	19
K9	58	K8a	20	DR6	65
OLAP	66	11	61	62	K8.1
DR2	K4	mIR	40A	63	K4.2A
K6	K3	LIR1	7	DR5	26
PAN	60	72	34	K12A	39
4B	70	K12Aa	42	33	25
DR1	64	37	29A	30	
K1	K4.2	57	2	53	
55	K5	6	46	K10.5	
38	74	36	44	K11	
49	56	9	43	8	
45		35	48	17.5	
K2		K15a		19	
50				65	
10				K8.1	
54				K4.2A	
				26	
				39	
				25	
				68	
				21	
				K10	
				18	
				67	
				24	
				22	
				17	
				31	
				67A	
				75	
				28	
				29B	

[1] Gene clusters identified by hierarchical clustering of gene-gene correlations, as shown in Figure 17.
