# Supplementary material for: Quantitative Analysis of the KSHV Transcriptome Following Primary Infection of Blood and Lymphatic Endothelial Cells
Source: Pathogens. 2017 Mar 19;6(1):11. doi: 10.3390/pathogens6010011 (PMC5371899; doi:10.3390/pathogens6010011)
Supplement: Supplementary file 1 [file pathogens-06-00011-s001.zip › Table S11 - Functional classification of KSHV genes.pdf]

Table S11: Functional classification of KSHV genes[1]

Env/ Memb	Capsid	Tegument	Virion	Regulatory	Replication	Immuno-Modulatory	Mitotic	Other
8	17.5	11	17	PAN	6	K1	K7	OLAP
K4.2A	25	19	20	18	7	4A	16	DR1
22	26	21	24	30	9	4B	K12A	DR2
23	43	32	29B	31	2	10	DR5	
27	62	33	29A	34	70	K2	DR6	
28	65	52	38	35	36	K3	71	
39	66	63	67A	37	40A	K4	72	
47		64	69	49	41	K4.1	K12Aa	
48				50	42	K4.2	73	
K8.1				K8a	44	K5	K15a	
53				55	46	K6		
58				57	54	45		
68					56	K9		
					59	K10		
					60	K10.5		
					61	K11		
						K14		
						74		

[1]KSHV genes, as represented by their related UCDS feature name, were classified based on their proposed function or mechanism of action from the published literature.
