# Supplementary material for: Quantitative Analysis of the KSHV Transcriptome Following Primary Infection of Blood and Lymphatic Endothelial Cells
Source: Pathogens. 2017 Mar 19;6(1):11. doi: 10.3390/pathogens6010011 (PMC5371899; doi:10.3390/pathogens6010011)
Supplement: Supplementary file 1 [file pathogens-06-00011-s001.zip › Table S12 - Significance of functional enrichment.pdf]

S12 Table: Significance of functional enrichment in gene regulatory modules[1]

<table>
<tr><th>Function</th><th>Cluster 1</th><th>Cluster 2</th><th>Cluster 3</th><th>Cluster 4</th><th>Cluster 5</th><th>Env/Cap subcluster</th></tr>
<tr><td>Envelope/ Membrane</td><td>P=0.1178</td><td>P=0.6855</td><td>P=0.6873</td><td>P=1</td><td>P=0.0113 (OR 5.0)[2]</td><td>P=0.01667 (OR 6.9)</td></tr>
<tr><td>Capsid</td><td>P=0.3432</td><td>P=1</td><td>P=0.595</td><td>P=1</td><td>P=0.0937 (OR 5.0)</td><td>P=0.0011 (OR 21.2)</td></tr>
<tr><td>Tegument</td><td>P=0.2002</td><td>P=1</td><td>P=0.6652</td><td>P=0.3523</td><td>P=0.0318 (OR 4.9)</td><td>P=1</td></tr>
<tr><td>Virion assembly/egress</td><td>P=1</td><td>P=0.597</td><td>P=0.3464</td><td>P=0.6042</td><td>P=0.1295 (OR 3.3)</td><td>P=1</td></tr>
<tr><td>Transcription Regulation</td><td>P=0.2248</td><td>P=0.2056</td><td>P=0.1042</td><td>P=0.6856</td><td>P=0.5293</td><td>P=0.5954</td></tr>
<tr><td rowspan="2">Replication</td><td rowspan="2">P=0.2907</td><td rowspan="2">P=0.6953</td><td>P=0.2898</td><td>P=0.0003 (OR 10.3)</td><td rowspan="2">P=0.0005</td><td rowspan="2">P=0.3477</td></tr>
<tr><td colspan="2">P=0.0001 (OR 9.6)</td></tr>
<tr><td rowspan="2">Immune modulation</td><td>P=0.0140 (OR 4.4)</td><td>P=0.0145 (OR 4.9)</td><td rowspan="2">P=0.0663</td><td rowspan="2">P=0.4574</td><td rowspan="2">P=0.1024</td><td rowspan="2">P=0.352</td></tr>
<tr><td colspan="2">P<0.0001 (OR 10.3)</td></tr>
<tr><td>Mitogenesis/ Cell cycle</td><td>P=1</td><td>P=0.632</td><td>P=0.2071</td><td>P=0.3523</td><td>P=1</td><td>P=0.5915</td></tr>
</table>

[1] Fisher's exact test for correlation between functional attributes of genes and gene regulatory modules identified in Figure 17.

[2]OR = Odds Ratio greater than 2.0 are shown
