# Supplementary material for: Quantitative Analysis of the KSHV Transcriptome Following Primary Infection of Blood and Lymphatic Endothelial Cells
Source: Pathogens. 2017 Mar 19;6(1):11. doi: 10.3390/pathogens6010011 (PMC5371899; doi:10.3390/pathogens6010011)
Supplement: Supplementary file 1 [file pathogens-06-00011-s001.zip › Table S13 - KSHV gene promoter categories.pdf]

S13 Table: KSHV gene promoter categories[1]

TATTTAA	TATTAAA	TATATA	TATAA	TATA	TAAAT	Undefined
8	4A	2	OLAP	6	K1	18
K2	K4.2A	16	K6	11	7	4B
K4.2	22	24	25	70	9	23
K5	26	28	30	K3	20	31
17.5	29B	35	46	55	21	34
17	33	42	49	56	29A	44
19	38	47	52	K11	50	45
27	K8.1	53	K8a	66	K4	58
32	64	54	K10	71		60
39	65	57	69			K10.5
40A	74	K9	K14			DR1
48		61	73			DR2
52						DR5
59						DR6
62						K12A
63						K12Aa
67						K4.1
67A						K7
K15a						LIR1
36						mIR
41						PAN
43						
68						
75						
37						
72						
K4.1						

[1]Promoter categories were derived from the promoter elements listed in Table 2
