# Supplementary material for: Quantitative Analysis of the KSHV Transcriptome Following Primary Infection of Blood and Lymphatic Endothelial Cells
Source: Pathogens. 2017 Mar 19;6(1):11. doi: 10.3390/pathogens6010011 (PMC5371899; doi:10.3390/pathogens6010011)
Supplement: Supplementary file 1 [file pathogens-06-00011-s001.zip › Table S14 - Significance of promoter enrichment.pdf]

Table S14: Significance of promoter enrichment in gene regulatory modules[1]

Promoter element[2]	Cluster 1	Cluster 2	Cluster 3	Cluster 4	Cluster 5
TATTTAAA	P=0.0358	P=0.1044	P=0.3534	P=0.5341	P=0.0962 (OR 2.3)[3]
TATTAAA	P=0.6833	P=0.1902 (OR 2.6)	P=0.2025	P=0.2062	P=0.0509 (OR 3.6)
TATATA	P=1	P=1	P=0.4082	P=0.3875	P=0.5293
TATAA	P=0.04141 (OR 3.9)	P=0.2056	P=0.684	P=0.6856	P=0.7504
TATA	P=0.6684	P=0.0228 (OR 6.1)	P=0.6376	P=0.347	P=0.1515
TAAAT	P=1	P=1	P=1	P=0.0703 (OR 4.8)	P=0.4159
Other	P=0.5276	P=1	P=1	P=0.7325	P=0.6083

[1] Fisher's exact test – see Materials and Methods

[2] see Table 2 for promoter elements

[3]OR = Odds Ratio greater than 2.0 are shown
