# Supplementary material for: Quantitative Analysis of the KSHV Transcriptome Following Primary Infection of Blood and Lymphatic Endothelial Cells
Source: Pathogens. 2017 Mar 19;6(1):11. doi: 10.3390/pathogens6010011 (PMC5371899; doi:10.3390/pathogens6010011)
Supplement: Supplementary file 1 [file pathogens-06-00011-s001.zip › Table S15 - Overexpressed UCDS gene features.pdf]

Table S15: Overexpressed KSHV genes[1]

TIME-K1	BCBL-1(K)	VERO-K	LEC-K1	LEC-K2	LEC-K3	BEC-K1	BEC-K2	BEC-K3
6	K1	K1		LIR1	LIR1	28	K4.2A	K4.1
7	4B	4A		K5	DR1	29B	23	31
9	10	4B		miR	68	31	28	67
10	11	K3				67	29B	
11	K2	70						
K2	DR1	K4						
K4.1	DR2	K4.1						
32	OLAP	K4.2						
29A	K6	DR1						
34	PAN	DR2						
35	32	OLAP						
36	38	K5						
37	45	K6						
41	49	K7						
46	50	23						
47	K8a	56						
50	54	58						
K8a	K9	60						
54	71	64						
56	73	66						
57	K14	74						
61								
mIR								
72								
K12Aa								
74								
K15a								

[1]Overexpressed KSHV genes were defined as those having expression levels in one infected cell culture that were outside of the 95% confidence level and 1.5 fold or greater than the mean of the expression levels in the other infected cell cultures. The UCDS gene features corresponding to the different KSHV genes are shown.
