# Supplementary material for: Quantitative Analysis of the KSHV Transcriptome Following Primary Infection of Blood and Lymphatic Endothelial Cells
Source: Pathogens. 2017 Mar 19;6(1):11. doi: 10.3390/pathogens6010011 (PMC5371899; doi:10.3390/pathogens6010011)
Supplement: Supplementary file 1 [file pathogens-06-00011-s001.zip › Table S16 - Significance of overexpression.pdf]

Table S16: Significance of enrichment of overexpressed genes in regulatory modules[1]

Infected Cell	Cluster 1	Cluster 2	Cluster 3	Cluster 4	Cluster 5
BCBL-1(K)	P<0.0001 (OR 347)[2]	P=0.06662	P=1	P= 0.0359	P<0.0001
VERO-K	P=0.5169	P<0.0001 (OR 128)	P=0.0367	P=0.729	P<0.0001
TIME-K1	P=0.772	P=0.3378	P<0.0001 (OR 82)	P=0.3451 (OR 2.0)	P<0.0001
TIME-K2	P=0.1178	P=0.2041	P=0.6873	P=0.2083	P<0.0001 (OR 31)
TIME-K3	P=1	P=1	P=0.3013 (OR 5.3)	P=0.284 (OR 5.7)	P=0.5361
BEC-K1	P=1	P=1	P=1	P=1	P=0.0145
BEC-K2	P=1	P=1	P=1	P=0.4913 (OR 1.9)	P=0.1286 (OR 5.7)
BEC-K3	P=1	P=1	P=1	P=1	P=1
LEC-K1	NA	NA	NA	NA	NA
LEC-K2	P=1	P=0.2666 (OR 6.2)	P=0.3013 (OR 5.3)	P=1	P=0.5361
LEC-K3	P=0.4598 (OR 2.3)	P=1	P=0.4177 (OR 2.6)	P=1	P=1

[1] Fisher's exact test was used to calculate the significance of enrichment (see Figure 17). Overexpressed genes are defined as those genes whose primary transcript levels (TPM) fall outside of the 95% confidence level and are 1.5 fold or greater than the mean of the other transcript levels.

[2]OR = Odds Ratios equal to or greater than 2.0 are shown
