# Supplementary material for: Quantitative Analysis of the KSHV Transcriptome Following Primary Infection of Blood and Lymphatic Endothelial Cells
Source: Pathogens. 2017 Mar 19;6(1):11. doi: 10.3390/pathogens6010011 (PMC5371899; doi:10.3390/pathogens6010011)
Supplement: Supplementary file 1 [file pathogens-06-00011-s001.zip › Table S2 - Pan region UCDS quantitation.pdf]

S2 Table: Comparison of total and primary transcripts encoding ORFs in the PAN region[1]

UCDS Feature[2]	LEC-K1-3 (TPM)[3]	BEC-K1-3 (TPM)	TIME-K2,K3 (TPM)	TIME-K1 (TPM)	BCBL(K) (TPM)	VERO-K (TPM)
K3	4331±5%	4220±10%	3446±0%	6100	5094	10844
ORF70	3407±6%	3261±13%	2751±0%	5084	2582	8239
K4	20088±4%	22877±2%	16272±1%	20912	26916	63647
K4.1[4]	555±78%[5]	781±44%	877±34%	1098	15	1483
K4.2A[6]	6372±10%	7896±12%	6922±16%	3128	1189	2534
K4.2[7]	149±16%	163±6%	102±22%	169	88	271
LIR1	117±32%	87±13%	59±28%	114	76	73
DR1	3032±40%	1890±16%	837±8%	722	6053	6150
DR2	1229±38%	737±10%	396±5%	190	4463	2435
OLAP	3108±35%	1884±8%	884±7%	608	14789	7759
K5	9145±21%	6549±13%	4463±11%	10723	7833	11963
K6	4537±23%	3848±10%	2271±4%	3322	13459	7630
K7[8]	2567±26%	1968±7%	1075±2%	622	2798	5694
PAN	429503±5%	435022±3%	465811±1%	327965	650560	540860
ORF16	586±13%	549±9%	506±10%	310	471	732
ORF17.5[9]	11554±2%	12777±5%	12659±2%	6274	7724	6473

[1]Primary RNA transcripts were quantitated using HTSEQ from RNA isolated from triplicate Independent infections of LEC, BEC and TIME endothelial cells and single infections of VERO and BCBL-1 cells, as described in Table 3. The initial TIME cell infection (TIME-K1) is analyzed separately, as discussed in the text.

[2]UCDS features used by HTSEQ to quantitate RNAseq reads; see Table 3

[3]TPM=transcripts per million mapped KSHV reads

[4]K4.1 primary transcript quantitation= K4.1 UCDS TPM (total) - K4.2A UCDS TPM (total) (see Table 3)

[5]TPM values with standard deviations above 15% are highlighted in yellow

[6]K4.2A = C-terminal region of K4.2 ORF common to short K4.2A and long K4.2 transcripts; K4.2A primary transcript quantitation=K4.2A UCDS TPM (total) – K4.2 UCDS TPM (total) (see Table 3)

[7]K4.2 = N-terminal region of K4.2 ORF only in long K4.2 transcript

[8]The K7 UCDS feature corresponds to the 5' untranslated region of the K7 transcript upstream of the PAN RNA.

[9]ORF17.5 = C-terminal region of ORF17 common to short ORF17.5 and long ORF17 transcripts; ORF17.5 primary transcript quantitation = ORF17.5 UCDS TPM (total) - ORF17 UCDS TPM (total)(see Table 3)
