# Supplementary material for: Quantitative Analysis of the KSHV Transcriptome Following Primary Infection of Blood and Lymphatic Endothelial Cells
Source: Pathogens. 2017 Mar 19;6(1):11. doi: 10.3390/pathogens6010011 (PMC5371899; doi:10.3390/pathogens6010011)
Supplement: Supplementary file 1 [file pathogens-06-00011-s001.zip › Table S3 - ORF17 UCDS quantitation.pdf]

S3 Table: UCDS quantitation of overlapping ORF17/ORF17.5 primary transcripts[1]

UCDS Feature[2]	LEC-K1-3 (TPM)[3]	BEC-K1-3 (TPM)	TIME-K2,3 (TPM)	TIME-K1 (TPM)	BCBL(K) (TPM)	VERO-K (TPM)
	Primary[4]	Primary	Primary	Primary	Primary	Primary
ORF16	586±13%	549±9%	506±9%	310	471	732
ORF17.5	11554±2%	12777±5%	12659±1%	6274	7724	6473
ORF17	1994±2%	2069±7%	1947±5%	1462	1276	1892
ORF18	1284±7%	1328±6%	1380±7%	1078	988	1336

[1]RNA transcripts were quantitated using HTSEQ from RNA isolated from triplicate independent infections of LEC, BEC and TIME endothelial cells and single infections of VERO and BCBL-1 cells. The initial TIME cell infection (TIME-K1) is analyzed separately, as discussed in the text.

[2]UCDS features used by HTSEQ to quantitate RNAseq reads; see Table 3

[3]TPM=transcripts per million mapped KSHV reads

[4]Primary = Reads mapping only to the primary transcript of the 5’ proximal ORF (see Table 3): ORF16(primary)=ORF16(total, ORF17.5(primary)=ORF17.5(total)-ORF17(total), ORF17(primary)=ORF17(total), ORF18(primary)=ORF18(total)
