# Supplementary material for: Quantitative Analysis of the KSHV Transcriptome Following Primary Infection of Blood and Lymphatic Endothelial Cells
Source: Pathogens. 2017 Mar 19;6(1):11. doi: 10.3390/pathogens6010011 (PMC5371899; doi:10.3390/pathogens6010011)
Supplement: Supplementary file 1 [file pathogens-06-00011-s001.zip › Table S4 - ORF49-52 UCDS quantitation.pdf]

S4 Table: Comparison of total and primary transcripts encoding ORFs 49-52[1]

UCDS Feature[2]	LEC-K1-3 (TPM)[3]		BEC-K1-3 (TPM)		TIME-K2-3 (TPM)		TIME-K1 (TPM)		BCBL(K) (TPM)		VERO-K (TPM)	
	Total[4]	Primary[5]	Total	Primary	Total	Primary	Total	Primary	Total	Primary	Total	Primary
ORF49	1178±8%	1178±8%	1269±16%	1269±16%	1207±21%	1207±12%	1412	1412	2177	2177	884	884
ORF50	1479±8%	1479±8%	1730±6%	1730±6%	1516±15%	1516±15%	2477	2477	2535	2535	1470	1470
ORFK8	6295±14%	4816±20%	6596±18%	4866±25%	7841±6%	6324±11%	24601	22124	12701	10166	2753	1283
ORFK8.1	30862±8%	30862±8%	31696±13%	31696±13%	37000±9%	37000±9%	14859	14859	4686	4686	5401	5401
ORF52	6636±3%	6636±3%	6730±12%	6730±12%	6854±10%	6854±10%	3058	3058	6246	6246	4379	4379

[1]RNA transcripts were quantitated using HTSEQ from RNA isolated from triplicate independent infections of LEC, BEC and TIME endothelial cells and single infections of VERO and BCBL-1 cells. The initial TIME cell infection (TIME-K1) is analyzed separately, as discussed in the text.

[2]UCDS features used by HTSEQ to quantitate RNAseq reads; see Table 3

[3]TPM=transcripts per million mapped KSHV reads

[4]Total = total reads mapped to the UCDS features for all mono- and poly-cistronic transcripts

[5]Primary = Reads mapping only to the primary transcript of the 5' proximal ORF; ORF49(primary)=ORF49(total); ORF50(primary)=ORF50(total); ORFK8(primary)=ORFK8(total)-ORF50(total); ORFK8.1(primary)=ORFK8.1(total); ORF52(primary)=ORF52(total)
