# Supplementary material for: Quantitative Analysis of the KSHV Transcriptome Following Primary Infection of Blood and Lymphatic Endothelial Cells
Source: Pathogens. 2017 Mar 19;6(1):11. doi: 10.3390/pathogens6010011 (PMC5371899; doi:10.3390/pathogens6010011)
Supplement: Supplementary file 1 [file pathogens-06-00011-s001.zip › Table S5 - ORF44-50 UCDS quantitation.pdf]

S5 Table: Comparison of total and primary transcripts encoding ORFs 44-50

	LEC-K1-3		BEC-K1-3		TIME-K2,3		TIME-K1		BCBL(K)		VERO-K	
	Total[1]	Primary[2]	Total	Primary	Total	Primary	Total	Primary	Total	Primary	Total	Primary
ORF44	531±11%	531±11%	556±11%	556±11%	477±10%	477±10%	628	628	377	377	400	400
ORF45	5719±5%	2119±6%	6020±7%	2240±7%	5970±6%	2240±18%	6131	1382	6280	4059	4212	1681
ORF46	3600±8%	1149±19%	3780±3%	1179±3%	3730±2%	1004±7%	4749	1553	2220	-245	2530	850
ORF47	2451±4%	1202±6%	2601±7%	1232±7%	2726±5%	1460±28%	3196	1806	2465	1600	1681	570
ORF48	1249±7%	1249±7%	1369±15%	1369±15%	1266±7%	1266±22%	1391	1391	865	865	1111	1111
ORF49	1178±8%	1178±8%	1269±16%	1269±16%	1207±7%	1207±21%	1412	1412	2177	2177	884	884
ORF50	1479±8%	1479±8%	1730±6%	1730±6%	1516±7%	1516±15%	2477	2477	2535	2535	1470	1470

[1]Total = total reads (TPM) mapped to the UCDS for all mono- and polycistronic transcripts

[2]Primary = Reads (TPM) mapping only to the primary transcript of the 5' proximal ORF (as described in Table 3);
ORF44(primary)=ORF44(total); ORF45(primary)=ORF45(total)-ORF46(total); ORF46(primary)=ORF46(total)-ORF47(total);
ORF47(primary)=ORF47(total)-ORF48(total); ORF48(primary)=ORF48(total); ORF49(primary)=ORF49(total).
