# Supplementary material for: Quantitative Analysis of the KSHV Transcriptome Following Primary Infection of Blood and Lymphatic Endothelial Cells
Source: Pathogens. 2017 Mar 19;6(1):11. doi: 10.3390/pathogens6010011 (PMC5371899; doi:10.3390/pathogens6010011)
Supplement: Supplementary file 1 [file pathogens-06-00011-s001.zip › Table S6 - Quantitation of sense-antisense transcripts.pdf]

S6 Table: Quantitation of sense and antisense transcripts[1]

ORFs[2]	BEC-K1 (TPM)[3]	LEC-K1 (TPM)	TIME-K1 (TPM)	BCBL(K) (TPM)
K2 total[4]	4574	5412	22665	27961
K2 AS[5]	13	14	9	17
K2 Sense[6]	4561	5397	22656	27944
% sense of total	99.7%	99.7%	100.0%	99.9%
K4 total	16998	16453	15550	22268
K4 AS[7]	8	12	0	14
K4 Sense	16991	16442	15550	22255
% sense of total	100.0%	99.9%	100.0%	99.9%
DR1 total	1095	897	320	6063
DR1 AS	536	434	155	2588
DR1 Sense	558	462	165	3475
% sense of total	51.0%	51.6%	51.4%	57.3%
DR2 total	525	392	56	4469
DR2 AS	10	2	0	20
DR2 Sense	516	390	56	4449
% sense of total	98.1%	99.5%	100.0%	99.5%
K6 total	2793	2351	2501	13479
K6 AS	1520	1152	257	1362
K6 Sense	1274	1199	2243	12117
% sense of total	45.6%	51.0%	89.7%	89.9%
29A total	1177	980	1329	307
29A AS	1083	901	1286	234
29A Sense	94	78	43	73
% sense of total	8.0%	8.0%	3.3%	23.9%
29B total	2581	1815	817	452
29B AS	2465	1682	672	315
29B Sense	116	133	145	137
% sense of total	4.5%	7.3%	17.8%	30.4%
ORF50 total	1507	1103	2317	2539
ORF50 AS	468	391	778	1150
ORF50 sense	1040	712	1540	1389
% sense of total	69.0%	64.6%	66.4%	54.7%
ORF59 total	21245	21902	28799	14901
ORF59 AS	13	8	0	11
ORF59 sense	21232	21894	28799	14890
% sense of total	99.9%	100.0%	100.0%	99.9%
ORF68 total	1762	1457	1092	1199
ORF68 AS	1347	1178	611	750
ORF68 Sense	415	279	481	449
% sense of total	23.6%	19.2%	44.1%	37.4%
ORF69 total	3624	2917	2907	2729
ORF69 AS	1139	895	501	493
ORF69 Sense	2485	2022	2406	2236
% sense of total	68.6%	69.3%	82.8%	81.9%
K12Aa total	2839	2867	10792	4723
K12Aa AS	39	15	50	384
K12Aa sense	2800	2852	10742	4339
% sense of total	98.6%	99.5%	99.5%	91.9%
ORF72 total	5176	5457	15813	3291
ORF72 AS	28	30	27	193
ORF72 sense	5148	5427	15785	3098
% sense of total	99.5%	99.4%	99.8%	94.1%
ORF73 total	182	160	389	2130
ORF73 AS	37	30	25	273
ORF73 sense	145	130	364	1857
% sense of total	79.7%	81.3%	93.5%	87.2%

[1] Sense and antisense RNA transcripts were quantitated using HTSEQ on RNA reads from RNAseq stranded libraries of single infections of LEC, BEC and TIME endothelial cells and VERO and BCBL-1 cells.
[2] UCDS features used by HTSEQ to quantitate RNAseq reads; see Table 3
[3] TPM=transcripts per million mapped KSHV reads
[4] total number of reads mapping to UCDS feature
[5] number of reads mapping antisense to UCDS feature
[6] number of reads mapping to UCDS feature (sense strand)
[7] a similar lack of reads mapping antisense to the UCDS features of ORFK4.1 and ORFK4.2 was also observed
