# Supplementary material for: Quantitative Analysis of the KSHV Transcriptome Following Primary Infection of Blood and Lymphatic Endothelial Cells
Source: Pathogens. 2017 Mar 19;6(1):11. doi: 10.3390/pathogens6010011 (PMC5371899; doi:10.3390/pathogens6010011)
Supplement: Supplementary file 1 [file pathogens-06-00011-s001.zip › Table S7 - ORF58-62 UCDS quantitation.pdf]

S7 Table: Comparison of total and primary transcripts encoding ORFs 58-62

	LEC-K1-3 (TPM)		BEC-K1-3 (TPM)		TIME-K2,3 (TPM)		TIME-K1 (TPM)		BCBL(K) (TPM)		VERO-K (TPM)	
	Total[1]	Primary[2]	Total	Primary	Total	Primary	Total	Primary	Total	Primary	Total	Primary
ORF58	20331±6%	-1257±52%[3]	20541±7%	-1130±38%	23620±4%	-1794±19%	27736	-935	11817	-3061	26270	794
ORF59	21588±8%	13443±9%	21670±7%	13687±8%	25414±5%	15166±7%	28672	15125	14879	12165	25476	17502
ORF60	8145±7%	2015±10%	7983±6%	1978±10%	10248±2%	2640±4%	13547	2968	2714	1604	7974	4233
ORF61	6130±7%	6130±7%	6006±5%	6006±5%	7607±1%	7607±1%	10579	10579	1110	1110	3741	3741
ORF62	2494±8%	2494±8%	2413±6%	2413±6%	3248±5%	3248±5%	1796	1796	648	648	792	792

[1]Total = total reads mapped to the UCDS for all mono- and poly-cistronic transcripts; TPM= transcripts per million

[2]Primary = Reads mapping only to the primary transcript of the 5' proximal ORF: ORF58(primary)=ORF58(total)-ORF59(total); ORF59(primary)=ORF59(total)-ORF60(total); ORF60(primary)=ORF60(total)-ORF61(total); ORF61(primary)=ORF61(total); ORF62(primary)=ORF62(total).

[2]Negative values (red) for primary transcripts indicates the absence of a primary monocistronic transcript for ORF58 and the presence of an ORF59 transcript upstream that terminates before ORF58.
