# Supplementary material for: Quantitative Analysis of the KSHV Transcriptome Following Primary Infection of Blood and Lymphatic Endothelial Cells
Source: Pathogens. 2017 Mar 19;6(1):11. doi: 10.3390/pathogens6010011 (PMC5371899; doi:10.3390/pathogens6010011)
Supplement: Supplementary file 1 [file pathogens-06-00011-s001.zip › Table S8 - Latency locus UCDS quantitation.pdf]

S8 Table: Comparison of total and primary transcripts encoding latency region ORFs

UCDS[1]	LEC-K1-3 (TPM)		BEC-K1-3 (TPM)		TIME-K2,3 (TPM)		TIME-K1 (TPM)		BCBL(K) (TPM)		VERO-K (TPM)	
	Total[2]	Primary[3]	Total	Primary	Total	Primary	Total	Primary	Total	Primary	Total	Primary
ORF69	3694±14%	3694±14%	3817±3%	3817±3%	3765±4%	3765±4%	2820	2820	2725	2725	3403	3403
K12A[4]	43101±4%	43101±4%	41547±1%	41547±1%	32442±2%	32442±2%	40250	40250	9743	9743	26297	26297
DR5[5]	136194±6%	136194±6%	132412±7%	132412±7%	105200±4%	105200±4%	124092	124092	38833	38833	69959	69959
DR6[6]	14709±7%	14709±7%	13970±10%	13970±10%	15726±10%	15726±10%	13020	13020	6473	6473	6152	6152
miR[7]	204±31%	204±31%	142±13%	142±13%	107±11%	107±11%	291	291	169	169	96	96
ORF71	4072±13%	-722±31%[8]	4127±20%	-843±30%	4852±19%	-978±21%	12581	-2416	3713	2663	1213	338
ORF72	4995±15%	4795±16%	5176±20%	4971±22%	6013±18%	5830±19%	15308	14997	3286	1051	1369	875
K12Aa[9]	7817±12%	7817±12%	6656±19%	6656±19%	5627±16%	5627±16%	24204	24204	4716	4716	1919	1919
ORF73	200±6%	200±6%	205±16%	205±16%	183±11%	183±11%	311	311	2235	2235	494	494
K14	222±3%	222±3%	209±7%	209±7%	192±2%	192±2%	166	166	864	864	327	327

[1]UCDS feature used by HTSEQ to quantitate RNAseq reads; see Table 3

[2]Total = total reads mapped to the UCDS for all mono- and poly-cistronic transcripts; TPM=transcripts per million

[3]Primary = reads mapping only to the primary transcript of the 5' proximal ORF; due to the complexity of splicing in this region only restricted primary transcripts were quantitated: ORF69(primary)=ORF69(total), K12A(primary)=K12A(total), DR5(primary)=DR5(total), DR6(primary)=DR6(total), miR(primary)=miR(total), ORF71(primary)=ORF71(total)-ORF72(total), ORF72(primary)=ORF72(total)-ORF73(total), K12Aa(primary)=K12Aa(total), ORF73(primary)=ORF73(total), K14(primary)=K14(total)

[4]K12A = UCDS feature corresponding to the K12A Kaposin transcript

[5]DR5 = direct repeat 5 corresponding to the middle of the K12B/C transcript

[6]DR6= direct repeat 6 corresponding to the 5' end of the K12B/C transcript

[7]miR = region containing the microRNA genes –transcripts mapping to this region have not been processed to yield the mature micro RNAs

[8]Negative values (red) for primary transcripts indicate the absence of a primary monocistronic transcript for ORF71 and the presence of an ORF72 transcript upstream that terminates before ORF71.

[9]K12Aa = UCDS feature corresponding to the 5' exon immediately upstream of the ORF72 CDS
