# Supplementary material for: Quantitative Analysis of the KSHV Transcriptome Following Primary Infection of Blood and Lymphatic Endothelial Cells
Source: Pathogens. 2017 Mar 19;6(1):11. doi: 10.3390/pathogens6010011 (PMC5371899; doi:10.3390/pathogens6010011)
Supplement: Supplementary file 1 [file pathogens-06-00011-s001.zip › Table S9 - Quantitation of K12Aa-DR6 reads .pdf]

S9 Table: Quantitation of reads spanning splice junctions in the latency region[1]

Spliced intron[2]	LEC-K1-3	BEC-K1-3	TIME-K2,3	TIME-K1	VERO-K	BCBL(K)
5'ORF72-DR6 (a)	1179±29%	1094±10%	612±8%	496	49	71
5'ORF73-5'ORF72 (b)	7±71%	6±82%	7±100%	0	0	0
5'ORF73-DR6 (c)	0	0	0	0	0	0
5'ORF72-5'ORF71 (d)	0	0	0	0	0	0
5'ORF73-ORF73 (long) (e)	2±41%	6±82%	4±100%	0	0	0
5'ORF73-ORF73 (short) (f)	0	1±141%	1±100%	0	0	0
DR6-ORF67A[3]	98±19%	96±7%	44±20%	0	13	0
DR6-ORF66[3]	86±32%	94±8%	28±100%	0	0	4
K14-classical[3]	19±16%	28±6%	30±22%	0	11	0
K15-7 intron average[3]	187±15%	86±6%	108±1%	96	0	0

[1]RNA-seq reads spanning splice junctions from triplicate independent infections of LEC, BEC, and TIME endothelial cells and single infections of Vero and BCBL-1 cells were quantitated using TopHat2. Average and standard deviation were determined for replicate infections.

[2]introns shown in Fig. 10C

[3]not shown in Fig. 10C. Transcripts containing the DR6-ORF67A and DR6-ORF66 splices would have alternate 3' ends and secondary distal ORFs. The K14 classical splice junction spans the intron between 12,9369 and 12,9516 bp, as indicated in the KSHV RefSeq NC_009333. The reads spanning the splice junctions in K15 were determined as an average of the split reads across all 7 K15 introns.
